# Effects of Remotely Delivered and Web-Based Interventions on Depression Severity During the COVID-19 Pandemic: 3-Arm Randomized Controlled Trial

**DOI:** 10.2196/88388

**Published:** 2026-08-04

**Authors:** Zev Schuman-Olivier, Liv Valö, Joseph A Rosansky, Gareth Parry, Alexandra Comeau, Fiona Kate Rice, Carl Fulwiler

**Affiliations:** 1 Department of Psychiatry, Center for Mindfulness and Compassion, Cambridge Health Alliance, 350 Main Street, Suite 5126, Malden, MA, 02148, United States, 1 617-591-6056; 2 Department of Psychiatry, Harvard Medical School, Boston, MA, United States

**Keywords:** digital health, eHealth, telehealth, depression, mindfulness, Mindfulness-Based Cognitive Therapy, cognitive behavioral therapy, randomized controlled trial, population mental health, stratified care, COVID-19 pandemic

## Abstract

**Background:**

The COVID-19 pandemic highlighted a critical need for effective population mental health approaches to target the most prevalent disorders (eg, depression) during periods of elevated community distress. The effectiveness of remotely delivered and web-based interventions should be investigated to identify and innovate high-quality models for population mental health service delivery.

**Objective:**

The primary objective investigated the effectiveness of adding Mindfulness-Based Cognitive Therapy for Resilience (MBCT-R)—a live, online, synchronous, remotely delivered, group-based intervention—to Cambridge Health Alliance MindWell (CHA-MW), a web-based population health screening and stratified support program, compared with CHA-MW alone, on depression symptom severity. The secondary objective evaluated adding internet Cognitive Behavioral Therapy (iCBT)—an asynchronous, web-based, individual, digital intervention—to CHA-MW, compared with CHA-MW alone.

**Methods:**

Participants (N=97) were randomized in a 2:2:1 ratio to receive MBCT-R+CHA-MW (n=37), iCBT+CHA-MW (n=41), or CHA-MW alone (n=19) in a 3-arm randomized clinical trial, from May 2021 to September 2022 in an urban public safety net hospital outpatient setting. CHA-MW served as a low-intensity control condition. For the MBCT-R+CHA-MW arm, MBCT-R was an 8-session program mildly adapted from MBCT to address COVID-19–related risks for depression. For the iCBT+CHA-MW arm, iCBT was a 6-session curriculum added to CHA-MW. All study procedures, including regular mental health symptom screenings, were conducted remotely or via a web-based platform. The primary outcome was change in depression symptom severity during the 24-week study period using an intention-to-treat approach that used generalized linear mixed-effects models to evaluate the comparative effectiveness of MBCT-R+CHA-MW vs CHA-MW over time. A secondary analysis compared iCBT+CHA-MW vs CHA-MW on depression severity. Completer analyses were conducted (per-protocol 6+ sessions). The secondary outcome was mental health visit utilization frequency during the study period.

**Results:**

Both MBCT-R+CHA-MW (mean difference −14.1, 95% CI −21.0 to −7.2) and CHA-MW (mean difference −15.2, 95% CI −21.8 to −8.6) had significant reductions in depression symptom severity, with no statistically significant between-group differences. iCBT+CHA-MW (mean difference −12.7, 95% CI −17.4 to −8.1) also reduced depression symptoms but without between-group differences when compared with CHA-MW. Intervention completion rates were low (MBCT-R: 30% and iCBT: 24%), and completers demonstrated significantly greater reductions in depression severity than noncompleters (mean difference −8.5, 95% CI −16.2 to −0.8). Overall mental health clinician visits by group had no statistically significant differences. CHA-MW had the largest increase in participants with new psychopharmacology treatment visits during the 24-week study (CHA-MW +21%, MBCT-R +10%, and iCBT −5%).

**Conclusions:**

MBCT-R+CHA-MW, iCBT+CHA-MW, and CHA-MW were each effective in treating depression, without any intervention demonstrating superiority in intention-to-treat analyses. CHA-MW was as efficacious during the COVID-19 pandemic as more resource-intensive interventions that demanded greater time and effort from participants. Low completion rates for MBCT-R and iCBT during the COVID-19 pandemic may have contributed to these results.

## Introduction

The COVID-19 pandemic profoundly impacted global behavioral health, with significant implications for mental health, treatment seeking, and treatment capacity [1,2]. Consistent with a broad literature attesting to the linkage between natural disasters and increased rates of mental illness and treatment demand, the COVID-19 pandemic demonstrated outcomes similar to those seen after Hurricane Katrina (2005) [3-7] and the Baton Rouge flood (2016) [8-11] but on an international scale [2,12-16]. In the United States, rates of depression and anxiety disorders increased 3-fold, with the burden of care falling largely on primary care settings that were swiftly overwhelmed by the rising demand [17-19]. Furthermore, health disparities research revealed that individuals experiencing greater socioeconomic impacts tended to demonstrate higher risk for developing depression [18,20]. Taken together, these findings highlight the need during periods of high community distress for effective remotely delivered population mental health approaches that readily target the most prevalent disorders among vulnerable populations (eg, depression) in anticipation of the expected increases in mental health treatment demand [14].

Within months of the COVID-19 pandemic’s onset in 2020, Cambridge Health Alliance (CHA), an extensive public safety-net health care system in the Metro Boston area, launched a remotely delivered online population mental health screening and stratified behavioral health care program, CHA MindWell (CHA-MW) [21]. CHA-MW aimed to reduce the mental health impacts of the COVID-19 pandemic on vulnerable populations both during and after the acute crisis. The CHA-MW team designed the program to accomplish this through remote screening and monitoring of mental health symptoms and by offering participants a variety of online mental wellness resources, individual check-ins with telehealth mental wellness counselors, and treatment referrals to standard telemental health care services based on the severity of the symptoms reported. Specifically, CHA-MW used Computer Adaptive Testing for Mental Health (CAT-MH) [22,23] to monitor participants’ mental health via regular dynamically adapted behavioral health surveys. A retrospective single-arm evaluation of the first year of CHA-MW implementation found significant reductions in depression (*d*=−0.30), anxiety (*d*=−0.27), and posttraumatic stress disorder (PTSD) (*d*=−0.31) symptoms after 3‐6 months of program engagement during the COVID-19 pandemic [21]. Participants whose symptoms met or exceeded clinical thresholds at baseline displayed greater improvements in depression (*d*=−0.42), anxiety (*d*=−0.44), and PTSD (*d*=−0.37) [21].

Having established CHA-MW as a basic population health monitoring and response program for reducing depression, anxiety, and PTSD, CHA sought to further address the need for remotely delivered mental health interventions by augmenting existing CHA-MW services with other evidence-based treatments for depression and pandemic-related distress. Mindfulness-Based Cognitive Therapy (MBCT) for depression [24] was identified as a promising program for investigation based upon its demonstrated effectiveness for treating depression, anxiety, and stress, as well as its ability to be mildly adapted to meet present needs (eg, remote delivery and transdiagnostic focus) while maintaining fidelity to its theory and core techniques. As a cost-effective, evidence-based intervention for treatment and relapse prevention of depression, MBCT integrated mindfulness skills training with elements of cognitive therapy [25,26]. Several randomized controlled trials found that MBCT significantly reduced depression relapse rates compared with usual care [27-29]. Furthermore, MBCT had been found to be as effective for maintenance as antidepressant medication [27,30,31], with some studies finding greater comparative effectiveness, particularly for patients with residual depression symptoms [31,32]. While MBCT was originally designed to prevent relapse in patients with remitted symptoms, several studies have demonstrated its effectiveness among patients with active depressive disorders or elevated depressive symptoms [33-35], with a recent meta-analysis finding that MBCT was as effective as other active treatments for people with current depressive symptoms [36]. MBCT was also found to be effective for treating anxiety [37-39] and stress [40,41] when delivered in online or virtual and in-person formats [39,42,43].

Based upon this literature, the multiple principal investigator (MPI; CF) designed this study’s primary intervention of interest, Mindfulness-Based Cognitive Therapy for Resilience (MBCT-R), to maintain fidelity to MBCT’s fundamental theory and techniques while mildly adapting it to the postpandemic setting in 2 ways. First, the format was adapted so that group leaders could offer it to larger groups through videoconference to meet the imminent need for remotely delivered treatment. Second, based upon MBCT’s established effectiveness in treating anxiety and stress in addition to depression, the MPI expanded protocol language referring specifically to depressive symptoms to also include the types of stress, anxiety, and mood symptoms activated by the pandemic. Beyond these adaptations, the design and content of MBCT-R mapped directly onto that of its base intervention, MBCT, including synchronous delivery in a group format over 8 weekly 2.25-hour sessions (Table S1 in Multimedia Appendix 1).

While prior research established MBCT’s effectiveness in treating stress, anxiety, and depression during regular nonpandemic contexts [36-41], limited information was available about MBCT’s potential effectiveness during a pandemic with forced isolation [44]. Because social isolation could lead to loneliness, a risk factor for worsening depression [45], a group-based remotely delivered intervention such as MBCT-R had the potential to provide a protective benefit of social support. Therefore, the study’s main objective was to examine whether adding an evidence-based, live online, synchronous, remotely delivered, and group-based mindfulness intervention (MBCT-R) to CHA-MW would have additive benefits during the COVID-19 pandemic. The primary goal of this 3-arm randomized clinical trial was to compare the effectiveness of MBCT-R plus CHA-MW (MBCT-R+CHA-MW; arm 1) with CHA-MW (arm 3) on reducing depression symptom severity over 24 weeks (primary outcome). We hypothesized that MBCT-R+CHA-MW, as a specific and active treatment, would be more effective in reducing depressive symptoms than CHA-MW, a nonspecific, low-intensity, and resource-efficient comparator.

The study also included a secondary intervention of interest, an evidence-based form of internet Cognitive Behavioral Therapy (iCBT), to facilitate a secondary analysis that could examine the effects of adding a different remotely delivered treatment method that was individual, digital, and asynchronous, providing treatment without social support. As a secondary aim related to depression outcomes, the study sought to compare the effectiveness of iCBT+CHA-MW (arm 2) with CHA-MW (arm 3) on change in depression symptom severity over 24 weeks. We again hypothesized that iCBT+CHA-MW, as an active treatment, would be more effective in reducing depressive symptoms than CHA-MW alone.

The study also examined the potential impact of treatment on psychiatric visit utilization during the 24-week study period (secondary outcome), based upon prior research finding that MBCT reduced outpatient psychiatry service utilization [46,47]. We predicted that participants engaged in MBCT-R+CHA-MW would have fewer mental health clinician visits over the 24-week study period than those in CHA-MW alone. Sensitivity analyses were also planned to explore differential impacts of psychiatric visit utilization on the primary outcome (depression).

## Methods

### Design and Setting

This remotely delivered randomized clinical trial, conducted in a community health care system in the Metro-North Boston region during the COVID-19 pandemic, compared the effectiveness of MBCT-R supported by CHA-MW (MBCT-R+CHA-MW) with CHA-MW alone on change in depression symptoms over 24 weeks. As a secondary analysis, the study investigated the effectiveness of iCBT+CHA-MW versus CHA-MW alone on change in depression symptoms. Within- and between-group effects for depression were assessed and compared at 24 weeks. As a secondary outcome, participants’ behavioral health visit use during the 24-week study period was analyzed to investigate between-group differences as well as sensitivity analyses to measure the potential effects of visit use on the measured mental health outcomes.

### Ethical Considerations

This study was conducted in accordance with the ethical principles of the World Medical Association’s Declaration of Helsinki, as revised in 2024. All participants provided written informed consent before participating in the study. The CHA Institutional Review Board (reference number: Legacy-CHA-IRB-1141-05-20) approved the study, and the rights, privacy, and confidentiality of all participants were protected throughout the study. This study was preregistered on ClinicalTrials.gov (NCT04595084) and monitored by the National Center for Complementary and Integrative Health (NCCIH) and a NCCIH-approved Independent Monitoring Committee [48]. Participants received financial compensation, up to US $160, for their participation. Payment was given at 5 timepoints, including US $20 after the screening or consent visit, US $20 after completion of baseline surveys, US $60 after the week 12 assessment, US $40 after the week 24 assessment, and US $20 for a completion bonus. Upon completion, participants could also become eligible for an optional substudy on stress reactivity (not reported here), which included an additional 3 payment timepoints: US $20 for a second screening or consent following main study completion, US $20 to US $40 for saliva collection, and US $20 to US $40 for pre– and/or post–daily diaries.

### Participants

Participants were recruited through 2 main methods. First, emails were sent to all adult primary care patients in the health care system inviting them to participate in CHA-MW [21]. Second, primary care providers referred patients with anxiety, depression, or COVID-19–related distress to CHA-MW. Once individuals registered for CHA-MW, the CHA-MW team automatically invited them to complete the first CAT-MH assessment. Those who met preliminary study inclusion criteria via CAT-MH symptom severity scores were eligible and invited by the CHA-MW team to learn more about joining this study. Study research coordinators screened all patients who expressed interest in joining the study for eligibility through a standardized process that included telephone interviews, self-report surveys, and electronic health record review. Inclusion criteria required participants to be current CHA patients aged between 18 and 70 years with English proficiency, able to participate in teleconference meetings, and having a depression score of 50‐75 measured via CAT-MH Depression module (CAT-DEP) [49], representing mild to moderately severe depression symptoms. Exclusion criteria included psychosis (CAT-Psychosis>60) [50], history of bipolar I disorder or current mania (CAT-Mania>70) [51], acute suicidality (CAT-Suicide ≥71) [52], severe depression (CAT-DEP>75) [49,51], severe PTSD (CAT-PTSD>75) [51], severe substance use disorder or positive toxicology for cocaine, unprescribed opioids, stimulants, or benzodiazepines in the 3 months before screening, current treatment with antipsychotic medication, mood stabilizer or benzodiazepine equivalent of 3 mg per day of lorazepam, current participation in another experimental research study, mindfulness group experience within the past year, or anticipated hospitalization or incarceration in the next 6 months. Eligible participants reviewed informed consent via REDCap (Research Electronic Data Capture) [53] and completed a consent assessment to ensure that they understood key aspects of study procedures. Those who scored greater than 90% completed the consent process and were able to complete baseline surveys. Study reporting followed the CONSORT (Consolidated Standards of Reporting Trials). The trial protocol is provided in Multimedia Appendix 2.

### Randomization and Blinding

Upon completion of the baseline survey battery, participants were randomized in randomly ordered blocks of 5 or 10 with a 2:2:1 ratio to MBCT-R+CHA-MW, iCBT+CHA-MW, or CHA-MW according to a computer-generated sequence (STATA random generator). The study methodologist (GP) provided randomization lists to the study senior research coordinator (AC) and/or data analyst (JB), all of whom were blinded to participant identity prior to and during randomization. When a participant was eligible, research coordinators informed the senior research coordinator, data analyst, or the methodologist who, in turn, provided the randomization status, as research coordinators themselves did not have access to source the blinded randomization sequence. A research coordinator then called the participant by phone to inform them of the process and timing for the MBCT-R or iCBT program, as well as the process for ongoing CHA-MW surveys.

Participants were not blinded to their assigned study arm, nor were the coprimary investigator (CF), research coordinators, data analyst, or methodologists after randomization took place. Finally, the contact primary investigator (ZS-O) was blinded to the assigned study arm when reviewing data quality, adverse events, and protocol violations, and was not fully unblinded until all primary outcome data were collected and the database was locked.

### Interventions

All interventions were delivered between baseline and study week 8. CHA-MW monitoring and support was provided to all participants, while the other 2 interventions were added to CHA-MW.

#### CHA-MW Monitoring and Support

CHA-MW [21] was an online population health monitoring and support platform wherein participants completed regular behavioral health surveys that enabled health care providers to screen for, track, and respond to clinical concerns. Designed to be a foundational component within a stepped-care framework, CHA-MW provided a low-intensity comparator to MBCT-R+CHA-MW by offering a baseline level of participant engagement that exceeded usual care, while also being more resource-efficient than alternative active controls used in comparative mindfulness-oriented interventional studies (eg, cognitive behavioral therapy, psychoeducation, and health educational programs) [36]. Participants across all study arms were provided with CHA-MW monitoring with surveys that dynamically adapted based upon participant responses, measuring depression (CAT-DEP) and anxiety (CAT-MH Anxiety module [CAT-ANX]) symptoms, respectively, weekly during weeks 1‐8 and then with completion of the full CAT-MH (including depression, anxiety, mania and hypomania, PTSD, psychosis, suicidality, and substance use risk) every 4 weeks for the 24-week study period.

CHA-MW included both general online mental wellness resources and targeted mental wellness coaching support based on a stratified care algorithm activated by the CAT-MH monitoring. For example, the CHA-MW team sent out a monthly newsletter that focused on a different psychoeducational topic each month (eg, sleep, winter blues, effects of food on mood, and coping with anxiety) and suggested a relevant smartphone app (using an app tool kit with free health care system–vetted smartphone apps [54]) that matched the newsletter theme for that month. In addition to the newsletter, which encouraged completion of the monthly CAT-MH for symptom monitoring, CHA-MW used a stratified care algorithm that, if depression worsened or was not improving after 2 weeks, resulted in activation of individual televisit check-ins with mental wellness counseling and/or with psychiatry treatment referrals to standard telemental health care services as indicated. Trained support technicians reviewed scores weekly using semiautomated algorithms and a REDCap dashboard [55], which delivered a scheduling link to participants whose CAT-MH scores met or exceeded clinical thresholds, including participants whose scores (1) increased by more than 5 points on the CAT-DEP Patient Health Questionnaire–9 equivalency from baseline, (2) remained at a moderate level of depression for 2 or more weeks in a row, or (3) indicated severe symptoms or worsening moderate symptoms over 2 weeks. If participants did not schedule directly, CHA-MW coordinating staff trained in motivational interviewing reached out to participants by phone to set up an outreach triage appointment with a mental wellness counselor. The mental wellness counselors were postgraduate-level trainees in the institution who were completing hours of supervised practice toward full, independent licensure, including psychology PhD graduates completing supervised post–doctoral training or licensed clinical social workers doing supervised training hours. These counselors provided brief behavioral interventions as needed or referred participants to more specialized psychiatric care using a stratified care algorithm. Notably, mental wellness counselors were not allowed to use mindfulness interventions or refer participants in the study to any mindfulness-based therapy or mindfulness groups for the duration of the study period.

#### MBCT Skills for Resilience During COVID-19 (MBCT-R)

MBCT-R was a live, online, 8-week, 2.25-hour, and psychoeducational group program modestly adapted from MBCT [24] to address stressors specific to the COVID-19 pandemic as well as logistic challenges of the COVID-19 era, including high demand for service and the necessity of remote online delivery. Adaptations were developed through expert review of the MBCT literature by MPI (CF) paired with feedback from the CHA Patient Advisory Board, including 5 patient stakeholders. The key adaptations included (1) shifting the wording used throughout the intervention to focus on a broader variety of mental health symptoms, specifically including anxiety and pandemic-related distress, in addition to depression; (2) enabling delivery of the group via telehealth platform; and (3) allowing enrollment of up to 40 participants at a time with 2 MBCT-R group coleaders. This manual-based intervention had a standard curriculum and flow (Table 1) that consisted of training in formal and informal mindfulness practices intended to increase participants’ awareness of the relationship between thoughts, feelings, and body sensations in daily life. Groups included discussion of practices and assignment of home practice. Supporting materials were provided via secure web portal and included readings, detailed instructions for home practice, and prerecorded audio guided practices. In addition to the 8 weekly sessions, MBCT-R also included a half-day, 4-hour online retreat generally between weeks 6 and 7. Participants were considered to have completed MBCT-R if they attended at least 6 sessions during the 8-week program.

**Table 1.** MBCT-R^a^ and iCBT^b^ session outlines.

	MBCT-R		iCBT: MoodGYM	
Week	Title and themes	Content	Title	Content
1	Awareness & Automatic Pilot Mind-wandering Habituated behaviors Present moment awareness	Raisin exercise Body scan Mindful inquiry Short breath focus	Introduction	Preprogram measures Introduction of core CBT^c^ concepts using 6 fictional characters
2	Living in Our Heads The wandering mind Obstacles to paying attention The breath as an anchor	Body scan Inquiry and practice review Thoughts and feelings exercise Brief sitting meditation (breath, body)	Feelings	Negative thought patterns Biased perceptions Negative views about the self and future The link between thoughts and feelings
3	Gathering the Scattered Mind Lost in thought Welcoming and curious attitude Bringing mindfulness to daily life	Sitting (breath, body, sensations) Mindful movement 3-minute breathing space Inquiry and practice review	Thoughts	Identifying and challenging biased or “warpy” thoughts Identifying vulnerabilities Self-esteem Pleasant activity scheduling
4	Recognizing Aversion Avoidance and attachment Turning toward difficulty A different place to view thoughts	Sitting (breath, body, sounds, thoughts) Automatic Thoughts Questionnaire 3-minute breathing space responsive Mindful walking Inquiry and practice review	Unwarping	Reporting on thoughts in the 3rd person Thought challenging Increasing social and physical activity Testing new ways of responding
5	Allowing and Letting Be A different relationship to the unwanted Acceptance; Window of tolerance Moving toward self-care	Sitting meditation focus on difficulty Inquiry and practice review Working with difficulty Breathing space	Destressing	Stress and identifying stressors “Relax Fest” game show—a quiz about relaxation Relaxation techniques
6	Thoughts are Not Facts How moods influence thoughts Thoughts as mental events Early warning signs of emotional distress	Sitting meditation focus on thoughts Moods and thoughts Alternative viewpoints Discuss relapse signature Discuss retreat	Relationships	The link between thoughts, emotions, and relationships Problem-solving skills
6.5 Half- Day Retreat	Making the Practice Your Own Being in silence Going deeper with mindfulness Strengthening the practice	Mindful walking Loving kindness meditation Discussion of practices Discuss preparation for end of course	N/A^d^	N/A^d^
7	How Can I Best Take Care of Myself? Behavior and mood are interrelated Skills to prevent relapse Fostering resilience	Sitting meditation (all) Exploring links between activity and mood Nourishing and depleting exercise links between activity and mood Scheduling activities and mindful action Action plan for staying well	Review	Postprogram measures and comparison with earlier results “Wrapping it Up” review and summary Certificate of completion
8	Maintaining and Extending New Learning Ending and beginnings The importance of self-care Well-being and resilience What is most important to us	Body scan Review action plans Review of entire course Keeping practice going Closing exercise	N/A^d^	N/A^d^

a MBCT-R: Mindfulness-Based Cognitive Therapy for Resilience.

b iCBT: internet Cognitive Behavioral Therapy.

c CBT: cognitive behavioral therapy.

d N/A: not applicable.

#### iCBT: MoodGYM

Generally, iCBT describes a family of evidence-based, online programs that target depression, anxiety, and stress to improve general psychological well-being [56]. In particular, this study used MoodGYM, a widely implemented and researched iCBT program developed by the Centre for Mental Health Research at the Australian National University [57] that integrated principles and techniques of cognitive behavioral therapy (CBT) and interpersonal therapy. Research demonstrated MoodGYM to be effective in reducing symptoms of depression, general psychological distress, and suicidal ideation, as well as improving indicators of quality of life [58-60]. Additionally, a 2016 meta-analysis of 12 studies found MoodGYM to be effective at reducing depression and anxiety symptoms among adult populations, with further evidence suggesting that it is also effective at reducing general psychological distress [61]. MoodGYM included six, 1-hour sessions comprising 5 curriculum modules and a review session. As an alternative to MBCT-R, this web-based program was well suited to offer an on-demand, asynchronous, and individual format with 6 sessions to complete in an 8-week period similar to MBCT-R (Table 1).

MoodGYM was considered a suitable active comparator for MBCT-R, as an evidence- and CBT-based, transdiagnostic treatment delivered via remote platforms, which also provided meaningful dimensionality in terms of the method of delivery (eg, group synchronous vs individual asynchronous) and intensity of the intervention (2.25 vs 1 hour per week). Completion of the program was self-paced, with the exception that participants were encouraged to wait at least 5 days between consecutive lessons and complete all lessons within 8 weeks. Participants were considered to have completed iCBT if they participated in all 6 sessions over the 8-week program.

#### Measures

**Figure 1.**
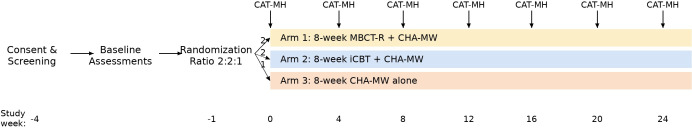
Study allocation and assessment schedule. CAT-MH: Computer Adaptive Testing for Mental Health; CHA-MW: Cambridge Health Alliance MindWell; iCBT: internet Cognitive Behavioral Therapy; MBCT-R: Mindfulness-Based Cognitive Therapy for Resilience.

Participants were followed for 24 weeks from the start of the intervention with not only recurrent monthly online CAT-MH assessments for depression and anxiety (Figure 1) but also monthly assessments for exploratory measures of perceived stress, interoceptive awareness, emotion dysregulation, experiential avoidance, self-compassion, and decentering.

All study surveys (prescreening, screening, baseline, and study assessments) were deployed and hosted via REDCap [53], including the CAT-MH, which was completed via a customized portal using the CAT-MH API integrated into a REDCap external module [55]. Participants’ demographic characteristics were collected at baseline via telephone interviews and self-report surveys. Full CAT-MH assessments were completed at baseline and weeks 4, 8, 12, 16, 20, and 24.

#### Primary Outcome: Depression

The study’s primary outcome was change in depression symptom severity between baseline and week 24 postrandomization, with MBCT-R+CHA-MW being the primary intervention of interest as compared with CHA-MW alone. Depression was assessed using the CAT-MH (CAT-DEP), a dimensional measure that enables precise, timely individual assessment and monitoring while also facilitating group-level comparative analyses based on aggregated data [49,51]. Based on multidimensional item response theory, CAT-DEP adaptively administers 12 questions from an item bank of 389 possible questions to estimate symptom severity while maintaining a 0.95 correlation with the total item bank score [49]. Scores are standardized as T-scores that represent the individual’s performance relative to a reference group, with a range of 100, a mean of 50, and an SD of 10. T-scores are calculated by converting a raw score to a z-score and then using the formula T=10 z+50. For CAT-DEP, scores are grouped as normal (less than 50), mild symptoms (50-65), moderate symptoms (66-75), and severe symptoms (higher than 75) [49]. Research has found the CAT-DEP to correlate well with gold standard measures of depression symptom severity, including the Center for Epidemiologic Studies Depression Scale [62] (*r*=0.90), the Hamilton Rating Scale for Depression–25 [63] (*r*=0.79), and the Patient Health Questionnaire–9 [64] (*r*=0.90).

#### Secondary Outcome: Mental Health Clinician Visit Use

The study’s secondary outcome was the number of mental health clinician visits used during the 24-week study period after randomization. Visit data were extracted from participants’ EPIC [65] electronic health record for the 24-week study period and included 4 types: telephone encounters (ie, CHA-MW wellness check-ins), individual psychotherapy, group visits, and individual psychopharmacology. Group visits were defined as any behavioral health-oriented visit conducted in a group setting (eg, smoking cessation group, pain management group, or group psychotherapy). Psychopharmacology visit data within 24 weeks prior to randomization were collected.

#### Exploratory Outcomes

Exploratory mental health outcomes included changes in anxiety symptom severity and perceived stress scores between baseline and week 24, as well as changes in several potential mechanistic factors including interoceptive awareness, experiential avoidance, emotional dysregulation, self-compassion, and decentering scores at baseline and during the 24 weeks after randomization.

Anxiety was measured by CAT-ANX [66], which adaptively administers 12 questions from an item bank of 437 possible questions to estimate anxiety symptom severity. Similar to the CAT-DEP, scores are standardized as T-scores, with a range of 100, a mean of 50, and an SD of 10, which represent the individual’s performance relative to a reference group. For CAT-ANX, scores are grouped as normal (<35), mild symptoms (35-50), moderate symptoms (51-65), and severe symptoms (>65) [66].

Perceived stress was assessed by the 14-item Perceived Stress Scale with items scored from 0 (Never) to 4 (Very Often) (Cronbach α=0.84‐0.86) [67]. The 14-item Perceived Stress Scale is designed to measure the degree to which an individual appraises situations in their life as unpredictable, uncontrollable, and overloading, with higher scores indicating greater perceived stress.

Interoceptive awareness was measured by the 37-item Multidimensional Assessment of Interoceptive Awareness–2 [68], with items scored from 0 (never) to 5 (always) (Cronbach α=0.64‐0.83) [68]. The Multidimensional Assessment of Interoceptive Awareness–2 measures an individual’s awareness of their internal bodily states, such as breathing or pain. It includes 8 scales measuring different dimensions of interoceptive awareness, including Noticing, Not distracting, Not worrying, Attention regulation, Emotional awareness, Self-regulation, Body listening, and Trusting, with higher scores on these subscales indicating greater interoceptive awareness.

Emotion regulation capability was assessed by the 36-item Difficulties in Emotion Regulation Scale [69], with items scored from 1 (Almost Never, 0%‐10%) to 5 (Almost Always, 91%‐100%) (Cronbach α=0.94) [70]. The Difficulties in Emotion Regulation Scale assesses an individual’s ability to understand, accept, and manage emotions effectively, with higher scores indicating greater difficulties with emotion regulation.

Experiential avoidance was assessed by the 15‐item Brief Experiential Avoidance Questionnaire, with items scored from 1 (Strongly Disagree) to 6 (Strongly Agree) (Cronbach α=0.80‐0.83) [71]. The Brief Experiential Avoidance Questionnaire measures an individual’s tendency to avoid or escape from unwanted internal experiences, such as uncomfortable thoughts, emotions, or sensations, with higher scores indicating higher levels of experiential avoidance.

Self‐compassion was assessed using the 12-item Self‐Compassion Scale–Short Form [72], with items scored from 1 (Almost Never) to 5 (Almost Always) (Cronbach α=0.87) [72]. The Self‐Compassion Scale–Short Form measures an individual’s capacity for self-compassion including aspects of self-kindness, self-judgment, common humanity, isolation, mindfulness, and overidentification, with higher scores indicating higher levels of self-compassion.

Decentering was measured using the 11-item Decentering subscale from the Experiences Questionnaire [73], with items scored from 0 (Never) to 4 (All the time) (Cronbach α=0.83) [73]. The Decentering subscale from the Experiences Questionnaire assesses an individual’s ability to take a detached view of their thoughts and feelings rather than identifying with them, taking a nonjudgmental and accepting stance to temporary mental events, with higher scores indicating higher levels of decentering.

### Adverse Events

Staff monitored adverse events at each study visit and through REDCap surveys administered at weeks 8, 16, and 24. Events were assessed for severity, relatedness, and expectedness, and were regularly reviewed by a data safety and monitoring board approved by the NCCIH.

### Analysis

Randomization was assessed by comparing the baseline characteristics of participants assigned to all 3 intervention arms using *t* tests, chi-square tests, or Fisher exact tests. For the primary outcome regarding depression, we conducted an intention-to-treat, repeated-measures analysis using generalized linear mixed-effects models (mixed) with a week by study group interaction term to estimate differences between MBCT-R+CHA-MW vs CHA-MW in depression severity (CAT-DEP) from baseline to 24-week follow-up. As a secondary outcome, we also examined the change in depression from baseline to week 24 comparing iCBT+CHA-MW with CHA-MW.

For the completer analysis, using the a priori per protocol completer definition (completion of at least 6 sessions of either MBCT-R or iCBT), we estimated the difference between participants who did and did not complete their allocated intervention within the MBCT-R+CHA-MW and iCBT+CHA-MW arms over time by adding a 3-way interaction term representing study week, group, and completer status.

For the secondary outcome of mental health clinician visit utilization during the 24-week study period, we used logistic regression to compare the proportion of participants with at least 1 mental health clinician visit across study groups. We also conducted post hoc moderation analyses to investigate potential impacts of differential visit usage on both primary and exploratory mental health outcomes.

Finally, 7 exploratory mediators of change were analyzed, chosen based on published studies of MBCT, CBT, and other related interventions, including anxiety, perceived stress, interoceptive awareness, experiential avoidance, emotional dysregulation, self-compassion, and decentering. We conducted a difference-in-differences intention-to-treat repeated-measures analysis using linear mixed-effects models with a study week by group interaction term to estimate the relative changes from baseline to week 24.

Assuming a 2-sided significance level α=.05, we calculated using Stata (version 16; StataCorp LLC) [74] that a sample of 200 participants randomized 2:2:1 would have provided 80% power to detect an effect size of *d*=−0.54 for between-group differences on the primary aim comparison of changes in depression severity (CAT-DEP) when comparing the MBCT-R+CHA-MW vs CHA-MW arms. Maximum likelihood estimation (MLE) was used to address missingness for all analyses. We also conducted multiple imputation for a sensitivity analysis, with comparable results, and so the former are reported here. We planned to adjust the models to account for baseline covariates that differed between study groups after randomization (*P*<.10). We accounted for multiple comparisons using the Benjamini-Hochberg false discovery rate procedure [75]. Effect sizes (Cohen *d*) were calculated. For the number of adverse events, we conducted a negative binomial regression to evaluate between-group differences. All analyses were conducted in Stata (version 18; StataCorp LLC) [76].

## Results

### Participant Flow and Demographics

**Figure 2.**
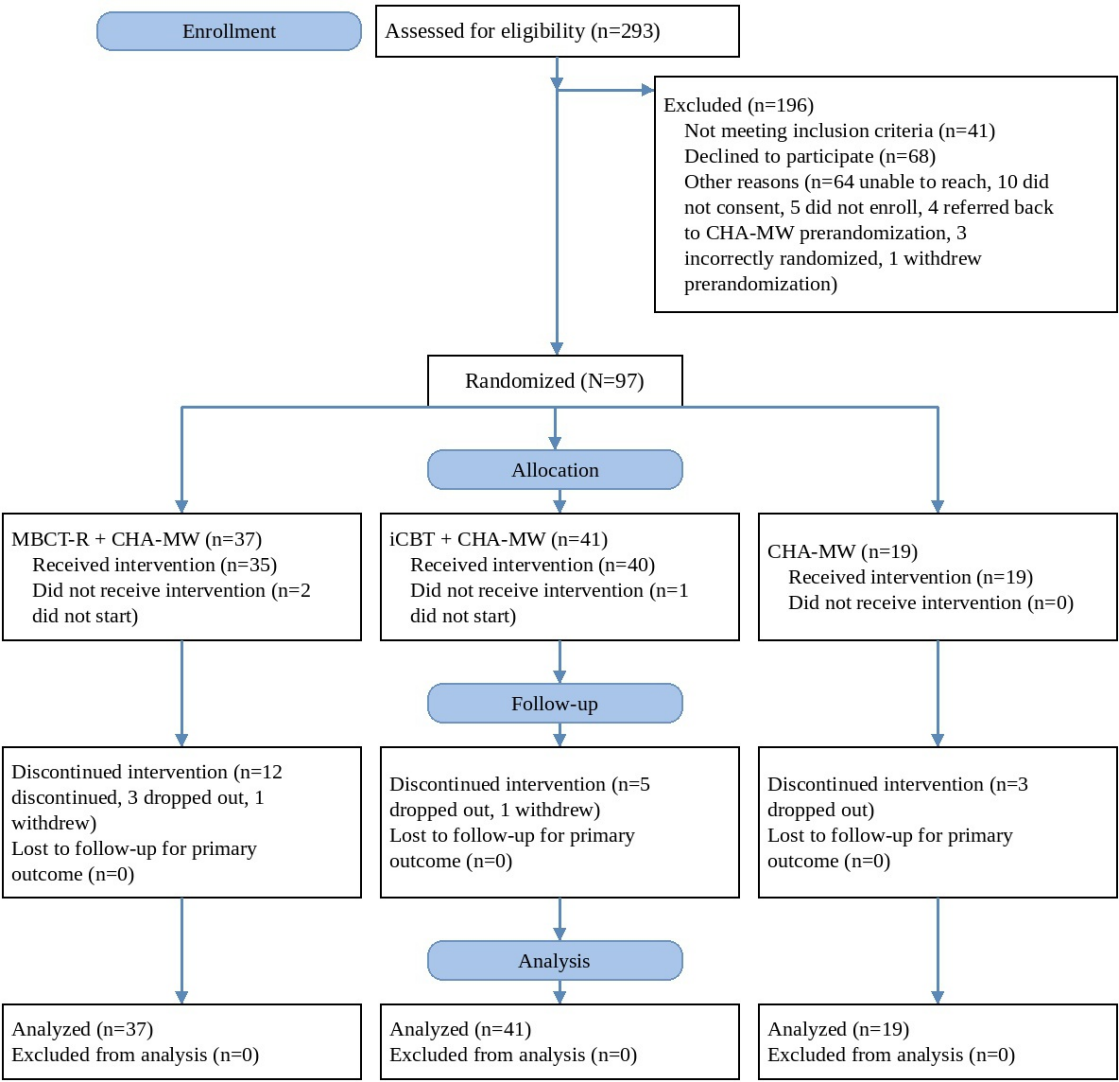
CONSORT (Consolidated Standards of Reporting Trials) flow diagram. CHA-MW: Cambridge Health Alliance MindWell; iCBT: internet Cognitive Behavioral Therapy; MBCT-R: Mindfulness-Based Cognitive Therapy for Resilience.

From March 19, 2021, to September 2, 2022, a total of 293 people were referred to the study, 113 completed informed consent and enrolled, and 97 were randomized to 1 of the 3 study arms (MBCT-R+CHA-MW: n=37; iCBT+CHA-MW: n=41; and CHA-MW: n=19) (Figure 2). Randomized participants (N=97) were 73% (71/97) female and, on average, aged 40.0 (SD 11.9) years, with 76% (74/97) identifying as White and 28% (27/97) identifying as American Indian or Alaska Native, Asian, Black, Haitian, or African American, or Other. Participants could endorse more than 1 demographic for race. No significant differences in baseline demographics were found between the 3 study arms (Table 2). Assessment completion between study arms was similar in all 3 arms with no difference between groups in the rates of participants who completed all 4 main assessment sessions: 67% (25/37) of the MBCT-R+CHA-MW arm, 68% (28/41) of the iCBT+CHA-MW arm, and 73% (14/19) of the CHA-MW arm.

**Table 2.** Participant baseline demographics by study arm.

Demographic or characteristic	CHA-MW^a^ (n=19)	MBCT-R^b^ (n=37)	iCBT^c^ (n=41)	Total (N=97)
Sex, n (%)				
Female	12 (63.2)	26 (70.3)	33 (80.5)	71 (73.2)
Male	7 (36.8)	11 (29.7)	8 (19.5)	26 (26.8)
Age (years), mean (SD)	35.4 (9.0)	40.6 (12.8)	41.7 (12.0)	40.0 (11.9)
Race, n (%)				
American Indian or Alaska Native	1 (5.2)	0 (0)	0 (0)	1 (1.0)
Asian	3 (15.8)	3 (8.1)	4 (9.8)	10 (10.3)
Black, Haitian, or African American	2 (10.5)	1 (2.7)	3 (7.3)	6 (6.2)
Other	2 (10.5)	3 (8.1)	5 (12.2.)	10 (10.3)
White	14 (73.7)	29 (78.4)	31 (75.6)	74 (76.3)
Ethnicity, n (%)				
Hispanic, Spanish, or Latinx	3 (15.8)	8 (21.6)	9 (22.0)	20 (20.6)
English as a second language, n (%)	3 (15.8)	5 (13.5)	7 (17.1)	15 (15.5)
Relationship status, n (%)				
Single	10 (52.6)	23 (62.2)	18 (43.9)	51 (52.6)
Married or living as married	8 (42.1)	11 (29.7)	21 (51.2)	40 (41.2)
Education (years), mean (SD)	17.6 (2.8)	16.5 (3.1)	16.7 (3.5)	16.8 (3.2)
Sexual identity, n (%)				
Straight or heterosexual	14 (73.7)	23 (62.2)	25 (61.0)	62 (63.9)
Lesbian, gay, or homosexual	1 (5.3)	4 (10.8)	3 (7.3)	8 (8.2)
Bisexual	1 (5.3)	7 (18.9)	8 (19.5)	16 (16.5)
Other	2 (10.5)	2 (5.4)	1 (2.4)	5 (5.2)
Don’t know	0 (0)	0 (0)	3 (7.3)	3 (3.1)
Prefer not to say	0 (0)	0 (0)	1 (2.4)	1 (1.0)

a CHA-MW: Cambridge Health Alliance MindWell.

b MBCT-R: Mindfulness-Based Cognitive Therapy for Resilience.

c iCBT: internet Cognitive Behavioral Therapy.

Baseline differences in primary and secondary outcome variables necessitated running primary and secondary analyses with baseline values as covariates. For the primary analysis (1^o^: MBCT-R+CHA-MW vs CHA-MW), baseline values of decentering (secondary outcome) were significantly lower in the CHA-MW arm than in the MBCT-R+CHA-MW arm (*P*=.04) (Table 3), so subsequent analyses were run with baseline decentering values as a covariate. For the secondary analysis (2^o^: iCBT +CHA-MW vs CHA-MW), baseline values of both decentering and depression were lower in the iCBT +CHA-MW arm than in the CHA-MW arm (*P*=.02, *P*=.04). Therefore, these secondary analyses were run with both baseline depression and baseline decentering values as covariates alongside baseline levels of the measured outcome.

**Table 3.** Participant baseline primary and exploratory characteristics by study arm.

	CHA-MW^a^ (n=19)	MBCT-R^b^ (n=37)	iCBT^c^ (n=41)	Total (n=97)
Primary outcome, mean (SD)				
Depression (CAT-DEP^d^)	59.6 (10.7)	57.6 (11.1)	51.4 (12.3)	55.3 (11.9)
Exploratory outcomes, mean (SD)				
Anxiety (CAT-ANX^e^)	46 (14.0)	45.5 (13.6)	41.1 (18.4)	43.7 (15.9)
Perceived Stress (PSS-14^f^)	39.8 (7.0)	40.4 (5.9)	37.1 (7.4)	38.9 (6.9)
Interoceptive awareness (MAIA-2^g^)	2.2 (0.6)	2.3 (0.7)	2.5 (0.7)	2.4 (0.7)
Emotion Dysregulation (DERS^h^)	103.9 (22.3)	94.6 (18.8)	95.2 (23.6)	96.7 (21.7)
Experiential Avoidance (BEAQ^i^)	52.7 (11.4)	50.7 (9.1)	51.2 (13.5)	51.3 (11.5)
Self-Compassion (SCS-12^j^)	2.4 (0.7)	2.7 (0.7)	2.5 (0.8)	2.5 (0.7)
Decentering (EQ-D^k^)	29.8 (4.0)	33.0 (5.7)	32.5 (5.7)	32.2 (5.5)

a CHA-MW: Cambridge Health Alliance MindWell.

b MBCT-R: Mindfulness-Based Cognitive Therapy for Resilience.

c iCBT: internet Cognitive Behavioral Therapy.

d CAT-DEP: Computer Adaptive Testing for Mental Health Depression module; scores were obtained for 18 participants at baseline for the CHA-MW arm.

e CAT-ANX: Computer Adaptive Testing for Mental Health Anxiety module; scores were obtained for 18 participants at baseline for the CHA-MW group.

f PSS-14: 14-item Perceived Stress Scale.

g MAIA-2: Multidimensional Assessment of Interoceptive Awareness–2.

h DERS: Difficulties in Emotion Regulation Scale.

i BEAQ: Brief Experiential Avoidance Questionnaire.

j SCS-SF: Self-Compassion Scale–Short Form.

k EQ-D: Decentering subscale from the Experiences Questionnaire.

Interim power analyses indicated that for an anticipated sample size of 100 randomized participants (40:40:20) the study had 80% power to detect a difference of at least *d*=0.72 between MBCT-R+CHA-MW (n=40) and CHA-MW (n=20) and 80% power to detect a difference of at least *d*=0.63 between MBCT-R+CHA-MW (n=40) and iCBT (n=40).

### Outcomes

#### Primary Outcome: Depression

For the primary outcome, the mean depression (CAT-DEP) score significantly decreased in all groups from baseline to week 24 (Table 4).

**Table 4.** Depression scores by study arm and week for primary (1^o^) and secondary (2^o^) outcome analyses.

Week	1^o^: MBCT-R^a^, mean (SD)	n	2^o^: iCBT^b^, mean (SD)	n	CHA-MW^c^, mean (SD)	n	Total , mean (SD)	n
0	57.6 (11.1)	37	51.4 (12.3)	41	59.6 (10.7)	18	55.3 (11.9)	96
4	46.7 (17.4)	28	39.8 (14.4)	35	48.4 (11.7)	15	44 (15.4)	78
8	41.3 (18.1)	30	38.3 (15.5)	31	44.5 (11.6)	15	40.7 (15.9)	76
12	45.8 (17.9)	28	39.7 (16.1)	28	42.5 (14)	14	42.7 (16.5)	70
16	41 (18.4)	23	41.2 (19.7)	29	44.7 (13.8)	15	41.9 (17.9)	67
20	44.8 (21.2)	20	40.5 (19.1)	27	46.2 (14.5)	14	43.2 (18.7)	61
24	43.2 (19.9)	23	37.4 (17.1)	29	42.8 (16.7)	15	40.6 (18)	67

a MBCT-R: Mindfulness-Based Cognitive Therapy for Resilience.

b iCBT: internet Cognitive Behavioral Therapy.

c CHA-MW: Cambridge Health Alliance MindWell.

As reflected in Table 5, findings indicated a statistically significant decrease of 15.2 points in the CHA-MW control group (95% CI −21.8 to −8.6; *P*<.001), 14.1 points in the MBCT-R+CHA-MW arm (95% CI −21 to −7.2; *P*<.001), and 12.7 points in the iCBT+CHA-MW arm (95% CI −17.4 to −8.1; *P*<.001). However, there were no significant between-group differences in depression reduction between MBCT-R+CHA-MW and CHA-MW over 24 weeks in the primary analysis (95% CI −8.5 to 10.7; *P*=.82), and no between-group difference between iCBT+CHA-MW and CHA-MW in the secondary analysis (95% CI −5.6 to 10.6; *P*<.55). Further exploratory analysis comparing all 3 study arms over time found no significant between-group differences (*χ*²_12_=12.3; *P*=.42) (Table S2 in Multimedia Appendix 1).

**Table 5.** Differences in primary (1^o^), secondary (2^o^), and exploratory outcome analyses.

Group	Within group (pre-post)				Between group (pre-post relative to control)			
Outcome	Pre: mean (SD)	Difference	95% CI	*P* Value	Difference	95% CI	Effect size (*d*)	*P* value
Depression								
CHA-MW^a^	59.6 (10.7)	−15.2	−21.8 to −8.6	<.001	Ref^b^	Ref	Ref	Ref
1^o^: MBCT-R^c^	57.6 (11.1)	−14.1	−21 to −7.2	<.001	1.1	−8.5 to 10.7	0.1	.82
2^o^: iCBT^d^	51.4 (12.3)	−12.7	−17.4 to −8.1	<.001	2.5	−5.6 to 10.6	0.2	.55
Anxiety								
CHA-MW	46.0 (14.0)	−15.3	−22.5 to −8	<.001	Ref	Ref	Ref	Ref
MBCT-R	45.5 (13.6)	−15.2	−21.3 to −9.1	<.001	0.1	−9.4 to 9.5	0.00	.99
iCBT	41.1 (18.4)	−12.1	−16.3 to −8	<.001	3.1	−5.2 to 11.4	0.24	.46
Perceived stress								
CHA-MW	39.8 (7.0)	−6.0	−9.6 to −2.5	<.001	Ref	Ref	Ref	Ref
MBCT-R	40.4 (5.9)	−6.1	−9.2 to −3	<.001	0.0	−4.7 to 4.7	0.0	.99
iCBT	37.1 (7.4)	−4.8	−7.4 to −2.1	<.001	1.3	−3.1 to 5.7	0.2	.57
Interoceptive awareness								
CHA-MW	2.2 (0.6)	0.3	0 to 0.5	.03	Ref	Ref	Ref	Ref
MBCT-R	2.3 (0.7)	0.6	0.2 to 1	.005	0.3	−0.2 to 0.8	0.7	.18
iCBT	2.5 (0.7)	0.2	0 to 0.4	.02	0.0	−0.3 to 0.3	−0.1	.75
Experiential avoidance								
CHA-MW	52.7 (11.4)	−3.1	−5.3 to −0.8	.008	Ref	Ref	Ref	Ref
MBCT-R	50.7 (9.1)	−5.5	−10.6 to −0.4	.04	−2.4	−8.1 to 3.2	−0.4	.39
iCBT	51.2 (13.5)	−3.5	−5.8 to −1.1	.004	−0.4	−3.6 to 2.8	−0.1	.80
Emotion dysregulation								
CHA-MW	103.9 (22.3)	−14.4	−22.5 to −6.2	<.001	Ref	Ref	Ref	Ref
MBCT-R	94.6 (18.8)	−11.0	−22 to −0.1	.048	3.3	−10.3 to 16.9	0.3	.63
iCBT	95.2 (23.6)	−8.9	−15.3 to −2.5	.006	5.5	−4.8 to 15.8	0.4	.30
Self-compassion								
CHA-MW	2.4 (0.7)	0.4	0.1 to 0.6	.001	Ref	Ref	Ref	Ref
MBCT-R	2.7 (0.7)	0.5	0.1 to 0.9	.02	0.1	−0.4 to 0.6	0.3	.62
iCBT	2.5 (0.8)	0.3	0.1 to 0.5	<.001	−0.1	−0.4 to 0.2	−0.2	.60
Decentering								
CHA-MW	29.8 (4.0)	3.6	1.4 to 5.7	.001	Ref	Ref	Ref	Ref
MBCT-R	33.0 (5.7)	2.7	−0.4 to 5.8	.09	−0.9	−4.7 to 2.9	−0.2	.64
iCBT	32.5 (5.7)	1.1	−0.4 to 2.5	.16	−2.5	−5.1 to 0.1	−0.7	.06

a CHA-MW: Cambridge Health Alliance MindWell.

b Ref: reference value.

c MBCT-R: Mindfulness-Based Cognitive Therapy for Resilience; 1o: analysis for primary intervention of interest.

d iCBT: internet Cognitive Behavioral Therapy; 2o: analysis for secondary intervention of interest.

**Figure 3.**
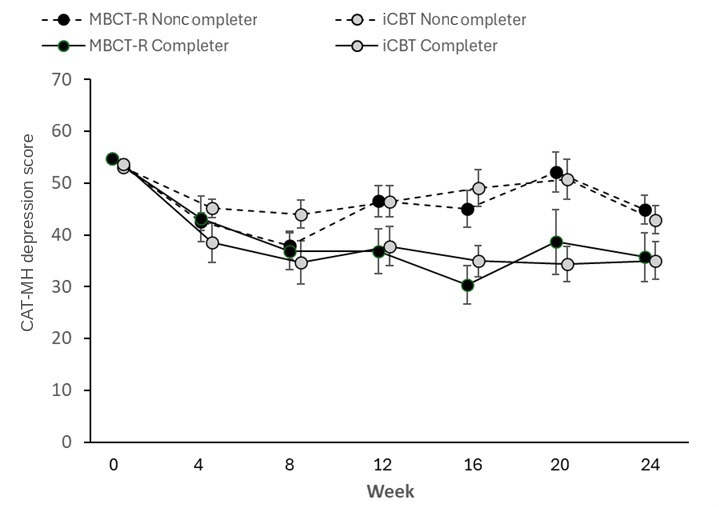
Marginal estimates of CAT-MH depression score by study week, group, and completer status. The figure shows the mean and standard error of the marginal estimate by study week, group, and completer status.CAT-MH: Computer Adaptive Testing for Mental Health; iCBT: internet Cognitive Behavioral Therapy; MBCT-R: Mindfulness-Based Cognitive Therapy for Resilience.

In the completer sensitivity analysis, intervention completion rates (6+ sessions completed) for the group, synchronous MBCT-R intervention (11/37, 30%), were not significantly different than for the individual, asynchronous iCBT intervention (10/41, 24%). Figure 3 shows the marginal estimates from the mixed-effects model with a 3-way interaction term representing study week, group, and completer status. Completers, relative to noncompleters, showed a greater reduction in CAT-DEP by week 24 of −8.5 (95% CI −16.2 to −0.8; *P*=.03), with no significant difference in this reduction between study intervention arms (*P*=.93). Relative to the CHA-MW arm, the completers in the MBCT-R+CHA-MW and iCBT+CHA-MW arms had a nonsignificant additional decrease in CAT-DEP of −3.6 (95% CI −12.5 to 5.2; *P*=.42), and noncompleters had a nonsignificant relative increase of 4.5 (95% CI −4.0 to 13.0; *P*=.30) (Figure S1 in Multimedia Appendix 1).

#### Secondary Outcome: Mental Health Clinician Visit Use

Mental health clinician visits were analyzed both in the aggregate and by visit type, including telephone encounters (ie, CHA-MW wellness check-in), individual psychotherapy, individual psychopharmacology, and group psychotherapy visits. Results regarding aggregated visits indicated no significant between-group differences in mental health clinician visit use (Table 6 and Table S3 in Multimedia Appendix 1) for nonparametric descriptive statistics. On average, there was no significant difference in participants’ overall health visit usage across the study arms.

**Table 6.** Mental health clinician visit use by study arm.

	CHA-MW^a^ (n=19)	MBCT-R^b^ (n=37)	iCBT^c^ (n=41)	Total (n=97)
Total visits during 24 weeks, mean (SD)	2.53 (4.26)	3.62 (5.23)	1.73 (3.07)	2.61 (4.28)
Telephone visits	1.05 (1.18)	1.14 (1.77)	0.68 (0.99)	0.93 (1.37)
Psychotherapy	0.95 (3.22)	1.51 (3.84)	0.46 (1.67)	0.96 (2.98)
Psychopharmacology	0.53 (1.31)	0.95 (1.88)^d^	0.05 (0.31)	0.48 (1.36)
Group	0 (0)	0.03 (0.16)	0.54 (1.86)	0.24 (1.23)

a CHA-MW: Cambridge Health Alliance MindWell.

b MBCT-R: Mindfulness-Based Cognitive Therapy for Resilience.

c iCBT: internet Cognitive Behavioral Therapy.

d *P*=.005.

When visits were disaggregated by type, exploratory findings revealed that patients in the MBCT-R+CHA-MW arm were more likely than those in the iCBT+CHA-MW arm to be engaged in psychopharmacology visits during the intervention (difference=0.92, 95% CI 0.28-1.55; *P*=.005). However, although baseline levels of individual psychopharmacology visits during the 24 weeks prior to the intervention did not statistically differ among the arms (Fisher exact *P*=.24), there was some numerical variation. Descriptively, the presence of any individual psychopharmacology treatment visit increased in the CHA-MW arm from 0% (0/19) having a psychopharmacology treatment visit during the 24 weeks prior to study randomization to 20% (4/19) of the sample during the 24 weeks following randomization. Similarly, psychopharmacology visits numerically increased in the MBCT-R+CHA-MW arm from 14% (5/37) of participants during the 24 weeks prior to randomization to 24% (9/37) of participants during the 24 weeks following randomization, whereas psychopharmacology visits decreased from 7% (3/41) of participants to 2% (1/41) in the iCBT +MW arm (Table S4 in Multimedia Appendix 1). A sensitivity moderation analysis confirmed that the number of mental health clinician visits during the intervention did not impact the primary or exploratory mental health results between groups (Table S5 in Multimedia Appendix 1).

#### Exploratory Outcomes

All 3 study arms demonstrated significant within-group pre-post decreases for anxiety, perceived stress, experiential avoidance, and emotional dysregulation, in addition to increases in interoceptive awareness and self-compassion. Additionally, for each of these mechanistic outcomes, no significant between-group differences (MBCT-R+CHA-MW vs CHA-MW, or iCBT+CHA-MW vs CHA-MW) were found in the magnitude of pre-post change or in the rate of change over the 24-week study period. Decentering differed at baseline for the CHA-MW arm, limiting interpretability of that exploratory analysis (Table 5 and Table S6 in Multimedia Appendix 1).

### Adverse Events

Twenty adverse event reports (AERs) were completed during the study (10/37, 27% [MBCT-R] vs 10/41, 24% [iCBT] vs 0/19, 0% [CHA-MW]; *P*=.045). Analyses indicated significantly fewer AERs in the CHA-MW arm than in both the MBCT-R+CHA-MW and iCBT +CHA-MW arms. One possible explanation for this difference is that participants in the CHA-MW arm had fewer contact points with the study team, during which they might have volunteered or been asked about potentially adverse events. Of the AERs reported, 18 were not serious, with 2 being related to the protocol. One MBCT-R participant experienced back pain–related anxiety during meditation and elected to discontinue the intervention. One iCBT participant reported feeling “worse” when completing the online modules and elected to discontinue the intervention. Regarding the 2 serious adverse events reported, neither were related to the protocol. Two MBCT-R participants were hospitalized for medical concerns, a blood clot and a kidney concern, respectively; however, both elected to continue the intervention.

## Discussion

### Main Findings

Contrary to study hypotheses, findings from this randomized controlled clinical trial did not demonstrate that MBCT-R+CHA-MW was more effective than CHA-MW alone in reducing depressive symptoms. Therefore, we did not find support for an additive effect of adding MBCT-R to CHA-MW. Furthermore, the magnitude of participants’ overall engagement with mental health clinician visits beyond their assigned intervention did not differ between groups, suggesting that study assignment did not impact overall mental health service clinical visit utilization significantly during the 24-week study period.

Low levels of intervention completion and study attrition during the COVID-19 pandemic may have limited the study’s ability to detect between-group differences. Most importantly, intervention arm participants had low rates of intervention completion (6+ sessions) in the completer analysis both for MBCT-R (30%) and for iCBT (24%), with an approximately 8-point difference in CAT-DEP score reduction between evidence-based intervention completers and noncompleters. This is a potential contributing factor for lack of superiority between either of the 2 additive evidence-based intervention arms versus the CHA-MW arm. The rate of completion was lower than expected for MBCT-R. As the live online, synchronous, and group-based option providing social connection, the MBCT-R+CHA-MW arm was anticipated to have a higher level of engagement with lower attrition rates than what occurred in the study [77]. While videoconference delivery of MBCT-R was anticipated to have greater attrition than what is typically seen in-person MBCT delivery (19%‐26%), MBCT-R was still expected only to have a noncompletion rate similar to the 50% seen for other digital mindfulness-based group interventions [77,78] and not the 70% noncompletion rate found in this study. Notably, in comparison, the 24% completion rate for MoodGYM was not dissimilar to published trials for iCBT [58]. Given the significant difference in depression reduction between completers and noncompleters, a larger future study with higher evidence-based intervention completion rates may have the potential to demonstrate an additive effect that was not seen in this study. Notably, during the COVID-19 pandemic, digital and mental health programs experienced high rates of dropout and nonadherence, which was due to systemic and technological barriers [79,80], personal (eg, work or time conflicts, infection with COVID-19, etc) and psychological constraints (eg, pandemic distress, depression, and anxiety) [79,81], and baseline high attrition among diverse populations [82]. Both MBCT-R+CHA-MW and iCBT+CHA-MW were higher-intensity interventions than CHA-MW alone, which may have put greater demands on participant resources (eg, time willing to be online and difficulty having privacy to engage in the program), that were already strained for many people during the pandemic [83,84]. Finally, only approximately 69% (67/97) of participants completed all 4 CAT-DEP assessment timepoints, resulting in an acceptable, but not ideal, level of missing data that increased standard errors for estimating the primary outcome in both the intention-to-treat and completer analyses.

Several other additional crucial factors impacted the study’s ability to detect group differences. First, despite adequate randomization for the primary comparison, there were differing levels of depression symptom severity at baseline in the secondary analysis for iCBT+CHA-MW versus CHA-MW. Although baseline depression was added as a covariate in the mixed-effects regression, inadequate randomization is still a limitation of the secondary outcome analysis. Second, floor and ceiling effects may have had an impact, especially for iCBT in the secondary analysis, since this arm had a lower level of depression severity at baseline. Third, these differences also created the potential for bidirectional interactions between higher symptom severity or longer duration of depressive symptoms and treatment-seeking behaviors [85,86]. Fourth, on average, the study sample endorsed relatively lower baseline depression scores (mean 55.3, SD 11.9) that fell within the mild-moderate range for symptom classification (CAT-DEP scores=50‐65). This limited range may have made it difficult to detect significant between-group differences with this sample size. Finally, referral to psychopharmacology was part of CHA-MW’s stratified care algorithm, so those with new or worsening CAT-MH symptoms were referred to medication management. Since the CHA-MW arm had the largest increase in new psychopharmacology visits during the 24-week study period, this could have contributed to its effect; the low-dose comparator CHA-MW may have had more new referrals than the other arms to psychopharmacology to start an antidepressant medication trial, which is another well-proven evidence-based treatment for depression, and may account for some of the benefit of the CHA-MW approach.

When considered as a whole, results suggest the resource efficiency and relative effectiveness of CHA-MW as a stand-alone population mental health intervention, which pairs a low-intensity online psychoeducation community newsletter with a platform for remotely delivered, automated mental wellness monitoring and a responsive semiautomated, stratified care algorithm for referring people with worsening mental health symptoms to standard telemental health care. CHA-MW could be reasonably effective with or without additional remotely delivered mindfulness or CBT modules, which may be especially true during times with high-stress events (eg, COVID-19) when intervention adherence for both live online, synchronous, group-based interventions and asynchronous, individual, digital interventions may be more challenging for participants to complete.

### Primary Outcome: Depression

For the primary outcome, results indicated significant reductions in depression across all treatment arms. All CAT-MH modules receive a T-score on a 100-point scale. Therefore, the posterior standard deviation (uncertainty) of the severity estimates for all CAT-MH modules (eg, CAT-DEP) is 5 points on a 100-point scale, so the clinically meaningful difference for a single person is 5 points. As such, a 5-point change is beyond what can be expected based on measurement error alone, and the within-group point reductions ranging from 12.7 to 15.2 across all interventions represented clinically meaningful reductions in depression symptoms. However, no significant between-group differences were found. MBCT-R+CHA-MW, iCBT+CHA-MW, and CHA-MW arms all demonstrated similar patterns of effect. This finding aligns with previous comparative research findings. MBCT is generally effective for reducing current depressive symptoms with comparable effect sizes as specific, active controls, such as CBT, depression education programs, or psychoeducation, at posttreatment and 6-month follow-up, but it is more effective than nonspecific controls, such as waitlist or treatment as usual, at posttreatment but not follow-up [36]. Consistent with this literature, this study found that MBCT-R+CHA-MW demonstrated similar effectiveness to iCBT+CHA-MW (the specific, active comparator); however, it also was similar in effectiveness to CHA-MW (the nonspecific, lower intensity control) at 24-week follow-up. While these findings ran contrary to study hypotheses, which predicted that MBCT-R+CHA-MW would be more effective than CHA-MW alone, it is notable that the nonspecific CHA-MW intervention had previously demonstrated a preliminary effect of moderate size on depression among people with clinically significant symptoms (*d*=−0.42) [21]. While this study was not designed as a noninferiority trial, present findings suggest a potential for comparative effectiveness of CHA-MW as a stand-alone intervention for depressive symptoms when compared with 2 more resource-intensive, active, and evidence-based treatments during stressful periods, such as the COVID-19 pandemic, when adherence barriers for completing additional interventions may be common.

Importantly, from a methodological perspective, the iCBT+CHA-MW versus CHA-MW comparison had a large difference in mean depression at baseline that was greater than the 5-point clinically meaningful difference. While analytic methods addressed this by including CAT-DEP baseline levels as a covariate in the secondary analysis, the difference at baseline in the iCBT arm limited the ability to draw firm conclusions from the secondary analysis.

### Secondary Outcome: Mental Health Clinician Visit Use

Overall, no significant differences in overall mental health clinician visit use over the 24-week study period were found. Contrary to hypotheses, which predicted that mental health clinician visit use would decrease as intervention intensity increased, participants tended to have similar engagement with mental health clinician visits irrespective of their assigned intervention. This finding suggests that the interventions did not differentially impact visit use during the 24-week study period. Analysis of disaggregated visits (ie, telephone encounter, individual psychotherapy, individual psychopharmacology, and group psychotherapy) found a significant difference related to psychopharmacology visits, such that participants in the MBCT-R+CHA-MW arm were more likely to be engaged in psychopharmacology visits than those in the iCBT+CHA-MW arm, yet the majority of these participants in the MBCT-R+CHA-MW arm were already engaged with individual psychopharmacology visits prior to the study. Additionally, the largest increase in new psychopharmacology visits was in the CHA-MW arm (20% increase), which may have contributed to the depression reduction effect of CHA-MW, as previously noted. This may have happened in CHA-MW because the stratified care approach facilitated timely entrance to medication management for depression when appropriate. The brief 20-minute online video or phone visits, using a motivational interviewing approach, focused on providing a broad overview of mental wellness options available while also enhancing motivation for treatment and reducing stigma for psychopharmacologic options when appropriate. Notably, a subsequent sensitivity moderation analysis did not find that mental health visit use moderated primary or exploratory mental health outcomes. Regardless of their engagement with psychopharmacology visits, participants in both MBCT-R+CHA-MW and iCBT+CHA-MW arms demonstrated similar improvements in the measured mental health outcomes. This is consistent with past studies that found that preferences for medication did not reduce the effectiveness of MBCT [87].

Taken together, these findings suggest the relative usefulness of CHA-MW, particularly as it was the least demanding intervention of the 3 investigated in this study. However, it is also possible that intervention effects may not have translated into changes in mental health visit use during the 24-week study period. For instance, data for this study included only visits within our health care system; it is possible that participants sought psychotherapy elsewhere, or even that they may have been in therapy elsewhere and decided to stop due to improvement during the study. Follow-up studies in a larger sample with greater power, higher levels of intervention completion, and a longer follow-up period with collateral service data would help to render a clearer picture of how these interventions may differentially impact mental health visit use, which in turn would inform a greater understanding of their respective costs and benefits.

### Exploratory Outcomes

MBCT-R+CHA-MW did not demonstrate superiority compared with iCBT+CHA-MW or CHA-MW for reducing exploratory clinical outcomes of anxiety or stress. Significant within-arm improvements were found for 6 of the 7 exploratory mental health outcomes within each of the 3 study arms, including reduced anxiety, perceived stress, experiential avoidance, and emotional dysregulation, as well as increased interoceptive awareness and self-compassion. Additionally, for each of these mechanistic outcomes, no significant between-group differences were found in the magnitude of pre-post change or in the rate of change over the 24-week study period. MBCT-R+CHA-MW, iCBT+CHA-MW, and CHA-MW all demonstrated similar patterns of effect.

By contrast, decentering only had significant within-group differences from baseline to week 8 in the CHA-MW arm. Decentering, defined as the ability to observe one’s thoughts and feelings as transitory, objective events in the mind rather than as true reflections of the self [73], is a skill central to both mindfulness and cognitive-based therapies. Based on prior studies, which have demonstrated that decentering partially mediated improvements during MBCT [26,88,89], we had anticipated that MBCT-R would increase decentering. Unexpectedly, although decentering was cultivated in both active study arms, neither the MBCT-R+CHA-MW nor the iCBT+CHA-MW arms significantly increased decentering. However, given that decentering scores were significantly lower in the CHA-MW arm at baseline, interpretation of these findings may also be confounded by regression to the mean. Further research, including dismantling studies, is needed to better understand the differential impacts of CHA-MW, MBCT-R, and iCBT on decentering.

### Limitations

In addition to limitations already discussed, the following should be noted to contextualize our findings. First, while the intention-to-treat analysis, using MLE methods, allowed all 97 participants to be included in the final analyses, the study was underpowered to support additional subgroup analyses. For instance, the absence of detectable between-group differences may reflect limited statistical power. It remains possible that subgroups of participants derive greater benefit from higher-intensity intervention; however, a larger trial is needed to evaluate moderators. Second, the sociodemographic characteristics of our study sample, which was predominantly White, female, educated, and heterosexual, limit the generalizability of study findings to aspects of identity and experience represented by the sample. Additionally, all study interventions took place online, which required technology access and literacy, resources that are not equally distributed across the population, nor immune to self-selection biases. The study also included rigorous exclusion criteria, which may further limit the generalizability of findings. Third, the study involved multiple exploratory outcome measures, which may have felt burdensome to some participants and could have negatively affected assessment completion rates. Fourth, study measures were exclusively self-report and, as such, vulnerable to biases of mood, memory, and present perceived stress. They may also reflect common method variance, or overlapping variability that is attributable to factors related to the common assessment method used (eg, positive or negative affect and social desirability), rather than reflect true relationships among the outcomes measured. Fifth, regarding missing data, results could be biased because of the use of the missing completely at random and missing at random assumptions used by the MLE approach. Sixth, the study was not designed as a noninferiority trial and, as such, is not positioned to assert the inferiority of one intervention or another. Furthermore, while CHA-MW monitoring and response served as a low-intensity, nonspecific, control condition in this specific integrated primary care setting, the study did not involve a control in the sense of treatment as usual or a waitlist. Considering evidence supporting the spontaneous remission of depression over time [90], care should be taken in attributing observed changes to the effectiveness of the various study arms, particularly given the study’s focus on individuals with mild to moderate depression and its relatively brief, 24-month follow-up period. Seventh, while the content of the MBCT-R intervention directly aligned with that of evidence-based MBCT, its method of delivery was modestly adapted to a videoconference platform to allow for remote implementation to larger groups. Adaptation may limit the interpretability of the findings. Finally, the COVID-19 pandemic has had various temporal waves and cycles of high-intensity stress, which could have impacted engagement, intervention participation and adherence, and the overall effectiveness for some cohorts more than others.

### Future Directions

Considering the relative resource efficiency of CHA-MW, replication of our findings with a cost-benefit analysis may reveal significant savings on a per person basis for CHA-MW over MBCT-R+CHA-MW or iCBT+CHA-MW. Future research could compare the effectiveness and resource efficiency of CHA-MW with other existing population mental health triaging systems used within community health care systems (eg, collaborative care [91,92]). MBCT or iCBT assignments were randomized in this study, yet treatment matching might have improved the impacts of the mindfulness and iCBT interventions, although studies have not always shown this to be the case [93]. Any future iterations of MBCT-R could include brief individual interviews, based upon evidence in a recent study that found that adding brief individual interviews (which often happen formally or informally during in-person mindfulness-based interventions) can have a measurable difference on adherence and completion rates in online mindfulness-based interventions [94]. Further research may be able to determine whether CHA-MW reached a ceiling that resulted in the other interventions having, on average, negligible additional benefit, and whether low completion rates during the COVID-19 pandemic and/or MBCT-R–specific adaptations (eg, larger group sizes online) contributed to current findings.

Implementing programs similar to CHA-MW, which pair online psychoeducation newsletters with a resource-efficient, high-tech, and soft-touch digital population mental health monitoring and semiautomated stratified care algorithm that optimizes patient agency and engagement with responsive, precise, and evidence-based care, could help to prevent trajectories of progressive impairment in the context of large-scale surges in the demand for individual and community mental health services during periods of high population stress.

### Conclusions

Findings from this randomized controlled clinical trial conducted during the COVID-19 pandemic suggest that MBCT-R+CHA-MW, iCBT+CHA-MW, and CHA-MW were each effective in treating depression. However, this study failed to demonstrate significant differences in the effectiveness of MBCT-R+CHA-MW as compared with CHA-MW in treating depression. Intervention completion during the COVID-19 pandemic was low, which may have impacted the extent of the effect that these evidence-based, remotely delivered interventions could have on depression symptom severity. These findings advance current research and clinical paradigms by spotlighting the relative cost- and care-effectiveness of CHA-MW when online intervention engagement and adherence may be low, with the finding being consistent whether the online remotely delivered intervention is synchronous group or asynchronous individual. Given the lack of evidence supporting any additive benefits of MCBT or iCBT to CHA-MW, more definitive research, with a highly powered trial, is needed to examine whether CHA-MW, with its effectiveness at mental wellness monitoring and referring participants to existing telemental health options, may be sufficient for tackling rapidly increasing mental health care needs during large-scale stressor events.

## Supplementary material

10.2196/88388 Multimedia Appendix 1 Supplemental materials.

10.2196/88388 Multimedia Appendix 2 Trial protocol.

10.2196/88388 Checklist 1 CONSORT 2025 checklist.

## Abbreviations

AER: adverse event report
CAT-ANX: Computer Adaptive Testing for Mental Health Anxiety module
CAT-DEP: Computer Adaptive Testing for Mental Health Depression module
CAT-MH: Computer Adaptive Testing for Mental Health
CBT: cognitive behavioral therapy
CHA: Cambridge Health Alliance
CHA-MW: Cambridge Health Alliance MindWell
CONSORT: Consolidated Standards of Reporting Trials
iCBT: internet Cognitive Behavioral Therapy
MBCT: Mindfulness-Based Cognitive Therapy
MBCT-R: Mindfulness-Based Cognitive Therapy for Resilience
MLE: maximum likelihood estimation
MPI: multiple principal investigator
NCCIH: National Center for Complementary and Integrative Health
PTSD: posttraumatic stress disorder
REDCap: Research Electronic Data Capture
