# Supplementary material for: Effects of Remotely Delivered and Web-Based Interventions on Depression Severity During the COVID-19 Pandemic: 3-Arm Randomized Controlled Trial
Source: JMIR Ment Health. 2026 Aug 4;13:e88388. doi: 10.2196/88388 (PMC13436849; doi:10.2196/88388)
Supplement: Supplementary file 1 [file mental-v13-e88388-s002.pdf]

Remotely Delivered Programs Targeting COVID-19 Stress-Related Depression and Substance Use

CO-PRINCIPAL INVESTIGATORS

Zev Schuman-Olivier, MD
Carl Fulwiler, MD PhD

Tool Revision History
Version 3.4
Approved by IRB on June 25, 2020

Version 3.5: Version Date (December 18, 2020) Sent to IRB AME1/NCCIH for Approval

1. Adjusted page numbering in table of contents
2. Added Christopher Morse, Gabriella Conversano, Christina Luberto, Lydia Smith, Ian Concannon, Audrey Cabral, Thomas Fatkin, Fiona Kate Rice, Fernanda Greco-Quentel, Zindel Segal, Christian Webb, and Douglas Grainger to the protocol
3. Removed Brian Mullin from the protocol
4. Added time since stressor as a covariate in exploratory analyses
5. Included Zoom technical support during consent if a tutorial is requested by a participant
6. Changed gift card vendor from PNC to Tango Gift Cards
7. Edited administration of Expectancy and Credibility Survey to Week 2 instead of Baseline and change to 6-item version of EC survey
8. Included random COVID-19 IgG saliva sample during screening
9. Updated salivary preparation procedures to reflect the contents of packages and when these packages are shipped
10. Changed Tango Gift Card Survey duration to 1 minute
11. Updated exclusion criteria from CAT-PTSD >75 to CAT-PTSD>70,
12. Updated inclusion criteria from CAT-DI PHQ-9 equivalency between 10-20 to CAT-DI PHQ-9 equivalency between 10-19
13. Included Tango Gift Card administrative login to access participant data instead of quarterly reports
14. Included Droplr platform to upload screenshots directly to the study Google Drive in the event of a COVID-19 related lockdown
15. Edited participant communication to include outreach via research coordinator's device through CHA's secure remote network in the event of a COVID-19 related lockdown
16. Edited randomization blocks to include blocks of 10
17. Removed additional spacing and minor formatting edits
18. Improved schedule of evaluations to clarify screening visit times

Version 3.6: Version Date (February 8, 2021) (AME1 required changes for IRB)

1. Adjusted page numbering in table of contents
2. Removed Zindel Segal and Doug Grainger from study team
3. Removed reference to CAT-DI PHQ-9 for inclusion/exclusion criteria and focused on CAT-DI only for clarity. Clarified that it is 10-point rise in CAT-DI and not 5-point rise in PHQ-9 equivalency that will trigger review in CHAMindWell.
4. Updated throughout to reflect that the exploratory aspects of the study (salivary cytokine/COVID-19 samples and daily stress diaries) optional and each will be part of an optional sub-study and will not be considered requirements of the main study.
5. Removed EHR from baseline, weeks 4, 8, 12 in Schedule of Evaluations and clarified that EHR data will be reviewed at week 24 for number of televisits and referrals to psychiatric/substance use treatment that were made during 24 weeks.

6. Corrected grammar/typos throughout.
7. Removed Doximity, which is no longer used by CHA.
8. Clarified payments for those enrolled in optional diary sub-study and optional salivary study.

Version 3.7: Version Date (March 2, 2021) (AME1 Required Changes for IRB, Resubmission 2)

1. Removed Farah Samawi from protocol
2. Updated protocol version number in footer: V3.7
3. Added a statement clarifying details of the 2 sub-studies (when they will start, who will be eligible, etc.)
4. Removed references to daily diaries and affective reactivity as key components of the study design
5. Renamed section 6.2.3. "Participant Coding" rather than "Participant Coding and De-Identification"
6. Clarified optional salivary collection, optional daily diary collection, and participant reimbursement protocols throughout for consistency

Version 3.8: Version Date (March 11, 2021) (AME1 Required Changes for IRB, Resubmission 3)

1. Removed Farah Samawi from Team Study Roster
2. Updated Study Design and Figure 1 (Study Schema)
3. Removed comments

Version 3.9: Version Date (March 29, 2021) (AME2 Required Changes for IRB)

1. Added Danielle Giachos, Leah Howard, Kristen Kilgallen, and Esteban Da Cruz to Team Study Roster as Research Assistants (RAs)
2. Changed CAT-MH psychosis cutoff to >60, or a clinical diagnosis of active psychosis

Version 3.9.1 Version Date (April 27, 2021) (AME3 Required Changes for IRB)

1. Fixed footer (v7.4)
2. Replaced Farah Samawi with Gabriella Conversano
3. Replaced chamindwell@challiance.org email with resiliencestudy@challiance.org (page 9); added "If eligible…" to the answer "I agree to participate in the daily diaries."

Version 3.9.2: Version Date (June 15, 2021) (AME4)

1. Added Leah Howard and Danielle Giachos to study team members responsible for hosting study groups and obtaining informed consent
2. Removed Brian Mullin from Study Team Roster
3. Removed Christopher Morse from Study Team Roster
4. Removed Ian Concannon from Study Team Roster
5. Removed Fernanda Greco-Quentel from Study Team Roster
6. Removed Mark Albanese from Study Team Roster

Version 4.0 Version Date (August 13, 2021) (AME5)

1. Removed CAT-SS from CAT-MH and removed CAT-MH Psychosis (from study visits/analyses, but kept for screening purposes),

2. Clarified exclusion criteria to include MMHS groups (as a form of MBI)
3. Clarified salivary sub-study details (e.g., removed courier driver, participants will come to CMC pre- and post-intervention for salivary cytokine and COVID Antibody collection)
4. Changed 14 days of daily diaries to 5 diaries (over the course of 7 days, both pre-intervention and post-intervention) as part of the sub-study
5. Clarified language about Study Weeks -4-0
6. Updated IL-6 sub-study exclusion criteria to include inability to come to CMC for saliva sample collection due to logistical difficulty
7. Clarified participant recruitment details regarding lottery for gift card incentives
8. Removed mention of a separate COVID-19 Socioeconomic Impact survey from Baseline battery, since it only refers to items in another survey
9. Removed Home Skills Use survey from baseline survey battery in section 6.2.5.4.c.
10. Made changes to the Schedule of Evaluation:
    a. Updated the 'Diaries and Saliva Sampling (OPTIONAL)' section of the Schedule of Evaluation to clarify that COVID-19 and IL-6 samples will be collected between study weeks 9-12, and changed duration of saliva sampling to 15 minutes
    b. Removed Ranked Desire from Baseline surveys in the Schedule of Evaluation
    c. Removed monthly CAT-DI and CAT-ANX on study weeks 4 and 8 from Monitoring section to reflect the correct schedule (as at these weeks participants receive the monthly full CAT-MH survey battery)
11. Added Joseph Rosansky to Study Team Roster
12. Removed Kristen Kilgallen from Study Team Roster
13. Removed Miriam Tepper from Study Team Roster

Version 4.1 Version Date (February 23, 2022) (AME6)

1. Added Rocio Hernandez Chavez to Study Team Roster
2. Added Clare Bumpus to Study Team Roster
3. Added Savannah Rae Richard to Study Team Roster
4. Removed Danielle Giachos from Study Team Roster
5. Removed Timothy Creedon from Study Team Roster
6. Removed Esteban Da Cruz from Study Team Roster
7. Removed Alexandra Brunel from Study Team Roster

Version 4.2: Version Date (June 20, 2022) (AME7)

1. Added Gareth Parry to Study Team Roster
2. Removed Leah Howard from Study Team Roster
3. Removed Audrey Cabral from Study Team Roster
4. Expanded responsibilities of Clare Bumpus as Research Coordinator
5. Added that we may contact participants up to 12 months post-intervention for daily diary and salivary sample collection
6. Added relationship between daily diaries and IL-6 up to 12 months post-intervention as exploratory outcome for optional sub-study, as outlined in H3.2
7. Clarified consent procedures for participants invited to join sub-studies after main study completion
8. Clarified payment for participants invited to join sub-studies after main study completion
9. Removed spaces between CHA and MindWell (i.e., CHAMindWell)

Version 4.3: Version Date (August 03, 2022) (AME8)
1. Removed Kayley Okst from Study Team Roster
2. Added Javier Barria to Study Team Roster
3. Removed Tom Fatkin from Study Team Roster

Version 4.4: Version Date (March 16, 2023) (AME9)
1. Added that we may recruit participants to be healthy controls for the daily diary and salivary sample collection
2. Added screening and consent procedures for participants invited to join sub-studies as healthy controls
3. Added the Responses to Stress Questionnaire COVID-19 (RSQ-COVID-19) to the Diaries and Saliva Sampling section in the Schedule of Evaluations
4. Removed Gabriella Conversano from Study Team Roster
5. Removed Phil Wang from the Study Team Roster
6. Added all healthy control information to section 14 of the table of contents

Version 4.5: Version Date (June 1, 2023) AME10
1. Removed Lydia Smith from the Study Team Roster

Version 4.6: Version Date (June 7, 2024) AME11
1. Add Tori Blot as an administrative contact, removing Carl Fulwiler and Clare Bumpus as administrative contacts.
2. Remove Todd Griswold, Benjamin Cook, Ellie Grossman, Rocio Hernandez-Chavez, Clare Bumpus, Savannah Rae-Richard, Javier Barria and Christina Luberto from the protocol.
3. Update the original paper protocol study roster to reflect the correct study roster as appears in Cayuse.

Version 4.7: Version Date (November 27, 2024) AME12
1. This modification is to add Liv Valo to the study Team.
2. We have removed Richa Gwande from the study team
3. We have added updated training for Carl Fulwiler.
4. Update the original paper protocol study roster to reflect the correct study roster as appears in Cayuse.

TABLE OF CONTENTS
V4.7

	Page
**PRINCIPAL INVESTIGATOR**	**1**
**TABLE OF CONTENTS**	**6**
**TEAM STUDY ROSTER**	**9**
**PRÉCIS**	**12**
Objectives	12
Design and Outcomes	12
Interventions and Duration	13
Sample Size and Population	14
**1. STUDY OBJECTIVES**	**15**
**2. BACKGROUND AND RATIONALE**	**16**
2.1. Background on Condition, Disease, or Other Primary Study Focus	16
2.2. Study Rationale	18
**3. STUDY DESIGN**	**18**
**4. SELECTION AND ENROLLMENT OF PARTICIPANTS**	**19**
4.1. Inclusion Criteria	19
4.2. Exclusion Criteria	19
4.2.1. Exclusion Criteria for IL-6 sub-study	20
4.3. Participant Recruitment	20
**5. STUDY INTERVENTIONS**	**22**
5.1. Interventions, Administration, and Duration	22
5.1.1. Interventions	22
5.1.1.a. MBCT-R, Mindfulness-Based Cognitive Therapy Skills for Resilience During COVID-19	22
5.1.1.b. Internet Cognitive-Behavioral Therapy (iCBT)	24
5.1.1.c. CHAMindWell Monitoring and Support (CHA-MW)	26
5.1.2. Administration	26
5.1.3. Duration	27
5.2. Handling of Study Interventions	27
5.3. Concomitant Interventions	28
5.3.1. Allowed Interventions	28
5.3.2. Required Interventions	28
5.3.3. Prohibited Interventions	28
5.4. Adherence Assessment	28
5.4.1. Measurement and Reporting of Participant Accrual, Compliance with Inclusion/Exclusion Criteria	28
5.4.2. Measurement and Reporting of Participant Adherence to Treatment Protocol	28
5.4.3. Measurement and Reporting of MBCT-R Group Leader Adherence to Treatment Manual	29
**6. Description of Evaluations**	**32**
6.1. Screening Process	32
6.2. Enrollment, Final Screening, Baseline, and/or Randomization	32

6.2.1. Consenting Procedure 33
6.2.2. Consent Assessment 33
6.2.3. Participant Coding 33
6.2.4. Salivary Screening Assessments 34
6.2.4.a. Screening Oral Fluid Toxicology (Salivary) Testing (Weeks -4 – 0) 34
6.2.4.b. COVID-19 Salivary Testing 34
6.2.5. Baseline Assessments 34
6.2.5.a. Baseline Daily Tracking (Weeks -4 – 0) 34
6.2.5.b. IL-6 Cytokine Saliva Collection (Weeks -4 – 0) 35
6.2.5.c. Consent, Screening, and Baseline Survey Batteries (Week -4 and Week 0) 35
6.2.6. Randomization and Allotment 36
6.2.7. Blinding 36
6.3. Overview of Study Assessments 37
6.4. Participant Communication 37
6.5. Completion/Final Evaluation 38
6.6. Participant Reimbursement 38
7. SAFETY ASSESSMENTS 40
7.1. Specification of Safety Parameters 40
7.2. Methods and Timing for Assessing, Recording, and Analyzing Safety Parameters 40
7.2.1. Expected Risks 40
7.2.1.a. MBCT-R 40
7.2.1.b. iCBT 42
7.2.1.c. CHAMindWell Monitoring 42
7.2.1.d. Salivary Monitoring 42
7.2.2. Expected Benefits 42
7.3. Adverse Events and Serious Adverse Events 43
7.3.1. Definition 43
7.3.2. Classification of AE Severity 43
7.3.4. AE Attribution Scale 43
7.4. Reporting Procedures 43
7.4.1. Procedures for Collecting and Reporting Unanticipated Problems 44
7.4.2. Reporting Unanticipated Problems that are Adverse Events 44
7.4.3. Reporting Unanticipated Problems that are not Adverse Events 45
7.4.4. SAE Reporting 45
7.5. Follow-up for Adverse Events 45
7.6. Safety Monitoring 46
8. INTERVENTION DISCONTINUATION 46
8.1. Participant Withdrawal/Termination Criteria 47
8.2. Withdrawing/Group Discontinuing Procedures 47
8.3. New Information 48
9. STATISTICAL CONSIDERATIONS 48
9.1. General Design Issues 48
9.2. Sample Size and Randomization 48
9.2.1. Justification of Sample Size and Power Calculations 48
9.2.2. Treatment Assignment Procedures 49

9.3. Definition of Populations 49
9.4. Interim Analyses and Stopping Rules 49
9.5. Outcomes 50
9.5.1. Primary Outcomes 50
9.5.2. Secondary Outcomes 50
9.5.3. Exploratory Outcomes 50
9.6. Data Analyses 51
9.6.1. Primary Outcome 51
9.6.2. Secondary Clinical Outcomes 51
9.6.3. Secondary Stress-Related Affective Reactivity and Salivary Cytokine Outcomes 52
9.6.4. Exploratory Outcomes 54
10. DATA COLLECTION AND QUALITY ASSURANCE 54
10.1. Data Collection Forms 54
10.2. Data Management 62
10.3. Quality Assurance 67
10.3.1. Training 67
10.3.2. Quality Control Committee 68
10.3.3. Metrics 68
10.3.4. Protocol Deviations 68
10.3.5. Monitoring 68
11. PARTICIPANT RIGHTS AND CONFIDENTIALITY 69
11.1. Institutional Review Board (IRB) Review 70
11.2. Informed Consent Forms 70
11.3. Participant Confidentiality 70
11.4. Study Discontinuation 71
12. COMMITTEES 71
13. PUBLICATION OF RESEARCH FINDINGS 74
14. Addendum: Enrolling Healthy Controls Participants for Sub-studies only 75
15. REFERENCES 75

Personnel present during study procedure:
At least one of the following personnel will be present during study and group procedures:

Zev Schuman-Olivier, MD, Principal Investigator; Carl Fulwiler, MD, PhD Co-PI; or Research coordinators (Alexandra Comeau, Clare Bumpus).

Study team members responsible for the following activities:
At least one of the following personnel will be responsible for obtaining and documenting informed consent: Zev Schuman-Olivier, MD, Principal Investigator, Carl Fulwiler, MD, PhD; or Research coordinator (Alexandra Comeau, Clare Bumpus).

The following personnel will be responsible for providing on-going information to the study sponsor and the IRB: Zev Schuman-Olivier, MD, Principal Investigator, Carl Fulwiler, MD,PhD; or Research coordinator (Alexandra Comeau, Clare Bumpus).

At least one of the following personnel will be responsible for maintaining participants' research records: Zev Schuman-Olivier, MD, Principal Investigator, Carl Fulwiler, MD,PhD; or Research coordinator (Alexandra Comeau, Clare Bumpus).

Inclusion of study personnel:
This project is a randomized controlled trial research study built on top of a population health implementation program at CHA.

The research team would consist of the following: Zev Schuman-Olivier, MD (PI); Carl Fulwiler (Co-PI); Ellie Grossman (Co-I); Benjamin Cook, PhD who is a quantitative methodologist and Director of the Health Equity Research Lab, Cambridge Health Alliance/Harvard Medical School; Richa Gawande, PhD (Co-I); Christina Luberto, PhD (Co-I); Additional staff include Gareth Parry (Biostatistician); Fiona Kate Rice (Research Assistant); Todd Griswold, MD (Co-I); Alexandra Comeau (Senior Research Coordinator); David Almeida (Co-I); Jackie Mogel (Co-I); Christian Webb, PhD (Co-I); Joseph Rosansky, PhD (Post-doctoral Fellow), Rocio Hernandez Chavez (Research Assistant), Clare Bumpus (Research Coordinator), Savannah Rae Richard (Research Assistant).

Standard clinical staff in comparative effectiveness study:
To more closely approximate standard care in a comparative effectiveness, MBCT-R group leaders and standard program support staff (group volunteers), will be able to participate in the MBCT-R groups without being required to be added to the study protocol as part of the research team. MBCT-R group leaders and program support staff will not be informed about the design of the protocol, who is participating in the study, nor will they have access to any study information.

PARTICIPATING STUDY SITES

The study will be conducted online through the CHA Center for Mindfulness and Compassion with recruitment online and at 12 adult primary care sites associated with **Cambridge Health Alliance (**DUNS number: 805262995**).**

Data analysis for aspects of the study will be conducted online through the **Pennsylvania State University** (DUNS number: 00-340-3953).

PRÉCIS

Remotely Delivered Programs Targeting COVID-19 Stress-Related Depression and Substance Use

Objectives

We will conduct a comparative effectiveness RCT comparing two online programs with a low-impact control condition. Mindfulness-Based Cognitive Therapy for Resilience (MBCT-R), and iCBT, are online versions of evidence-based treatments for depression. MBCT-R integrates training in mindfulness with elements of CBT with a focus on the psychosocial stressors associated with COVID-19 that increase risk for depression. It is based on the 8-week MBCT program which has well-established efficacy for symptoms of depression, anxiety and stress, and for preventing relapse in recurrent depression. iCBT is a 6-week web-based asynchronous cognitive-behavioral therapy educational curriculum for depression and anxiety. The two programs will be compared with weekly monitoring by CAT-MH with telephone support and referral to treatment as needed. Primary outcomes are levels of depressive symptoms, substance use, and stress-related affect reactivity over the course of 24-weeks of completion of computerized adaptive mental health interviews (CAT-MH).

Design and Outcomes

Randomized control trial:
We are collaborating with CHA's extensive system of primary care to employ computerized adaptive testing (CAT-MH®) to rapidly screen and monitor patients during the pandemic. First, we are employing CAT-MH to identify patients at high-risk of stress-related mental illness, characterizing patients into 3 tiers: minimal symptoms who are low-risk (Tier 1), mild-to-moderate symptoms who are at-risk, but not meeting criteria for limited in-person treatment (Tier 2), and moderate-to-severe symptoms requiring treatment (Tier 3). Tiers are based on the CAT-DI, which has PHQ-9 equivalency scores, with well-established cut-offs for treatment recommendations in primary care of mild (5-9, watchful waiting), moderate (10-14, psychological treatment), moderately severe (15-19, psychological and antidepressant treatment), severe (>20, referral to mental health treatment).[1] CAT-DI 50-75 is equivalent to 10-19, representing moderate and moderately severe depression.

Second, Tier 2 patients who have mild-to-moderate symptoms of depression (CAT-DI PHQ-9 equivalency between 10-19) will be recruited for the study and will be randomized in a 3-arm comparative effectiveness RCT to compare a live, online psychoeducational program - Mindfulness-Based Cognitive Therapy for Resilience (MBCT-R) – to either a) enhanced monitoring (with weekly CAT-MH monitoring with rapid referral to in-person mental health care if needed, or b) an asynchronous internet CBT (iCBT) application. MBCT is as effective as maintenance antidepressant medication for preventing relapse in recurrent depression and is also

effective for reducing current symptoms of stress, anxiety and depression. We are currently implementing a MBCT-R pilot project with 17 patients enrolled in CHAMindWell with ongoing CAT-MH monitoring during COVID-19. We will randomize to MBCT-R + CHAMindWell : iCBT + CHAMindWell : CHAMindWell monitoring alone in a 2:2:1 ratio.

The following paragraph describes an optional sub-study and associated measures. Since this aspect of the study is exploratory and may not be available throughout the study, it will only include a subset of participants who enroll during the time this sub-study is running. However, we may invite participants who will either be healthy controls or participants who have enrolled in the study, including those who previously completed the study when the salivary sub-study was not available, to provide daily diaries and salivary cytokine and COVID-19 samples for up to 12 months following intervention completion. Participants who are invited to complete the sub-study after having completed the main study or those who are recruited to the sub-study as healthy controls (see section 14 for healthy controls) will only provide at least 5 out of 7 daily diaries and one saliva sample at a single timepoint, which will be considered post-intervention data.

We will use optional daily diaries before randomization, at 9-12 weeks, and up to 12 months following intervention completion to investigate changes in affective reactivity during stressors. We will also examine the relationship between stress, affective reactivity (uptick in negative affect or downtick in positive affect on stressor days over 7 diary days), and levels of interleukin-6, as well as how these factors relate to changes in depression severity. Daily Stressors will be assessed using the Daily Inventory of Stressful Events (DISE[2]), which yields several variables for each reported stressor including: (a) content classification of the stressor; (b) who was involved in the event; (c) dimensions of threat (loss, danger, disappointment, frustration, opportunity); (d) and subjective severity. We have adapted this inventory to include COVID-19 related stress. Affect will be assessed using Positive and Negative Affect scales developed for the national MIDUS Study[3,4]. These synergistic initiatives offer a unique opportunity to study the effects of online, live MBCT-R on depression, substance use, stress-related affective reactivity, and stress-related inflammation.

Interventions and Duration

<u>MBCT-R:</u>
Mindfulness-Based Cognitive Therapy (MBCT) is an effective group intervention for depression and anxiety that combines mindfulness training with elements of cognitive therapy. The manual-based intervention consists of training in formal and informal mindfulness practices to bring mindful awareness to the relationship between thoughts, feelings and body sensations in daily life, and group discussion of practices and homework. MBCT-R is a slight modification of MBCT designed to foster resilience and prevent development of new-onset anxiety and depression disorders or the exacerbation of existing conditions. The structure of eight 2 1/4 hour classes and one half-day retreat is the same, but the class is delivered online and addresses the specific stressors associated with the COVID-19 pandemic and its economic and social consequences. MBCT-R retains the basic MBCT curriculum including at-home assignments to engage in specific meditation practices daily and audio instructions are provided. The theme of

resilience in the face of novel stressors associated with the COVID-19 pandemic is woven throughout. Classes of 40-50 participants will be co-taught by Co-PI Fulwiler, Director of MBCT Training at the Center for Mindfulness and Compassion, and an additional trained group leader, that may not be listed in this protocol, following the MBCT-R manual. After completing training and co-leading at least 2 classes with Dr. Fulwiler (along with mentoring), these group leaders will be eligible to lead an MBCT-R class on their own with an additional co-leader. MBCT-R participants will also be enrolled in CHAMindWell Monitoring and Support.

iCBT:

iCBT is a family of evidence-based online programs for depression, anxiety, stress and general psychological well-being[5]. MoodGYM, was developed by the Centre for Mental Health Research at the Australian National University[6] and is based on CBT and interpersonal therapy targeting depression, anxiety, stress, and general psychological distress. MoodGYM has 6 sessions with five curriculum modules and a review session that can be completed within an 8-week period. iCBT participants will also be enrolled in CHAMindWell Monitoring and Support. If there are difficulties with accessing iCBT technology or if monitoring with CAT-MH or CAT-DI/ANX-CAT interviews suggests symptomatic worsening, then a CHAMindWell coordinator will contact the participant and check-in and offer support, including setting up a televisit with a member of a clinical member of the outpatient mental health team if needed.

CHAMindWell Monitoring and Support:

All groups will have CHAMindWell Monitoring and Technician support during weeks 1-8 and then every 4 weeks afterwards for 24 weeks. They will complete CAT-DI and ANX-CAT weekly and full CAT-MH every 4 weeks. Trained technicians will review scores weekly and reach out to anyone whose CAT-MH severity level increased, anyone who increased by more than 5 points on CAT-DI PHQ-9 equivalency from baseline, or anyone who remains at a moderate level of depression for 2 or more weeks in a row. Participants with severe symptoms or worsening moderate symptoms over 2 weeks will be asked if they would like a televisit with a clinician.

Sample Size and Population

We anticipate enrolling N=240 participants over 8 months. We anticipate 200 participants to be randomized (2:2:1 ratio, block size of 5 to MBCT-R, iCBT, or CHA MindWell alone, respectively) (n=80 MBCT-R + MW, n=80 iCBT + MW, n=40 CHA MindWell alone).

Participants will be patients at CHA and must be 18-70 years old. This study will enroll individuals of any gender, and any demographic group, including pregnant women.

Participants must have CAT-DI scores of 50-75 (Tier 2 patients), with sufficient English fluency to understand procedures and questionnaires, without acute severe mental illness (active psychosis, bipolar I disorder, acute suicidality or self-injurious behavior, severe depression, severe PTSD, moderate to severe substance use) and ability to provide informed consent. This trial is limited to English-speaking participants at this time since the intervention curricula have

not been adapted to other languages. As this is an internet-based trial, participants must also have access to the internet and an electronic device in order to attend study groups and complete online questionnaires.

1. STUDY OBJECTIVES

1.1. Examine effects of remotely delivered programs on depression by randomizing patients with mild-moderate depressive symptoms to MBCT-R, iCBT, or CHA-MW monitoring alone (N=200, 80:80:40 per arm).

H1.1: MBCT-R will show greater efficacy compared with CHA-MW monitoring alone on depressive symptoms as measured by CAT-MH during 24 weeks after randomization. (*Primary Aim*)

H1.2: iCBT will show greater efficacy compared with CHA-MW monitoring alone on depressive symptoms as measured by CAT-MH during 24 weeks after randomization. (*Secondary Aim*)

1.2. Examine effects of remotely delivered programs on substance use by randomizing Tier 2 patients to MBCT-R, iCBT, or CHA-MW monitoring alone.

H2.1: MBCT-R will be more effective than other arms with less alcohol use (fewer heavy drinking days per month using timeline follow-back) at 24 weeks. (*Secondary Aim*)

H2.2: MBCT-R will be more effective than other arms with less drug use (fewer positive oral fluid toxicology screens for illicit substances) at 24 weeks. (*Secondary Aim*)

1.3. Examine effects of remotely delivered programs on televisit utilization by randomizing patients with mild-moderate depressive symptoms to MBCT-R, iCBT, or CHAMindWell monitoring alone (N=200, 80:80:40 per arm).

H3.1: MBCT-R will require lower numbers of televisits MH during 24 weeks after randomization compared with CHAMindWell monitoring alone. (*Secondary Aim*)

H3.2: MBCT-R will require lower numbers of televisits MH during 24 weeks after randomization compared with iCBT. (*Secondary Aim*)

1.4. Optional Sub-Study Exploratory Aim: Examine the relationship of stress-related affect reactivity and inflammation on depression.

H4.1: Higher salivary IL-6 will be associated with greater depression severity. (*Exploratory Aim*)

H4.2: Reductions in salivary IL-6 and stress-related affective reactivity will partially mediate the effect of MBCT-R on depression severity. (*Exploratory Aim*)

1.5. Additional Exploratory Objectives

We will include several exploratory outcomes [anxiety, perceived stress (PSS-14), neurovegetative symptoms (BDI-2), rates of clinician televisit use, referral rates to psychiatric/substance use treatment, rates of in-person MH visits, additional inflammatory

markers in the salivary sub-study (TNF-a, IL-1b, IL-8)]. We will include several covariates [age, race/ethnicity, gender, COVID-19 status (when available), COVID-19 impact (socio economic from RSQ COVID-19 survey), COVID-19 fear, alcohol drinks per day, cannabis use, nicotine use, and time elapsed since stressors] and exploratory mechanisms [experiential avoidance, stress coping style (disengagement, involuntary dis/engagement), rumination, emotion regulation, decentering, self-compassion, and interoceptive awareness].

2. BACKGROUND AND RATIONALE

2.1. Background on Condition, Disease, or Other Primary Study Focus

The COVID-19 pandemic presents a level of everyday stress unparalleled in modern history, with significant implications for mental health, substance use and treatment capacity. It is well-established that natural disasters lead to increased rates of mental illness, and the COVID-19 pandemic and its aftermath is already proving to be no exception[7–10,11]. In the aftermath of Hurricane Katrina in 2005, rates of anxiety-mood disorders nearly doubled[12] and there was a surge in mental illness that overwhelmed mental health systems[1314]. Only 18% of people with new onset symptoms qualifying for a mental health diagnosis sought mental health care during the next year[15] and most of those who did seek care presented to primary care[16]. Substance use disorders and treatment admissions increased among both white[17] and disadvantaged minority populations[18], with 20% of all substance use disorder admissions also having psychiatric illness[19], showing the impacts of ongoing stress and untreated mental illness. Similarly, after the Baton Rouge flood in 2016, psychiatric visits more than doubled in the year after the flood. The largest impact on the behavioral health care system was from increases in depression (190%) and substance use (330%) visits, which together accounted for 49% of all mental health visits during the year[20]. People with acute socioeconomic impacts had a higher risk of depression[21]. As the COVID-19 disaster unfolds, urgent steps with a population health approach are needed to bolster the mental health system with prevention and monitoring efforts in anticipation of the inevitable increase in mental health symptoms and need for treatment.[9]

Stress is a major cause of new-onset depression and substance use, and it is also a predictor of relapse in both disorders. The pathophysiology of stress in precipitating depression and in provoking relapse is an area of active research. While overall number and severity of stressors predicts the emergence or recurrence of affective disorders, some people are resilient during stress and do not develop a disorder[22, 23]. In particular, upticks in levels of negative affect to daily stressors predict the likelihood of having an affective disorder 10 years later[24], and negative affect reactivity to stress predicts mortality among people with chronic illness[2]. This suggests the way one copes with stress may reduce its physical and mental impact. Developing a better scientific understanding how stress leads to depression, how resilience factors help people to avoid stress-related depression and developing remote interventions that support resilience and can be rapidly disseminated fills a critical public health need during this time of crisis.

It is estimated that more than 300 million people in the world suffer from depression, which is listed by the World Health Organization (WHO) as the single largest factor contributing to global disability[25] with the incidence increasing over recent decades in the U.S.[26] The frequently chronic and recurrent nature of depression and the fact that many patients experience significant residual symptoms after remission contribute significantly to disability.[27,28]. Mindfulness-Based Cognitive Therapy (MBCT) was adapted from Mindfulness-Based Stress Reduction (MBSR) to target depressive symptoms and risk factors by integrating training in mindfulness with elements of cognitive therapy.[29] . Several randomized controlled trials showed that MBCT reduced relapse rates to usual care[30–32], and as effective as maintenance antidepressant medication[32–34]. In some studies, MBCT was more effective than antidepressants, especially in patients with residual depression symptoms.[34,35] In addition to its original application for prevention, there have been a number of studies showing efficacy of MBCT among people who are currently experiencing depression or elevated symptoms of depression[36–38], and a recent meta-analysis of 13 trials found that MBCT was as effective as other active treatments for people with current depressive symptoms[39]. Additional studies of MBCT have demonstrated efficacy for anxiety[40–42] and stress[43,44].

Given robust evidence for the effectiveness of MBCT for prevention of depression as well as amelioration of active symptoms of depression, anxiety and stress, this cost-effective group program which can be delivered effectively online[42,45]is an ideal preventative strategy. MBCT is designed to target maladaptive rumination – a persistence of negative, self-focused thinking – that characterizes depression, based on studies showing that patients who demonstrate greater cognitive reactivity to low mood are more likely to experience relapse.[46–48]Subsequent research has confirmed that reduced cognitive reactivity is a central mechanism for the effectiveness of MBCT[49]. These cognitive tendencies can be remediated using mindfulness practice to facilitate an earlier recognition of unhelpful thought patterns and unpleasant feelings that usually trigger rumination. The manual-based intervention consists of training in formal and informal mindfulness practices to bring mindful awareness to the relationship between thoughts, feelings and body sensations in daily life, and group discussion of practices and homework. It is taught in eight 2 1/4-hour classes and a half-day retreat. The curriculum includes assignments to engage in the meditation practices daily and audio instructions are provided.

Cambridge Health Alliance (CHA) is a safety-net healthcare system serving the metro-north Boston area, a combined population of over 380,000 residents, with the highest per capita COVID-19 rates in Massachusetts, which is 3rd in the US in overall number of COVID-19 cases per state. 60% of CHA patients receive federally subsidized health insurance.

2.2. Study Rationale

This study offers four innovations that will shift current research and clinical paradigms:

1. Innovative Application of Evidence-Based MBI to Address the Mental Health Impact of COVID-19: MBCT-R is based on the 8-week MBCT program which has the strongest evidence base of all mindfulness-based interventions (MBI) for prevention, shown to reduce symptoms of anxiety and depression, and is the only MBI shown to be preventative, i.e., it is as effective as maintenance antidepressant medication for reducing risk of relapse in people with recurrent depression. MBCT-R is innovative because it incorporates a focus on the specific stressors associated with the COVID-19 pandemic, teaching practical mindfulness and CBT skills for coping and staying well.

2. Optimized for Patient Empowerment and Self-Monitoring: Using CAT-MH interviews to identify patients at risk of exacerbation or relapse (Tier 2), we will offer MBCT-R and iCBT as evidence-based approaches to prevention, thereby reducing the impact of COVID-19 on our vulnerable population of low-income patients. Furthermore, the implementation of CAT-MH monthly monitoring surveys, provides a quick platform for providers to monitor patient mental health and refer patients to the appropriate level of care they require.

3. Population Health Focus: MBCT-R is designed for implementation within primary care and outpatient mental health and will also be used with CHA's two ACO populations. By providing it as a psychoeducational group live online, MBCT-R also allows us to reach a much larger audience than conventional group therapy models. Repeated CAT-MH monitoring allows us to safely monitor large numbers of participants and identify those who may need a higher level of care.

4. Innovative Methods for Elucidating Mechanisms of MBI for Prevention: Testing novel hypotheses about stress related to psychological and biological methods of prevention. Collecting daily diaries and salivary samples from a subset of participants as optional measures will allow us to examine the mechanistic relationship of stress-related affect reactivity and inflammation as potential mediators of depression.

3. STUDY DESIGN

We will randomize to MBCT-R + CHAMindWell : iCBT + CHAMindWell : CHAMindWell monitoring alone in a 2:2:1 ratio. Changes in depression symptom severity (primary outcome), measured by the CAT-DI, will be assessed at weeks 0, 4, 8, 12, 16, 20, and 24. Rates of positive toxicology screens and levels of self-reported substance use on 30-day TLFB (secondary outcomes) will be assessed at 24 weeks. We will also track the number of mental health clinician televisits required during the first 12 weeks and the full 24 weeks of the study (secondary outcome).

The 2 optional sub-studies (daily diaries and salivary collection) will begin running once in-person saliva collection protocols are fully approved by the CHA Laboratory. Originally, we had planned to have participants collect IL-6 and COVID-19 saliva at home to store and be picked up later by a courier driver. However, saliva is now considered to be a Category B substance (due to the ongoing COVID-19 pandemic), and

thus we will not be able to use a courier driver to pick up the samples. Therefore, we have adapted our protocol so that salivary collection for the optional sub-studies will occur at the CMC, where a trained research coordinator will collect the saliva samples and immediately store them in a locked CMC freezer.

Once the procedures for the 2 optional sub-studies have been finalized and approved, anyone who newly enrolls in the main study will be eligible to participate in the optional sub-studies, as long as they do not meet the exclusion criteria for the IL-6 sub-study (i.e., signs of poor oral health, dental problems, logistical difficulties) and are willing and able to come to the CMC in person for saliva collection.

Figure 1. Study Schema

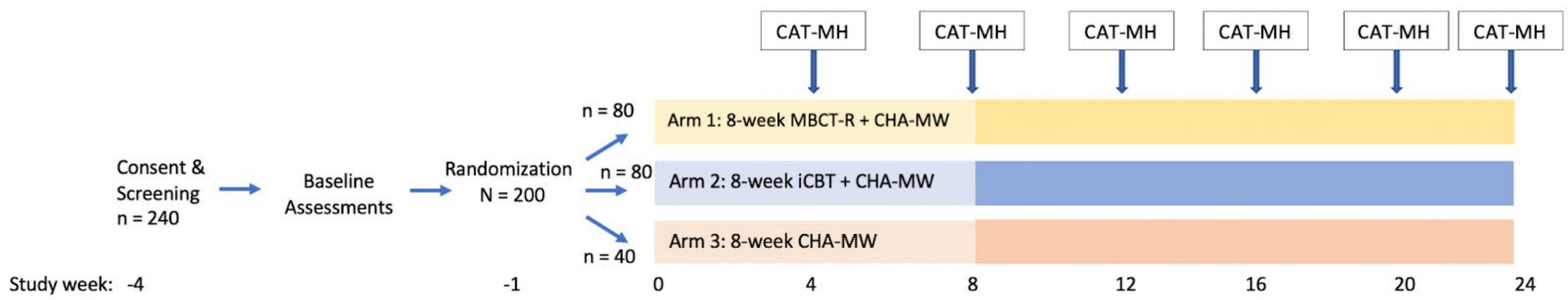

4. SELECTION AND ENROLLMENT OF PARTICIPANTS

4.1. Inclusion Criteria

All of the following are required criteria for inclusion in the study:

5. 18-70 years old
6. Current patient of CHA primary care or behavioral health provider
7. Active enrollment in CHAMindWell
8. Sufficient English fluency to understand procedures and questionnaires
9. Ability to provide informed consent
10. Access to the internet and an electronic device to attend study groups and complete questionnaires.
11. CAT-DI 50-75

4.2. Exclusion Criteria

Any of the following is regarded as a criterion for exclusion from the study:

12. Active psychosis or severe level of psychosis on CAT-Psychosis (>= 60)

13. Bipolar I disorder history or severe level of mania on CAT-M/H[50] (>70)
14. Acute suicidality or self-injurious behavior or severe level of suicidality on CAT-SS[51] (>=71)
15. Severe depression, indicated by CAT-DI > 75[50,52]
16. Severe level of PTSD on CAT-PTSD[50] (>70)
17. Current treatment with antipsychotic medication, mood stabilizer or benzodiazepine equivalent of 3mg/day of lorazepam
18. Cognitive inability as demonstrated by the inability to complete an online informed consent assessment
19. Current participation in another experimental research study
20. Previous participation in an 8-week intensive Mindfulness-Based Intervention in past 1 year, including Mindful Mental Health Service (MMHS) groups (e.g., MTPC, MSC, MBSR)
21. Expected medical hospitalization in next 6 months
22. Expected incarceration in next 6 months
23. Severe substance use disorder or high risk on CAT-MH SUD. In addition, use of or positive toxicology for cocaine, unprescribed opioids, stimulants, or benzodiazepines in the past 3 months.
24. Inability to participate in group intervention without disrupting group in opinion of principal investigator
25. Inability to complete screening, baseline assessments and 5 daily diaries.

4.2.1. Exclusion Criteria for IL-6 sub-study

Any of the following is regarded as a criterion for exclusion from the sub-study:

1. Signs of poor oral health (e.g., oral infections, teeth bleeding when brushed, etc.) as indicated by the UCI Oral Health Questionnaire
2. Dental Problems as indicated by the UCI Oral Health Questionnaire
3. Logistically difficult for them to do this part of the study (e.g., inability to come to the CMC for saliva sample collection)

4.3 Participant Recruitment

To ensure a rigorous comparative effectiveness design, we would refer to the study during recruitment as a "study comparing remotely delivered interventions designed to promote mental wellness and reduce stress-related depression, anxiety, and substance use," which would avoid the impact of expectation bias towards or against mindfulness, CBT, or monitoring alone.

All recruitment for the study will happen through CHAMindWell, the current standard offering at CHA for population mental wellness monitoring during COVID-19. All patients must already be enrolled and active in CHAMindWell with a completed CAT-MH to be referred to or eligible for inclusion in the study. All patients who complete a CHAMindWell CAT-MH assessment and

score between 50-75 on CAT-DI will be contacted by the research coordinators about the study. People who have registered for CHAMindWell who have had a score ≤35 on the CAT-DI and who do not have any other CAT-MH screening test >50 may be contacted by the research coordinators about their interest in the sub-studies, to be screened to participate as a healthy control. This contact by the study team will either be through MyCHArt or whichever means a patient consented for use in CHAMindWell, including phone, email (including a color flyer describing details of the study), or text (for patients who indicated to CHAMindWell that text is appropriate for communication). Information about the study will be posted on the CHA CMC website after IRB approval of the patient flyer and will be shared with CHA staff electronically.

CHA patients may end up learning about and enrolling in CHAMindWell through various pathways related to the underlying population mental health initiatives in the CHA Department of Psychiatry, which is beyond the scope of the study. They may be referred to CHAMindWell by their CHA primary care provider or a CHA behavioral health provider. Patients might also self-refer to CHAMindWell or be encouraged to enroll in CHAMindWell by CHA's ACO. There are several ongoing and emerging population mental health initiatives in the Department of Psychiatry, so we anticipate building substantial demand for the CHAMindWell program during the COVID-19 surge through these avenues. These include the following:

- All adult ACO patients (~65,000 CHA ACO patients) will receive an email communication from Cambridge Health Alliance about the benefits of enrolling in the CHAMindWell community mental wellness support program during COVID-19. This will include a gift card incentive for adult patients with CHA PCPs who enroll in CHAMindWell and complete an initial CAT-MH computerized assessment.
- CHA Providers will refer patients to standard CHAMindWell for monitoring with CAT-MH or to MBCT-R or iCBT using EHR. Patients that are referred to MBCT-R or iCBT will concurrently be enrolled in enhanced CHAMindWell monitoring. Patients will only be eligible for study recruitment after they are enrolled in some form of CHAMindWell monitoring.

<u>Procedures for recruitment and screening eligible patients (e.g., Screening Log).</u>
CHAMindWell patients who have CAT-DI scores 50-75 may be referred to the study by CHAMindWell coordinators. A trained Research Coordinator (RC) or research assistant (RA) will also review the patients' most recent CAT-MH interview (within the last 30 days) that they have completed through the CHAMindWell program. CAT-MH will be reviewed in screening to identify psychiatric risk factors and diagnoses[50,53], and a review with a doctoral level clinician will be required if there is possible psychiatric exclusion.

If the CAT-DI score was collected more than 30 days prior to the date of the main resilience study eligibility review, study staff will review the CAT-DI score from the study's baseline assessment for eligibility scores during screening. If the new CAT-DI score from baseline is no longer within the 50-75 range, participants will still be eligible for the main resilience study ONLY IF their baseline score is equal to or greater than 35. All other participants will be deemed ineligible at that point in time and referred back to

the CHAMindWell program. These participants may be reconsidered for the study in the future if their scores fall within the eligibility range.

Additionally, to ensure study eligibility based on other mental health symptoms and diagnoses, a trained research coordinator or research assistant will review the survey scores in the patients' most recent full CAT-MH interview that they have completed through the CHAMindWell program. CAT-MH interview scores must be taken within the last 90 days. If the full CAT-MH interview was completed more than 90 days prior to the time of study eligibility review, a research study team member will alert trained coordinators from the CHAMindWell program to request that the patient complete a new CAT-MH interview. The research coordinator will use the new CAT-MH interview scores (within a 90-day range) to review for study eligibility.

Decisions on the inclusion/exclusion will be made by the research coordinator with support from the CMC medical director, Co-PI, and Principal Investigator. If the participant meets inclusion and exclusion criteria, then the RC, RA, study clinicians, and/or CMC referral recruitment coordinator will contact the patient and suggest scheduling a study consent/screening visit. During consent/screening, the Research Coordinator will offer Zoom technical support to ensure that all participants have adequate training for observed oral fluid toxicology testing and in the event that they are randomized to the MBCT-R + CHA-MW intervention. Time spent with support staff will be recorded.

During this visit the patient will be given a verbal overview of the key information related to the study by the RC, and an opportunity to consent to enrollment. Once a patient completes the consent process by scoring at least 90% on the consent assessment and signs the consent form, he/she will be referred to as a "participant" in the study.

After informed consent, a study research coordinator or trained research assistant will conduct an electronic health record (EHR) screen and final eligibility screening by reviewing recent clinical diagnosis and medication doses.

5. STUDY INTERVENTIONS

5.1. Interventions, Administration, and Duration

5.1.1. Interventions:

5.1.1.a. MBCT-R, Mindfulness-Based Cognitive Therapy Skills for Resilience During COVID-19

Resilience is the ability to respond to adversity, something we all need in these challenging times. **Mindfulness-Based Cognitive Therapy for Resilience (MBCT-R)** is a psychoeducational group program that incorporates teaching of mindfulness and CBT skills to foster resilience, reduce symptoms of anxiety and depression and prevent

relapse. It addresses the specific stressors associated with the COVID-19 pandemic and its economic and social consequences, integrating practical mindfulness skills with practical tools from cognitive therapy for coping and staying well. Through the practice of mindfulness participants learn to engage the body, mind, and heart to relate differently to challenging thoughts and emotional distress, to help to care for themselves and cultivate resilience. This live online program allows participants to engage with the teacher and with each other. This is an 8-week 2 ¼ hour program and includes a half-day retreat between weeks 6 and 7.

The manual-based intervention consists of training in formal and informal mindfulness practices to bring awareness to the relationship between thoughts, feelings and body sensations in daily life, and group discussion of practices and homework. It is taught in eight 2.25-hour classes and one half-day retreat in a classroom format. MBCT has a standard curriculum and flow (see Table 1), though course content often varies around 20% based on adapting content to current needs of participants and what is arising. Adherence and fidelity are monitoring by recordings via Zoom. The program includes at-home assignments to engage in the meditation practices daily and audio instructions are provided. The program consists of 8 weekly 2 ¼ hour sessions and a half-day retreat delivered live via video streaming (Zoom software), which allows real-time interactions between teachers and participants. Technical support is provided to participants as needed but this is rare. Supporting materials including readings, instructions for home practice, and recorded audio guided practices delivered on a secure web portal. MBCT-R participants will also be enrolled in CHAMindWell Monitoring and Support. If there are difficulties with accessing MBCT-R technology or if monitoring with CAT-MH or CAT-DI/ANX-CAT interviews suggests symptomatic worsening, then a CHAMindWell coordinator will contact the participant and check-in and offer support, including setting up a televisit with a clinical member of the outpatient mental health team if needed.

Table 1a: MBCT-R Sessions

Session	Title and Themes	Content
Session 1	Awareness & Automatic Pilot<br>· Mind-wandering<br>· Habituated behaviors<br>· Present moment awareness	· Raisin exercise<br>· Body scan<br>· Mindful inquiry<br>· Short breath focus
Session 2	Living in Our Heads<br>· The wandering mind<br>· Obstacles to paying attention<br>· The breath as an anchor	· Body scan<br>· Mindful inquiry & home practice review<br>· Thoughts and feelings exercise<br>· Short sitting meditation (breath/body)
Session 3	Gathering the Scattered Mind<br>· Lost in thought<br>· Welcoming & curious attitude<br>· Bringing mindfulness to daily life	· Sitting (breath/body/sensations)<br>· Mindful stretching/movement<br>· 3-minute breathing space<br>· Mindful inquiry & home practice review

Session 4	Recognizing Aversion<br>· Avoidance and attachment<br>· Turning towards the difficult<br>· A different place to view thoughts	· Sitting (breath/body/sounds/ thoughts)<br>· Checklist of negative thoughts<br>· 3-minute breathing space responsive<br>· Mindful walking<br>· Mindful inquiry & home practice review
Session 5	Allowing and Letting Be<br>· A different relationship to the unwanted<br>· Acceptance<br>· Moving towards self-care	· Sitting meditation focus on difficulty<br>· Mindful inquiry & home practice review<br>· Working with difficulty<br>· Breathing space
Session 6	Thoughts are Not Facts<br>· How moods influence thoughts<br>· Thoughts as mental events<br>· The participant observer	· Sitting meditation focus on thoughts<br>· Moods & thoughts<br>· Discuss relapse signature<br>· Discuss retreat
Retreat	Making the Practice Your Own<br>· Being in silence<br>· Going deeper with mindfulness<br>· Strengthening the practice	· Mindful walking<br>· Loving kindness meditation<br>· Discussion of practices<br>· Discuss preparation for end of course
Session 7	How Can I Best Take Care of Myself?<br>· Behavior and mood are inter-related<br>· Skills to prevent relapse<br>· Fostering resilience	· Sitting meditation (all)<br>· Exploring links between activity and mood<br>· Scheduling activities and mindful action<br>· Relapse prevention
Session 8	Maintaining & Extending New Learning<br>· Ending and beginnings<br>· The importance of self-care<br>· Maintaining well-being and resilience<br>· What is most important to us	· Body scan<br>· Review relapse prevention plans<br>· Review of entire course<br>· Keeping practice going<br>· Closing exercise

5.1.1.b. Internet Cognitive-Behavioral Therapy (iCBT) -- MoodGym

iCBT is a family of evidence-based online programs for depression, anxiety, stress and general psychological well-being[5]. MoodGYM, was developed by the Centre for Mental Health Research at the Australian National University[6]. MoodGYM was chosen for six reasons. First, there exists substantial (although not conclusive) evidence supporting MoodGYM's effectiveness for symptoms of depression, anxiety, and general psychological distress[54], including reductions in hazardous alcohol use[55] and suicidal ideation[56]. Second, MoodGYM is readily available for purchase as an educational program on the Internet. Third, MoodGYM has 6 sessions with five curriculum modules and a review session that can be completed within an 8-week period similar to MBCT-R, which requires attendance in at least 6 sessions over 8 weeks to complete the program. Fourth, MoodGYM is a suitable comparator intervention based on CBT and interpersonal therapy to serve as an active CBT comparator for MBCT-R. Fifth, during COVID-19, participants may experience new onset symptoms of not just depression but also other symptoms, so an intervention for those experiencing symptoms of depression, anxiety, stress, and general psychological distress, could be accessible and useful to a high

number of participants. Fifth, no studies evaluating MoodGYM had previously been conducted in the US within a population mental health setting during COVID-19.

All participants will be given an access code token when they are randomized to iCBT, so that they can use it to enroll in Mood Gym. This will make the iCBT program free. eHub Health Pty Limited (the company that hosts MoodGYM, eHH) will provide the Research Coordinator and Study PI with administrative login credentials to access data about the Users' use of the Program, including depression and anxiety quiz scores, progress through the site (with date-stamps for when modules are started/completed), number of logins, and time in each module. This data will be keyed by the unique token identifiers which are entered by Users when they register. Completion of the program will be self-paced, with the exception that participants will be encouraged to wait at least 5 days in between consecutive lessons and complete all lessons within 8 weeks. After presenting the study coordinator with evidence of completion of all modules, then they will be eligible to receive the completion bonus at the end of the study. iCBT participants will also be enrolled in CHAMindWell Monitoring and Support. If there are difficulties with accessing iCBT technology or if monitoring with CAT-MH or CAT-DI/ANX-CAT interviews suggests symptomatic worsening, then a CHAMindWell coordinator will contact the participant and check-in and offer support, including setting up a televisit with a member of a clinical member of the outpatient mental health team if needed.

Table 1b. iCBT Mood Gym Structure and Content (from Twomey, et al.)[54]

Table 1. Structure and content of MoodGYM.

Sessions		Content
Number	Title	
–	*Introduction*	User completes brief outcome measures and the results of these are presented.<br>Six fictional characters/personas (in cartoon style) are introduced.<br>Throughout the programme, scenarios involving these characters are used to explain CBT principles relating to mood and thoughts.<br>The core concepts of CBT and 'warpy' thoughts are explained.<br>The programme worksheets and the online workbook are explained.
1	*Feelings*	Learning about negative thought patterns, biased perceptions, negative views about the self and future and the link between thoughts and feelings
2	*Thoughts*	Learning how to identify and challenge biased or 'warpy' thoughts<br>Identifying areas of vulnerability (e.g. the need to be loved)<br>Learning about self-esteem<br>Completing 'Being Nice to Myself for a Change' pleasant activity scheduling diary
3	*Unwarping*	Taking the role of a reporter and reporting on thoughts in the third person<br>Talking self out of dysfunctional thoughts<br>Setting up thought challenging experiments<br>Increasing social and physical activity<br>Testing out new ways of responding to situations
4	*De-stressing*	Learning about stress and identifying stressors<br>Take part in the 'relax fest' game show – a quiz about relaxation<br>Relaxation technique and downloadable relaxation audio files are provided.
5	*Relationships*	The link between thoughts, emotions and relationships is explored.<br>Problem-solving skills are provided.
–	*Review*	User completes brief outcome measures and the results of these are presented and compared with earlier results.<br>A 'Wrapping it Up' review is presented that summarises what has been learned.<br>A certificate of completion is presented.

CBT: cognitive behavioural therapy.
The structure and content of MoodGYM is updated on a periodic basis.

5.1.1.c. CHAMindWell Monitoring and Support (CHA-MW)

CAT-MH (Computer Adaptive Testing for Mental Health), based on multidimensional item response theory, adapts item presentation to the individual's severity so that different individuals are tested with different items depending on their severity level.[52] This allows for rapid testing – 2-10 minutes, depending on the number of domains tested – compared to 1-1.5 hours for a structured clinical interview, and greater precision. It also permits adaptive evaluation of complex traits, including depression, anxiety, mania and hypomania, PTSD, psychosis, suicidality, and substance abuse.[57,58] And it is more reliable for monitoring and measurement based care because it avoids the response bias of administering the same questions repeatedly as is done with standard testing methods.[58] It is easy for patients to fill in online[50] and provides a series of questions to enable the clinical researchers to ascertain whether they meet thresholds for depression, suicidality, general anxiety, PTSD, psychosis and other common and major psychiatric conditions. All of the modules provide thresholds (e.g., mild, moderate, severe, that are based on tradeoffs between sensitivity and specificity.

All groups will have CHAMindWell Monitoring and Technician support during weeks 1-8 and then every 4 weeks afterwards for 24 weeks. They will complete CAT-DI and ANX-CAT weekly and full CAT-MH every 4 weeks. Trained technicians will review scores weekly and reach out to anyone whose CAT-MH severity level increased on any scale to moderate or severe; anyone who increased by more than 10 points on CAT-DI PHQ-9 equivalency from baseline or from last CAT-DI test, anyone who misses 2 weeks of CHAMindWell CAT-DI testing in a row, or anyone who remains at a moderate level of depression for 4 or more weeks in a row. Participants with severe symptoms or worsening moderate symptoms over 2 weeks will be asked if they would like a televisit with a clinician.

<u>5.1.2. Administration:</u>

Participants will be enrolled and randomized on a rolling basis. Participants will be randomized in blocks of 5 or 10 with 2:2:1 ratio (MBCT-R + CHA-MW: iCBT + CHA-MW: CHAMindWell alone). Next, after randomization, the participant will be informed about their assignment and given a start week for group sessions within the next 2-4 weeks (either MBCT-R or iCBT) and will be given information about the intervention and its structure with a start date scheduled. We will aim to run MBCT-R groups approximately every 4 weeks, depending on demand, but will run a group at least every 8 weeks, depending on enrollment rate.

We will aim for participants to begin participating in MBCT-R or iCBT groups as soon as possible after randomization, but at least within 4 weeks. All participants will start in CHAMindWell with monthly monitoring and support prior to randomization. All participants will complete a week 0 CAT-MH during the week prior to when the MBCT-R group starts and will be informed of their intervention status that week. They will continue to receive CHAMindWell weekly monitoring for the first 8 weeks.

A research coordinator will take attendance online each week for MBCT-R, which will be transferred to REDCap by the research coordinator after the group. Engagement with iCBT curriculum will be assessed weekly by a research coordinator, which will be transferred to REDCap by the research coordinator each week.

Call logs for CHAMindWell will be stored in a confidential google sheet and all televisit notes with CHAMindWell clinicians will be recorded as televisit notes in EPIC EHR.

To more closely approximate standard care in a comparative effectiveness trial in which we are randomizing patients to one of three existing population health interventions, other non-study patients will still be able to participate in the MBCT-R groups, the iCBT program, or the CHAMindWell intervention without being required to be added to the study.

Alternatives to Participation:

Participants can still participate in any of the programs if they do not want to participate in the study. All CHA patients participating in Enhanced CHAMindWell (i.e., weekly CAT-MH interviews for 8-weeks) will still be able to receive iCBT or MBCT-R for free, regardless of their participation in this study. Participants can also be referred directly to outpatient psychiatry or primary care behavioral health providers if they would prefer this instead

5.1.3. Duration

Participants will complete study procedures for a total of 28 weeks. They will participate in MBCT-R- CHA-MW, iCBT+ CHA+MW, or weekly CHAMindWell monitoring alone for the first 8 of the 24 weeks; the remaining weeks will include assessment time points every 4 weeks.

Any CAT-MH assessment battery completed more than 2 weeks after it is expected to be completed by the study timeline would be marked as outside the acceptable assessment window and excluded from primary analyses. Any weekly CAT-DI/CAT-AD assessment completed more than 72 hours after the expected week would be marked as outside the acceptable assessment window and excluded from analyses.

5.2. Handling of Study Interventions

The MBCT-R is tailored specifically for participants with Depression, Anxiety and Stress. This manual will be sent to NCCIH prior to the start of study enrollment. The iCBT curriculum will be shared with NCCIH prior to start of study enrollment.

MBCT-R training for group leaders: Prerequisites to be eligible for training as a group leader for MBCT-R are: 1) mental health training and experience, i.e. an advanced degree in mental health (social work, psychology, psychiatry) including training in Cognitive-Behavioral Therapy, and at least two years of clinical experience in the field including experience with running groups; 2) personal experience with a regular mindfulness meditation practice; and 3) Training and experience with MBCT including attendance at the 5-day intensive foundational training and experience teaching at least one MBCT

class with supervision and mentoring by a qualified MBCT mentor. Candidates must also be thoroughly familiar with the MBCT curriculum (Mindfulness-Based Cognitive Therapy for Depression, Second Edition, Segal, Z.V., Williams, J.M.G., Teasdale, J.D., (2012) Guildford Press, New York).

MBCT-R training will start with a day-long program in a simulated classroom setting involving role plays, practice teaching, and didactics covering adaptation of the MBCT curriculum for COVID-related resilience skills. Participants will then be eligible to co-teach MBCT-R with Dr. Fulwiler, a nationally recognized MBCT mentor, who will provide mentoring and feedback. Leader trainees will be required to co-lead at least two MBCT-R classes with Dr. Fulwiler with mentoring before being eligible to lead a MBCT-R class on their own (with an additional co-leader). Trainees will be rated for both MBCT and MBCT-R teaching competence using the Mindfulness-Based Intervention Teaching Assessment Criteria (MBI:TAC)[59]. Only teachers who are rated as Competent or above will be allowed to teach MBCT-R classes for the study.

5.3. Concomitant Interventions

5.3.1. Allowed Interventions

All other non-experimental interventions are allowed as long as they do not conflict with inclusion/exclusion criteria. Many participants are also receiving standard care for their chronic illness and mental health, which may include trials of psychopharmacologic agents or psychotherapy.

5.3.2. Required Interventions

There are no additional required interventions except the home mindfulness practice encouraged in MBCT-R.

5.3.3. Prohibited Interventions

Psychopharmacologic treatments AND diagnoses related to active psychosis, thought disorder, bipolar 1 disorder, schizophrenia, or schizoaffective disorder as these would indicate that the participant meets one of the exclusion criteria. Psychopharmacologic treatments with SSRIs, SNRIs, and NDRIs are not prohibited. Stable atypical antipsychotics and mood stabilizers for the adjunctive treatment of mood disorders or bipolar 2 disorders are not prohibited as long as dose has been stable for 3 months and there is no active psychosis or mania in the past year.

5.4. Adherence Assessment

5.4.1. Measurement and Reporting of Participant Accrual, Compliance with Inclusion/Exclusion Criteria:

Review of the rate of participant accrual and compliance with inclusion/exclusion criteria will occur monthly by the Senior Research Coordinator with reports to PI monthly to

ensure that a sufficient number of participants are being enrolled. Accrual and adherence will be reported twice yearly to the DSMB. The DSMB will meet once yearly.

5.4.2. Measurement and Reporting of Participant Adherence to Treatment Protocol:
Participants will be considered adherent to the treatment protocol if they participate in at least 6 sessions of MBCT-R, 6 sessions of iCBT groups, or 6 weekly CAT-MH computerized interviews. If they are adherent to 6 intervention sessions and complete 75% of weekly surveys and 75% of CAT-MH interviews, then they will be eligible for a $20 completion bonus. Adherence for iCBT will be rated based on the level of completion of each module and adherence for MBCT-R will be rated based on the level of attendance for each session. Adherence for each intervention session will be rated on the following scale: 0 = absence; 1 = completed; 2 = incomplete/partially complete (with comments). An explanation of study staff efforts to monitor and address participant adherence deficits will be documented and reviewed twice weekly by the senior research coordinator.

5.4.3. Measurement and Reporting of MBCT-R Group Leader Adherence to Treatment Manual:
Data on adherence of MBCT-R group leaders to the MBCT-R manual will be collected weekly by research staff and reviewed monthly by the PI, and twice yearly by the study methodologist and DSMB. All MBCT-R sessions will be recorded via Zoom and saved to a secure computer, and then uploaded by a Research Coordinator or Research Assistant to CHA confidential Google Drive. We will review 10% of MBCT-R recordings by a trained reviewer rating for adherence, competence, and fidelity, using the MBI:TAC[59]. These group leader adherence reviews will be used to provide feedback to group leaders for internal quality improvement and may be reported descriptively to show that we met standards for high quality MBCT-R. If a group leader doesn't meet high quality standards, then a sensitivity analysis can be conducted with and without the data from that group, but the data itself will not be included in research.

Table 2: Schedule of Evaluation from Screen to Week 24
Table 2a: Schedule of evaluations Consent & Screening – Week 12

	Consent	Screening	Study Weeks	Baseline	Study Week											
			-4 to -0		1	2	3	4	5	6	7	8	9	10	11	12
**Informed Consent Session**																
Informed Consent Form	X															
Consent Quiz	X															
**Screening Session (and SUD outcomes)**																
CAT-MH or review if recently completed		X														
UCI Oral Health Questionnaire		X														
Tango Registration		X														
EHR Records		X														
Demographics Survey		X														
Alcohol/Substance Use History		X														
Meditation/ Mindfulness (SMME)		X														
Oral Fluid Toxicology Testing		X														
**Diaries and Saliva Sampling (OPTIONAL) *** **																
Daily Inventory of Stressors			X										X ▼			
Positive and Negative Affect Reactivity			X										X ▼			
Responses to Stress Questionnaire COVID-19 (RSQ-COVID-19)			X										X →			
IL-6 saliva** PRE			X													
IL-6 saliva** POST													X ▼			
**Baseline Battery**																

Credibility/Expectancy Qtn (CEQ)						X										
**Monitoring**																
CAT-MH* (full, includes DI/ANX)								X				X				X
CAT-DI and ANX-CAT only				X	X	X	X		X	X	X					
**Mechanisms Battery**																
Experiences Questionnaire (EQ)				X				X				X				X
Experiential Avoidance (BEAQ)				X				X				X				X
Difficulty in Emotion Regulation (DERS)				X				X				X				X
Perceived Stress Scale (PSS-14)				X				X				X				X
Self-Compassion Scale (SCS-SF)				X				X				X				X
Interoceptive Awareness (MAIA-2)				X				X				X				X
Beck Depression Inventory 2 (BDI-II)				X												X
Responses to Stress Questionnaire COVID-19 (RSQ-COVID-19)				X												X
**Covariates**																
COVID-19 Fear				X												X
COVID status self-report				X				X				X				X
Home Skills Use Diary					X	X	X	X	X	X	X	X				
Adverse Events Reporting Form				X				X				X				X
Duration (min)	30	39	24	68	7	8	7	53	7	7	7	53	24	24	28	76

CAT-MH: CAD-MDD, **CAT-DI, ANX-CAT, CAT-M/H, **CAT-SUD**, CAT-PTSD, CAT-P-SR, CAT-C-SSRS, CAT-PHQ-9 (see pg. 55 for full description)*
***saliva (also includes* TNF-a, IL-1B, IL-8)
*** Diaries and Saliva Sampling (OPTIONAL) will only be collected at a single time point for the healthy control population

Table 2b: Schedule of evaluations weeks 13-24

	Study Week												Duration (min)
	13	14	15	16	17	18	19	20	21	22	23	24	
**Informed Consent Session**													
Informed Consent Form													20
Consent Quiz													10
**Screening Session (and SUD. outcomes)**													
CAT-MH or review if recently completed													12
Oral Health Saliva Screening													4
Tango Registration													1
EHR												X	0
Demographics Survey													3
Alcohol/Substance Use History												X	5
Meditation/ Mindfulness (SMME)													5
Oral Fluid Toxicology Testing												X	5
**Diaries and Saliva Sampling (OPTIONAL)** ***													
Daily Inventory of Stressors													10
Positive and Negative Affect Reactivity													10
Responses to Stress Questionnaire COVID-19 (RSQ-COVID-19)													20
IL-6 saliva** PRE													15
IL-6 saliva** POST													15
**Baseline Battery**													

Credibility/Expectancy (CEQ)				1
**Monitoring**				
CAT-MH* (full)	X	X	X	12
CAT-DI and ANX-CAT only				2
**Mechanisms Battery**				
Experiences Questionnaire (EQ)				5
Experiential Avoidance (BEAQ)				4
Difficulty in Emotion Regulation (DERS)				8
Perceived Stress Scale (PSS-14)			X	4
Self-Compassion Scale (SCS-SF)				2
Interoceptive Awareness (MAIA-2)				5
Beck Depression Inventory 2 (BDI-II)				7
Responses to Stress Questionnaire COVID-19 (RSQ-COVID-19)			X	20
**Covariates**				
COVID-19 Fear				3
COVID status self-report	X	X	X	1
Home Skills Use Diary				5
Adverse Events Reporting Form	X	X	X	5
Total Duration (min)	20	20	64	

CAT-MH: CAD-MDD, ***CAT-DI*, ANX-CAT, CAT-M/H, ***CAT-SUD***, CAT-PTSD, CAT-P-SR, CAT-C-SSRS, CAT-PHQ-9 (see pg. 55 for full description)*
***saliva (also includes* TNF-a, IL-1B, IL-8)
^30-day TLFB added at Week 24
*** Diaries and Saliva Sampling (OPTIONAL) will only be collected at a single time point for the healthy control population

6. DESCRIPTION OF EVALUATIONS

6.1. Screening Process
Referrals to the study will come from the CHAMindWell program.

Prior to screening, participants will have completed a CAT-MH interview through the CHAMindWell population mental wellness program, which is NOT a research program. Participants with mild to moderate symptoms of depression, as quantified by a CAT-Depression Inventory (DI) score of 50-75[53], will be contacted and recruited into the main resilience study study. Participants who score ≤35 on the CAT-DI will be recruited as healthy controls for the sub-studies.

If the participant has a CAT-DI score of 50-75, then they will be considered eligible for inclusion in the main resilience study. If the participant has a CAT-DI score of ≤35 and do NOT have any other CAT-MH screening test >50, then they will be considered eligible for inclusion in the sub-studies. Next, the RC or research recruitment referral coordinator, or research assistants will contact the patient through the methods the participant has consented to be contacted in CHAMindWell (first by phone). The RC will describe the study and conduct a phone pre-screen with inclusion/exclusion criteria. If the phone is NOT answered, then they will contact the patient via MyCHArt message through EPIC or a personal email or text invitation (if the patient consented to be contacted via these methods in CHAMindWell) to talk by phone with a study coordinator about the study. The RC can help direct the patient to the enrollment and consent process if they have time and interest to do this by sending them a REDCap informed consent link by email, or by scheduling a time to do this by phone or Google Meets HIPAA-compliant video conference.

As part of screening (after consent has been obtained), the research coordinator will review the EHR record. If the screening process or EHR review suggests that the participant meets exclusion criteria or brings up other questions about eligibility, then the RC will contact the PI, Co-PI or CMC Medical Director to review the information and decide prior to proceeding to baseline surveys.

6.2. Enrollment, Final Screening, Baseline, and/or Randomization

Study enrollment is defined as the date when the online informed consent document is completed through REDCap. After enrollment a screening survey will be completed and a final clinical review screening through the EHR will be conducted. Once the patient is determined eligible from screening, they will complete their baseline measures on REDCap. They may be invited to complete an optional sub-study, including 5 daily diaries (over 7 days pre-intervention, and 7 days post-intervention) and salivary sample collection (one pre-intervention, one post-intervention). Participants who completed the main study before the optional sub-studies were available may be contacted up to 12 months post-intervention with an offering to complete a single post-intervention timepoint of 5 out of 7 daily diaries and one saliva sample. Once baseline measures are completed, then the patient is eligible for randomization, which is described below.

6.2.1. Consenting Procedure

All patients will be sent an informed consent form, which will be obtained from each participant at entry into the study via REDCap. The informed consent document will include a full description of the study procedures and review associated risks. Patients will also be asked to consent to email communication, online video recordings of sessions if applicable, and optional collection of salivary samples (if available), and optional daily diaries (if available). Participants who completed the main study before the optional sub-studies were available will be invited to complete an additional consent session that includes completion of at least 5 out of 7 daily diaries and one saliva sample at a single timepoint post-intervention. They will be asked to sign a consent addendum via REDCap that acknowledges their willingness to join the sub-studies. Because these interventions are well-established with more than 1000 studies demonstrating the safety and efficacy of Mindfulness-based interventions[60] and CBT interventions, the interventions represent a low-risk experience. Strict confidentiality during the consenting procedure will be maintained through use of a secure online REDCap platform, and the study staff will be trained on maintaining confidentiality during the consent process. The research coordinator will call or video chat with the patient as they proceed through the online consent form reviewing the main points together. If the participant has questions, then the coordinator will describe the key information of the study verbally, using an outline designed for easy patient comprehension, and will then give the patient time to review the consent form on their own. The patient will be given a chance to ask questions and express any concerns about the study. If the patient has questions for a clinical provider, then a mental health clinician on the study team will be scheduled to speak with the patient prior to enrollment. No study-specific procedures or investigations will be performed before the patient has electronically signed and dated the Informed Consent Form. After the informed consent session, the research coordinator will document that informed consent was monitored and completed in REDCap.

6.2.2. Consent Assessment:

Prior to signing the informed consent, the participant will be asked to complete an Informed Consent Assessment (Consent quiz) through REDCap to ensure comprehension of the study procedures, rights of participants, as well as understanding of the risks and benefits of the study. The participant will not be able to proceed with the study without having at least 90% correct answers on the consent assessment.

Participants will be given the opportunity to review the study procedures with a trained study staff member if they fail the consent assessment. Participants will also be given an opportunity to correct their answers on the consent assessment. If they can't pass the assessment with at least 90% completely correct answers after reviewing it and the protocol with the coordinator, then they will NOT be able to proceed to signing the informed consent and they will need to be evaluated for the presence of reading difficulties or cognitive deficits disrupting their ability to complete study tasks prior to re-consideration in the study. Once the participant demonstrates understanding of the study by passing the consent assessment, the consent will be electronically signed and dated

and they will then be considered enrolled in the study. Participants who have been invited back to the study to complete a single post-intervention timepoint of daily diaries and saliva sample will complete an informed consent addendum that does not include the comprehension quiz they completed upon initial consent. The signed informed consent forms, and addenda, will be stored electronically and the participant will be instructed to download a copy of the signed informed consent form for their own records.

6.2.3. Participant Coding

Upon consenting to the study, each participant will be assigned a coded study number by the research coordinator based on their order of enrollment and a unique study acrostic which is based on letters (to allow for cross-referencing and prevent mistaken confusion among participants if a digit is incorrect).

6.2.4. Salivary Screening Assessments

Following consent, the research coordinator will schedule a time to conduct oral fluid toxicology screening via videochat.

6.2.4.a. Screening Oral Fluid Toxicology (Salivary) Testing (REQUIRED)

All participants will be shipped a package containing sponge-based testing kits for Oral Fluid Toxicology testing. Oral fluid toxicology tests will be conducted via videochat with a research coordinator to ensure proper collection. The participant will hold the results panel of the test up to their camera for the research coordinator to screenshot using HIPAA-compliant technology, such as Droplr (https://droplr.com/), to securely upload screenshots directly to the secure study Google Drive. Participant faces and other PHI will not be included in the screenshot.

6.2.4.b. COVID-19 Salivary Testing (OPTIONAL)

Sub-study participants will come to the Center for Mindfulness and Compassion (CMC), once at baseline prior to the study intervention and once after the intervention so that a trained study research coordinator or research assistant can collect a saliva sample. Participants that opted into the sub-study after completing the main study will only come to the CMC once post-intervention for a single saliva sample collection. Participants that consented into the sub-study as healthy controls will only come to the CMC once for a single saliva sample collection.

The COVID-19 antibody saliva samples will be collected at the same time as the IL-6 cytokine passive drool saliva samples (described in section 6.2.5.b. below). Samples will be stored in a locked CMC freezer by research coordinators prior to shipment to Salimetrics for analysis.

6.2.5. Baseline Assessments

Eligible participants may complete baseline daily tracking and will complete baseline surveys through a link to the secure REDCap database using their personal devices.

6.2.5.a. Optional Baseline Daily Tracking (Weeks -4 - 0):
If asked to participate in this optional sub-study, eligible participants will first be asked to complete at least 5 daily evening diary surveys. These evening surveys will assess daily stress and stressful events related to COVID-19 from the past day. In addition, experiences of negative and positive affective states during the past day will also be assessed based on method by Almeida lab[24]. Participants will have a total of 7 days to complete daily diaries; however only 5 submissions are required. Participants are encouraged to complete as many daily diaries as they so choose. This will ensure ample time for participants to complete the baseline daily tracking. Upon completion of baseline daily tracking, participants will be sent the baseline survey battery. Participants not enrolled in the optional baseline daily diaries will be sent the general baseline survey battery following completion of screening assessments. Participants will be asked to complete at least 5 daily diaries (over the course of 7 days) again during weeks 9-12, or up to 12 months following intervention completion for those who join after completing the main study.

6.2.5.b. Optional IL-6 Cytokine Saliva Collection (Weeks -4 - 0):
If asked to participate in this optional sub-study, all sub-study participants will be screened after enrollment for oral infection, oral hygiene behaviors, smoking status, and alcohol consumption. Since alcohol and smoking may impact salivary IL-6 levels, status will be included as covariate in IL-6 analyses. Additionally, time since stressor will be included as a covariate in IL-6 analysis. Both on a day prior to the study intervention and on a day following the intervention, participants in the salivary sub-study will be asked to come to the CMC to collect a saliva sample (ideally between days 2-6 of the daily diary tracking so that they can have a daily diary for the day before, day of sample, and day after sample). They will be encouraged to complete these within the 7 consecutive daily diary days. Participants who join this optional sub-study after they completed the main study will only provide a saliva sample once post-intervention. Participants who join this sub-study as healthy controls will only provide a saliva sample once, and will not complete any intervention as part of the study. After collection, all samples will be stored in a locked CMC freezer until week 12 (and can be stored frozen at the CMC up until 24 weeks from collection date), as recommended by Salimetrics. Research Coordinators will ship both pre-intervention and post-intervention saliva samples to Salimetrics sometime after week 12 and before 24 weeks after the collection date.

6.2.5.c. Consent, Screening and Baseline Survey Batteries (Week -4 and Week 0):

The Consent and Screening Survey Battery will take 32 minutes and include the following:

- Informed Consent Form
- Consent Quiz
- Review and Confirmation of most recent CAT-MH for inclusion/exclusion criteria
- UCI Oral Health Questionnaire

- Tango Registration
- Demographics Survey
- PhenX Alcohol/Substance Use History (Past 30-day, lifetime)
- Meditation and Mindfulness Experience (SMME)

The Baseline Survey Battery consists of the following and is expected to take approximately 60 minutes.

- Baseline CAT-MH
- Experiential Avoidance (BEAQ)
- Difficulty in Emotion Regulation (DERS)
- Perceived Stress Scale (PSS-14)
- Self-Compassion Scale (SCS-SF)
- Interoceptive Awareness (MAIA-2)
- Experiences Questionnaire (EQ)
- Beck Depression Inventory 2 (BDI-II)
- Responses to Stress Questionnaire COVID-19 (RSQ-COVID-19)
- Alcohol and Drug Timeline Follow-back (TLFB)
- COVID-19 Fear
- COVID Status Self-Report
- Ranked Desire for each intervention option (RD)

In addition, a trained research coordinator will review the patients' EHR and review their oral fluid toxicology data, clinical diagnosis, and medication doses.

6.2.6. Randomization and Allotment

We will randomize participants after they complete the baseline survey battery. Participants will be randomized in blocks of 5 or 10 with a 2:2:1 ratio of MBCT-R: iCBT : CHA-MW alone. Randomization lists will be provided to the study senior RC with the randomization of study numbers by the methodologist using STATA random generator. When a participant is eligible, the research coordinators will inform the senior RC, data analyst, or the methodologist who will then provide the randomization status. A research coordinator will then call the participant by phone to let them know the date/time that they can start the MBCT-R, the date and process for starting the iCBT group, or the process for ongoing CHAMindWell CAT-MH surveys.

6.2.7. Blinding

The Data Analyst, Senior RC, and methodologist will be blinded to participant identity prior to and during randomization. The research coordinators will not have access to the randomization spreadsheet. See Table 3 for blinding status for study staff. Participants will not be blinded to their assigned study arm (MBCT-R, iCBT, vs. CHA-MW alone), nor will the Co-PI, research coordinators, data analyst, or methodologists. The PI will be blinded to the assigned study arm when reviewing data quality, adverse events, protocol violations, etc. The PSU study team will be blinded to which arm is MBCT-R or iCBT in their analysis of exploratory sub-study daily diaries and salivary cytokine data.

Table 3. Blinding Status of Study Staff

Study Role	Blinding Status	Time of unblinding
PI	Blinded	When all primary outcome data have been collected and when database is locked
Co-PI	Not blinded	
Senior RC	Not blinded	Unblinded after randomization and allocation
Data Analyst	Not blinded	Will prepare reports for DSMB with unblinded data, but will not have contact with participants.
Methodologists	Not blinded	Will review reports for DSMB with unblinded data, but will not have contact with participants.
RCs	Not blinded	
Clinical Co-Is and Medical Directors	Not blinded	
Participants	Not blinded	

6.3. Overview of Study Assessments

Study assessments are outlined below:

- Phone Pre-Screening (0.25 hours)
- Consent and Screening Session (0.75 hours)
- Oral Fluid Toxicology Screening and Optional Sub-Study Training Session (0.5 hours)
- Optional Sub-Study Baseline Salivary Sample Collection (0.25 hours)
- Optional Baseline Daily Tracking (~1 hour) (10 min daily survey x 5 days)
- Baseline Survey Battery (1 hour)
- Weekly Surveys (Weeks 1, 2, 3, 5, 6, 7, 8 10 min)
- 4-week Assessment (0.5 hours)
- 8-week Assessment (0.5 hours)
- 12-week Assessment (1.25 hours)
- Optional Week 9-12 Daily Tracking (~1 hour) (10 min daily survey x 5 days)
- Optional Sub-Study Week 9-12 Salivary Sample Collection (0.25 hours)
- 16-week Assessment (0.5 hours)
- 20-week Assessment (0.5 hours)

- 24-week Assessment (0.8 hours)

6.4. Participant Communication

Study research coordinators will contact patients via phone calls throughout the course of the 24-week study if (a) their CAT-MH interview triggers a suicidality alert, or (b) they have missed a MBCT-R group session or did not participate in any iCBT within past week, or (c) if they fail to complete a CAT-MH survey. If a participant does not respond to the phone call and has not been responsive within the past two weeks, study staff will reach out using e-mail or text message. Anyone who increased by more than 10 points on CAT-DI from baseline or from last CAT-DI test, anyone who misses 2 weeks of CHA CAT-DI testing in a row, or anyone who remains at a moderate level of depression for 4 or more weeks in a row will receive a call and will be asked if they would like a televisit with a clinician. Participants with severe symptoms or worsening moderate symptoms over 2 weeks will be asked if they would like a televisit with a clinician.

The voicemail, text, and email outreach attempts will be limited to scheduling and general content and will not include any mention of the research study or the nature of the study. Participants will be contacted if they miss surveys or group visits. In these cases, the research coordinators will reach out to participants and schedule a televisit with a clinician if needed. Participants who completed the main study before the sub-studies were available may also be contacted up to 12 months following the intervention with an invitation to re-consent to complete a single timepoint of 5 out of 7 daily diaries and one saliva sample.

Only secure WiFi will be used to communicate with participants. Only a CHA IT approved secure study cell phone and computer, both password-secured, will be used to communicate with participants. However, in the event of a COVID-19 related lockdown, research coordinators will use their personal devices to access the CHA-secure network or CHA-approved remote communication process (e.g. CHA's approved calling system) to communicate with participants. All communication will be logged in a G-suite secure study call log, and messages will be deleted from the study cell phone or social media site once logged in the call log.

6.5. Completion/Final Evaluation

Final primary outcome/endpoint is reduction in depression severity as demonstrated by CAT-MH during 24 weeks with primary time points at 4, 8, 12, 16, 20 and 24 weeks using a joint test.

Final study session is week 8, participants will continue to complete CAT-MH assessments in 4-week periods for a total of 16-weeks following completion of the intervention. Please refer to the schedule of evaluations for an exact list of assessments used in this session.

6.6. Participant Reimbursement:

Participants will be paid using either anonymous, retail, online reward cards, OR anonymous, HITECH safe reloadable prepaid cards. Specifically, these online reward cards would be provided through a company like Tango (https://www.tangocard.com), a HIPAA compliant rewards service, or Comdata, a CHA approved reloadable gift card vendor. Use of Tango will require the input of participant email. Use of anonymous, retail, non-reloadable prepaid cards will not require any participant information to be recorded.

Participants will also be rewarded with a completion bonus for more than 75% completion: including attendance to 6 group MBCT-R sessions or 6 iCBT sessions completed, completion of at least 6 of 8 weekly CAT-MH and skills use surveys during first 8 weeks. They will also need to complete 75% of CAT-MH full interviews at baseline, 4-week, 8-week, 12-week, 16-week, 20-week, and 24-week assessments, and more than 75% of weekly CAT-MH and skills use surveys.

Payment will be given as follows:

- $20 at Screening/Consent for completion of screening, informed consent, and oral fluid toxicology screening
- $20 for completion of Baseline Assessment
- $60 after 12 Week Assessment (including 4 wk + CAT-MH ($20), 8 wk + CAT-MH ($20), 12 wk + CAT-MH ($20)
- $40 at 24 Week Assessment (incl. 16-wk CAT-MH ($10), 20wk CAT-MH ($10), and 24 wk + CAT-MH ($20))
- Up to $20 completion bonus including attendance to 6 group MBCT-R sessions or 6 iCBT sessions completed (this criterion does not apply to participants in the CHAMindWell monitoring study arm), completion of at least 75% (6 of 8) weekly CAT-DI and skills use surveys during first 8 weeks. They will also need to complete 75% of CAT-MH full interviews at 4-week, 8-week, 12-week, 16-week, 20-week, and 24-week assessments.
- $40 for collection of 2 saliva samples ($20 per sample) (for those enrolled in optional sub-study) [Participants who provide a single post-intervention saliva sample will receive $20].
- $40 for completion of all daily diaries ($20 for completion of all 5 baseline daily diaries and $20 for completion of all 5 post-intervention daily diaries) (for those enrolled in optional sub-study) [Participants who complete at least 5 out of 7 daily diaries only post-intervention will receive $20].
- [If applicable] $20 at Screening/Consent for completion of screening and informed consent for the optional sub-studies after having completed the main study.

Total study payments possible will be $160 dollars in gift cards or pre-paid cards.$20-40 will be available to participants who participate in the saliva sub-study and another $20-40 available to participants who participate in the daily diaries sub-study. Participants who join the sub-studies after having completed the main study will receive an additional

$20 for screening and consent. Participants will be paid $140 for completing all main study assessments and CAT-MH interviews with a completion bonus of $20 for greater than 75% completion as described above. Participants who join the sub-studies as healthy controls may be paid $20-$60 for screening/consent completion, 1 saliva sample, and at least 5 out of 7 daily diaries completed.

Study Payments will be given the following times:
Payment 1: $20 for Screening/Consent visit
Payment 2: $20 after baseline surveys
Payment 3: $60 after 12 Week Assessment
Payment 4: $40 after 24 Week Assessment
Payment 5: $20 for completion bonus
Payment 6: $20-40 for saliva collection (optional sub-study)
Payment 7: $20-40 for pre- and/or post- daily diaries (optional sub-study)
Payment 8: $20 for Screening/Consent following main study completion (if applicable)

7. SAFETY ASSESSMENTS

AEs will be systematically assessed at each assessment time point (monthly assessments). In addition, group leaders and research coordinators will be trained to identify and report any adverse events that may occur or are reported during assessments every 4 weeks. All AEs will be reviewed weekly by an RC and the Co-PI or CMC Medical Director, and monthly by the PI, and SAEs will be reviewed by PI within 24 hours. Please see adverse events section (7.3) below.

7.1. Specification of Safety Parameters

Participants are assessed regularly for AEs, hospitalization, and changes in medication.

7.2. Methods and Timing for Assessing, Recording, and Analyzing Safety Parameters

<u>7.2.1. Expected Risks</u>

7.2.1.a. MBCT-R

Research coordinators and group leaders will be trained to respond to escalating anxiety, psychosis, or any other worsening psychiatric symptoms. They will be trained to alert a clinician within 24 hours of becoming aware of escalating symptoms, and to contact the CMC Medical Director, Co-PI, or Principal Investigator by phone or page as soon as possible and at most within 24 hours of alerting the existing care provider (s) to review the case. The care team and study staff will work together to determine the appropriate

referral, including referral to outpatient mental health and/or local Emergency Department if necessary.

1. Time spent learning mindfulness techniques

Participants will be invited to practice mindfulness skills and techniques at home, which may involve negotiating with those at home to create time and space for practice. This will not be required of them.

2. Worsening of underlying mental health symptoms or increased anxiety due to difficulties in mental training program

Some people may have continued worsening of their mental health symptoms. Mindfulness practices can sometimes cause anxiety for those who practice. Support from the Group Leader and the group can help participants recognize that this anxiety is part of the process and can help people learn to manage these feelings. If meditation and mental exercises are causing a worsening of anxiety for participants, they will be encouraged within groups to change their approach to the practice. If the participant(s) are unable to find a way to practice without eliciting an increase in symptoms, then they may be asked to stop practicing and to meet with either the Co-PI, the CMC Medical Director or the Principal Investigator for an evaluation. The participant may ultimately be terminated from the study if they are experiencing an adverse reaction. These participants may continue to receive other treatments as part of their ongoing treatment plan.

3. In some very rare cases, meditation practice can lead to a dissociative state or to psychosis[61].

This is more likely in participants with current conditions of or a predisposition to psychosis. Participants experiencing active psychosis are excluded from participating in this study. If a participant begins to experience these feelings during his or her time in the study, then the study staff and care team will work together to alert the appropriate care team providers and study investigators immediately. The participant may be asked to stop practicing and to meet with either the Co-PI, CMC Medical Director or the Principal Investigator for an evaluation in person or via videoconference. Videoconferencing will occur through the HIPAA compliant and encrypted communications platform Zoom or Google Meets. The participant may ultimately be terminated from the study if he or she is experiencing an adverse reaction. These participants may continue to receive other treatments as part of their ongoing treatment plan.

4. Physical discomfort due to gentle movement aspect of training.

The training will involve gentle movements and stretching. Some participants could experience physical discomfort during this aspect of training. If a participant feels discomfort, he or she will be encouraged to engage in a less straining manner. If discomfort continues, then the participant will be asked to sit out of this brief element of

training and discuss the areas of discomfort with their PCP before return to practice of physical postures. Severe discomfort is not expected, but could possibly occur. In this case, the participant will be treated for this pain, and encouraged to return to practice stationary forms of mindfulness. If a participant gets hurt or gets sick as a direct result of being in this study, emergency treatment will be given to them. All needed emergency care is available to participants, just as it is to the general public. Cambridge Health Alliance has not set aside any money to pay for a research-related injury or illness.

5. Feelings of embarrassment or anxiety when asked personal survey questions.

Some survey questions are of a sensitive or personal nature and may cause the participant to become upset. In some rare cases, participants may require mental health support upon feeling upset by the survey questions. A participant's referring clinician will be contacted if the participant needs additional mental health support. The PI, Co-PI, and CMC medical director will remain available to field questions from participants about their experience with mindfulness and any adverse experiences. The clinical director will help to monitor participants and coordinate behavioral health care for participants.

6. Despite efforts to prevent data breaches, any use of electronic devices and internet data transmission can result in a **breach of confidentiality**.

7. Group members will be asked to keep what others share confidential, but they may not. As a result, **some private information may be disclosed** by other group members.

7.2.1.b. iCBT

1. Despite efforts to prevent data breaches, any use of electronic devices and internet data transmission can result in a **breach of confidentiality**.

2. **Worsening of underlying mental health symptoms or increased anxiety** due to difficulties in mental training program

CBT practices can sometimes cause anxiety for those who practice. If the participant(s) are unable to find a way to continue iCBT without eliciting an increase in symptoms, then they may be asked to stop practicing and to meet with either the Co-PI, the CMC Medical Director or the Principal Investigator for an evaluation. The participant may ultimately be terminated from the study if they are experiencing an adverse reaction. These participants may continue to receive other treatments as part of their ongoing treatment plan.

7.2.1.c. CHAMindWell Monitoring

1. Despite efforts to prevent data breaches, any use of electronic devices and internet data transmission can result in a **breach of confidentiality**.

2. Monitoring may lead to anxiety and difficulty with completion of interviews may lead to feelings of guilt or shame. Staff will specifically be trained not to shame anyone about missed sessions, but instead focus on what they can do to complete the next ones.

7.2.1.d. Optional Salivary Monitoring

1. Increased awareness of oral health problems.

7.2.2. Expected Benefits

1. Participants may learn about others who have similar problems as they do, helping them feel less alone.
2. Participants may feel increased accountability in their mental wellbeing by being in group and engaging in monthly monitoring questionnaires.
3. Participants may find that they smoke fewer cigarettes and drink less alcohol.
4. Participants can learn skills for controlling behavior and improved well-being.
5. Participants may feel less depression, anxiety, stress, and pain.
6. Participants may benefit from the support of group-based treatment.
7. Participants may feel more joy and gratitude.
8. Participants receive access to proper level of care.

7.3. Adverse Events and Serious Adverse Events

Following NCCIH request, a detailed Data and Safety Monitoring Plan (DSMP) was designed and a DSMB assigned. Please see appendix at the end of this document. Adverse event reporting guidelines are detailed in the DSMP and copied below.

<u>7.3.1. Definition:</u>
In this study we will use the FDA definition of adverse events (AEs): "any untoward medical occurrence that may present itself during treatment or administration with a pharmaceutical product [or group-based treatment intervention], and which may or may not have a causal relationship with the treatment." Serious adverse events for this trial will be defined as any AE temporally associated with the participants' involvement in research that meets any of the following criteria:

- Death
- A life-threatening event
- Inpatient hospitalization
- A persistent or significant disability/incapacity
- A congenital anomaly or birth defect
- An important medical event based upon appropriate medical judgment.

<u>7.3.2. Classification of AE Severity:</u>
AEs will be labeled according to severity, which is based on their impact on the patient. An AE will be termed "mild" if it does not have a major impact on the patient, "moderate" if it causes the patient some minor inconvenience, and "severe" if it causes a

substantial disruption to the patient's well-being. Of note, a severe AE and a serious adverse event (SAE) are distinct terms. A subject could experience a severe AE that does not meet the above-listed definition of an SAE; alternatively, a subject could experience a moderate AE that meets the SAE definition.

7.3.4. AE Attribution Scale:

AEs will be categorized according to the likelihood that they are related to the study intervention. Specifically, they will be labeled unrelated, possibly related, or definitely related to the study intervention.

7.4. Reporting Procedures

This study will comply with the reporting requirements from the Cambridge Health Alliance IRB. The PI will report to the CHA IRB, DSMB, and NCCIH any of the following *unanticipated problems* and *adverse events* that occur 1) during the conduct of the study, 2) after study completion, or 3) after participant withdrawal or completion:

1. *Internal adverse events* that are *unexpected,* and related or *possibly related to the research* and that indicate there are new or increased risks to participants;
2. *External adverse events* that are *serious*, *unexpected*, and related or *possibly related to the research* and that indicate there are new or increased risks to participants that require some action (e.g., modification of the protocol, consent process, or informing participants);
3. *Deviation* from the approved research protocol or plan without IRB approval in order to eliminate apparent immediate hazard to participants or harm to others;
4. *Deviation* from the approved research protocol or plan that placed participants or others at an increased risk of harm regardless of whether there was actual harm to participants or others;
5. Any event that requires prompt reporting according to the research protocol or investigational plan or the sponsor;
6. Breach of confidentiality or violation of HIPAA
7. Procedural error regardless of whether participants experienced any harm;
8. Interim analysis, safety monitoring report, publication in a peer-reviewed journal, or other finding that indicates that there are new or increased risks to participants or others or that participants are less likely to receive any direct benefits from the research;
9. Complaint by/on behalf of a research participant that indicates that the rights, welfare, or safety of the participant have been adversely affected or that cannot be resolved by the investigator;
10. Incarceration of a research participant during participation in this study (which is not currently approved for involvement of *prisoners* as participants);
11. *Noncompliance* with applicable regulations or requirements or determinations of the IRB identified by the research team or others that indicates that the rights, welfare, or safety of participants have been adversely affected;
12. *Suspension* or *termination* of the research, in whole or in part, based on information that indicates that the research places participants at an increased risk of harm than previously known or recognized;
13. Suspension or disqualification of an investigator by the sponsor, or others;

14. *Scientific misconduct*; or

15. Any other problem that indicates that the research places participants or others at an increased risk of harm or otherwise adversely affect the rights, welfare or safety of participants or others.

7.4.1. Procedures for Collecting and Reporting Unanticipated Problems:

All unanticipated problems (including AEs) will be collected by the PI or other study staff on an AE Tracking Log form, at the following time points: weeks 0, 4, 8, 12, 16, 24, and additionally on an ad-hoc basis.

Reports of *unanticipated problems involving risks to participants or others* will be submitted to the IRB, DSMB and NCCIH within 5 working days of the date the investigator first becomes aware of the problem.

7.4.2. Reporting Unanticipated Problems that are Adverse Events:

Any unanticipated untoward or unfavorable medical occurrence, including abnormal sign, symptom, or disease, that indicates that the research places participants at increased risk of physical or psychological harm than previously known or recognized will be submitted as an AE to the IRB, DSMB, and NCCIH. The PI will provide the following information in the report:

1. A detailed description of the adverse event;
2. The basis for determining that the event is unexpected in nature, severity, or frequency;
3. The basis for determining that the event is related or possibly related to the research procedures;
4. The basis for determining that the research places participants at an increased risk of harm (i.e., a serious adverse event); and
5. Whether any changes to the research or other corrective actions are warranted.

7.4.3. Reporting Unanticipated Problems that are not Adverse Events:

All other unanticipated problems incidents, experiences, information, outcomes, or other problems that indicate that the research places participants at an increased risk of physical, psychological, economic, legal, or social harm than was previously known or recognized will be submitted as an "Other Event" to the IRB, DSMB, and NCCIH within 5 business days. The investigator will provide the following information in the report:

1. Detailed description of the unanticipated problem;
2. The basis for determining that the problem is unexpected;
3. The basis for determining that the problem indicates that the research places participants at an increased risk of harm; and
4. Whether any changes to the research or other corrective action are warranted.

7.4.4. SAE Reporting:

SAEs that are unexpected, serious, and possibly related to the study intervention will be reported to the IRB, DSMB, and NCCIH in accordance with requirements. Any SAE, whether related to study intervention or not, will be reported to the IRB and the DSMB within 5 business days. The initial SAE report will be followed by submission of a completed SAE report. SAEs that are unanticipated, serious, and possibly related to the study intervention will be reported to the NCCIH within 5 business days. Anticipated or unrelated SAEs will be handled in a less urgent manner but will be reported to the IRB in accordance with their requirements. A summary of the SAEs that occurred during the previous year will be included in the annual progress report to NCCIH. In the annual AE summary, the DSMB Report will state that they have reviewed all AE and SAE reports.

7.5. Follow-up for Adverse Events

Communication of Adverse Events:

The PI and the Co-PI will be notified of all adverse events that occur within 48 hours of it being reported to the study team. The PI will then decide what appropriate actions to take in order to ensure participant safety. In some cases, the participant may have already contacted their relevant providers, and/or the participant has already improved in which case no action is necessary.

Once the necessary steps to ensure participant safety have been enacted, the CMC medical director, Co-PI, and/or PI will discuss what actions need to be taken with regard to the study: discontinuing the participant from the intervention temporarily until conditions have improved; discontinuing from the intervention permanently but keeping them in the study; or having them drop out of the study entirely.

7.6. Safety Monitoring

The PI and data analyst will review with the research coordinators data collection, data completeness and accuracy as well as protocol compliance on a monthly basis. Study progress and safety will be reviewed monthly (and more frequently if needed). Progress reports, including participant recruitment, retention/attrition, and AEs, will be provided to the DSMB every 6 months (see DSMP for details).

Possible Risk of Suicide:

During prescreening, study applicants who have not completed a CAT-MH in past 28 days will undergo a CAT-MH computerized interview to determine their eligibility and safety for their participation in this study. Special attention will be placed on the severity of the CAT-Depression Inventory as well as suicidality and psychosis modules. Applicants who are actively suicidal will not receive the study interventions and will be contacted immediately by one of the study clinicians to assess safety and possible need for emergency treatment. The CAT-MH interviews will include a CAT-SI section that focuses on suicide risk as well as the Columbia Suicide Scale.[62] Any patients with severe suicide risk or imminent harm on CSS will cause CAT-MH to send an urgent email to both RCs, CMC Medical Director, Co-PI and PI who will then follow-up on it within 12 hours.

If imminent risk of suicide or danger to self or others is evidenced during the prescreening evaluation, then the participant will be excluded from participation and will be referred to the emergency room or the appropriate level of care as soon as possible. The PI and Co-PI will be responsible for overseeing risk assessment during screening and study assessments. The PI or CO-PI will be contacted by page and text as soon as possible to help with clinical management. If the PI is unavailable, Todd Griswold (CMC Medical Director), or Carl Fulwiler (Co-PI) can be consulted to cover.

If a participant describes feelings of suicidality while participating in a group, the licensed mental care provider present during the group will help to manage this event with the current standard of care and will make immediate contact with the PI, Zev Schuman-Olivier, or clinical coverage listed above. The participant will be referred to the emergency room or the appropriate level of care. The PI will be notified within 24 hours of any suicidality.

8. INTERVENTION DISCONTINUATION

8.1. Participant Withdrawal/Termination Criteria:

Withdrawal/Termination criteria for Participants:

Participants may be discontinued from just the study intervention treatment ("Group Discontinuation") OR both the study intervention treatment and study assessments ("Study Withdrawal") at any time.

Specific reasons for discontinuing a participant ("Group Discontinuation") from the group are:

1. Voluntary discontinuation by the participant who is at any time free to discontinue his or her participation in the group, without prejudice to further treatment.
2. Safety reasons as judged by the investigator.
3. Substance use disorder or psychiatric symptoms worsen to a level that requires a higher level of care.
4. Revealing private information about other participants in the groups.

Specific reasons for withdrawing a participant ("Study Withdrawal") from the entire study are:

1. Voluntary discontinuation by the participant who is at any time free to discontinue his or her participation in the study, without prejudice to further treatment.
2. Safety reasons as judged by the investigator.
3. Incorrect enrollment (i.e., the participant does not meet the required inclusion/exclusion criteria) of the participant.
4. Active severe substance use disorder or level of intoxication during study procedures that precludes ability to conduct assessments.
5. Participants unable to complete baseline assessments.

8.2. Withdrawing/Group Discontinuing procedures

Participants who discontinue will be asked about the reason(s) for their discontinuation and the presence of any adverse events. If possible, they will be seen and assessed by the Principal Investigator. Serious and Unexpected Adverse events will be followed up.

If participants are terminated from group treatment for any reason (except incorrect enrollment or safety concerns that endanger the study team), then they will still be expected to complete their outcome assessments, unless the participant revokes informed consent and withdraws from study.

Safety precautions for discontinuing a participant will be followed. Participants will be informed about discontinuation from group by the clinician leading the group and alternative treatment options will be discussed with the participant. If the participant is not available to receive this communication, then a letter will be sent informing the participant about the group discontinuation and providing alternative treatment options.

If a participant is withdrawn from the study, they will be contacted by the Principal Investigator and informed the reason they are being withdrawn. A Withdrawal Letter will be sent informing the participant about the study withdrawal. If the participant was still in a treatment group, then the group discontinuation process will also be completed as described above with attention to providing alternative treatment options.
Participation in this research study is voluntary. Participants can choose not to participate, and their decision will in no way affect the quality of care they receive at CHA or their working relationship with CHA. Participants may elect at any point to discontinue their participation in this study.

Participants who become pregnant during the study will not be discontinued. If a participant becomes pregnant during the study, then the study team will alert the referring provider about the pregnancy and encourage the provider to follow their standard of care. Mindfulness groups during pregnancy have been shown in meta-analysis to reliably reduce anxiety, depression, and stress[43].

8.3. New Information

Any pertinent new information will be communicated to study participants via e-mail if they have an e-mail address, otherwise via phone, by the site research coordinator. In light of new information, participants may elect to discontinue their participation in the study.

9. STATISTICAL CONSIDERATIONS

9.1. General Design Issues

Retention can be a problem in online behavioral group programs. To support the attractiveness of the study and ensure outcome measure completion, we will use a debit card with contingency bonus for survey completion. We will also use a study completion

bonus to encourage participation in all study assessment sessions, surveys and diaries. We will email all participants who missed a session within 48 hours. If a participant misses a survey, or more than one session then we will contact them. If participants continue to be unresponsive after 4 calls within 72 hours, then we will send a message to their provider to ensure that they continue to receive the appropriate level of care.

9.2. Sample Size and Randomization

9.2.1. Justification of Sample Size and Power Calculations:

This study will enroll a maximum of n = 240 participants and randomize 200 participants (80 MBCT-R: 80 iCBT: 40 CHAMindWell alone) over 12 months. Our power analyses have identified that we will have the sample to identify meaningful effects at N= 160, assuming 20% attrition by 24 weeks.

In previous MBCT studies, MBCT has an impact over 24 weeks on reductions in depression at an effect size (ES) of d = -.47[36]. iCBT Mood Gym has an effect size of g = -0.36 in metanalyses compared with control[54]. In meta-analyses of online CBT, online interventions with monitoring and coaching have and ES of d = -0.58 with admin support, d = -0.78 with professional clinical support, but d = -0.38 without any coaching or support[563]. Based on these studies, and since CHAMindWell involves monitoring with adaptive admin coaching and referral to professional support, we anticipate CHAMindWell alone (Arm 3) will have an ES of around d = -0.4, iCBT(Mood Gym) + CHAMindWell (Arm 2) will have within-group ES of d = -0.78 and MBCT-R + CHAMindWell will have within-group ES of d = -0.87. Overall, we expect MBCT-R + CHAMindWell (Arm 1) versus CHAMindWell monitoring alone (Arm 3) to have between-group ES of d = -0.47.

Participation of 200 individuals randomized 2:2:1 to intervention and comparator arms allows for 80% power to detect a net effect size of d= -0.44 for decreases in depression symptoms/severity when comparing baseline to 24-weeks changes among Arm 1: MBCT-R + CHAMindWell vs. Arm 2: iCBT (Mood Gym) + CHAMindWell to Arm 3: CHAMindWell alone. This power calculation was performed with the powerlmm package in R[64], assuming statistical analysis using linear mixed effects models with group and time main effects, group-by-time interactions, and person-specific random intercepts. As the primary outcome will be analyzed in an intent-to-treat analysis, it is not sensitive to the number of individuals who drop out of the study after randomization. With a final sample of 160 completers at 24 weeks after 20% dropout, we expect to have 80% power to detect an effect size of d=0.47 on secondary outcomes.

9.2.2. Treatment Assignment Procedures

Participants will be randomized in a 2:2:1 allocation to MBCT-R: iCBT: CHAMindWell alone in randomized block sizes of 5[65].

9.3. Definition of Populations

Intent-To-Treat analyses will be performed according to the following definition of the intent to treat population: All individuals who are randomized in the study, which is defined as those individuals who complete informed consent, meet inclusion criteria and do not meet exclusion criteria, complete all baseline assessments, are randomized and receive information about their randomization status.

9.4. Interim Analyses and Stopping Rules

This study will be stopped prior to its completion if: (1) the intervention is associated with adverse effects that call into question the safety of the intervention; (2) any new information becomes available during the trial that necessitates stopping the trial; or (3) other situations occur that might warrant stopping the trial. If the DSMB feels that a safety review to consider stopping the trial is required, then a safety review will be scheduled with the PI, and the IRB and sponsor will be informed. While no formal interim analyses are planned, we do plan to provide regular data monitoring for consistency and to decrease missing values. During these checks, we will monitor the data for unusual patterns that might suggest issues related to safety or adverse effects.

9.5. Outcomes

9.5.1. Primary Outcomes

Change in depression symptom severity as measured by CAT-MH Depression Inventory (DI) scores at baseline and during 24 weeks after randomization (week 0,4, 8, 12, 16, 20, and 24) [50,58].

9.5.2. Secondary Outcomes

1. Rates of positive toxicology screens for alcohol or unprescribed substances at 24 weeks
2. Levels of substance use (lower number of heavy drinking OR drug use days per month) on 30-day TLFB at 24 weeks
3. Number of mental health clinician televisits required during first 12 weeks
4. Number of mental health clinician televisits required during 24 weeks.

9.5.3. Exploratory Outcomes for Optional Sub-Study

1. Mean level of negative affect on stressor days compared with non-stressor days post-intervention compared to pre-intervention.
2. Mean level of positive affect on stressor days compared with non-stressor days post-intervention compared to pre-intervention.
3. Difference between mean level of salivary Interleukin-6 pre-intervention and post-intervention.
4. Associations between daily diary variables and Interleukin-6 level.

Mechanistic measures

1. Brief Experiential Avoidance Questionnaire (BEAQ)
2. Difficulties in Emotion Regulation Scale (DERS)
3. Perceived Stress Scale (PSS-14)
4. Self-Compassion Scale Short Form (SCS-SF)
5. Multidimensional Assessment of Interoceptive Awareness (MAIA-2)
6. Responses to Stress Questionnaire - COVID -19 (RSQ-COVID-19) Stress coping style (disengagement, involuntary engagement)
7. Experiences Questionnaire (EQ) (Rumination and Decentering)

Other Exploratory Outcomes and Moderators
1. CAT-MH anxiety (ANX-CAT)
2. CAT-SI risk level
3. Beck Depression Inventory (BDI-2)
4. Referral rates to psychiatric/substance use treatment
5. Credibility and Expectancy
6. Salivary IL-6, TNF-a, IL-1b, IL-8 (from optional sub-study)
7. Oral Fluid Toxicology
8. Ranked Desire of Interventions and Group Times

Additional covariate analyses
1. COVID-19 Fear
2. COVID-19 socio/economic impact
3. COVID-19 status (self-report and salivary IgG)
4. Time since stressor (self-report)

9.6. Data Analyses

9.6.1. Primary Outcome:
For primary outcomes analysis, we will conduct an intent-to-treat, repeated measures design analysis using generalized linear mixed effects models (mixed) to estimate the MBCT-R **(H1.1)** and iCBT **(H1.2)** treatment effects (compared to CHA-MW monitoring on CAT-DI severity at 4, 8, 12, 16, 20 and 24 weeks). Mixed models account for clustering of multiple observations within participants, allowing for person-specific random intercepts and slopes and a variety of covariance structures.[66] For each outcome variable, we will select distribution and link functions that result in the best fit as measured by the Akaike Information Criterion (AIC) and other measures of model fit[67]. The models will include fixed effects terms for treatment group (k=2, compared to the reference group of enhanced CHA-MW monitoring alone), time point (k=6, compared to baseline). The interactions of treatment group and time (k=12) allow us to estimate our treatment effect at each time period. We will compute linear contrasts of predictive margins to test for significant within-group and between-group changes from baseline. Standardized effect sizes will be calculated as the ratio of the linear contrast for the

interaction term of interest divided by the square root of the sum of residual and participant-specific variance. Secondary outcomes **(H2.1-2**) include continuous, binary, and count variables. We match the modeling specification to the outcome. For example, number of tele-visits and number of heavy drinking days will be modeled as count variables and we will use Poisson or negative binomial distributions (depending on degree of dispersion) with log links. Binary outcome models (e.g., toxicology testing at 24 weeks) will use a logistic framework. Two-part models will be considered for continuous and count outcome variables that feature an abundance of zeros. For missing data, we implement multiple imputation methods in Stata (mi procedure).[63,64]

9.6.2. Secondary Clinical Outcomes:
There are several main secondary outcomes, which will be conducted using the same analysis approach as described above for the primary outcome.

The first is change in depression symptom severity comparing iCBT and CHAMindWell alone as measured by CAT-MH Depression Inventory (DI) scores at baseline and during 24 weeks after randomization (week 0,4, 8, 12, 16, 20, and 24). We will use the same linear outcome modeling specification we use for the primary outcome analysis. The second is number of televisits conducted comparing MBCT-R and CHAMindWell during 24 weeks after randomization. The third is number of televisits conducted comparing iCBT and CHAMindWell during 24 weeks after randomization. For these televisit outcomes, we will treat the outcomes as count variables and specify Poisson or negative binomial distributions with log links depending on degree of dispersion (neg. binomial for distribution with overdispersion). The fourth is positive toxicology screens for alcohol or unprescribed substances at 24 weeks comparing MBCT-R with iCBT and CHAMindWell alone. For this binary outcome, we will specify logistic mixed models. The fifth is the number of heavy drinking OR drug use days per month (TLFB) between weeks 0 and 24 comparing MBCT-R with iCBT and CHAMindWell alone. For these count measures, we will consider Poisson and negative binomial specification depending on dispersion and will use zero-inflated versions of these models if there is a high proportion of zeros.

9.6.3. Secondary Stress-Related Affective Reactivity and Salivary Cytokine Outcomes for Optional Sub-Study:

Data on daily experiences will be obtained through REDCap surveys. The Daily Inventory of Stressful Events [24] will be used to assess whether each of 7 types of stressors occurred in the past 24 hours: argument, avoided an argument, stressor at work or school, stressor at home, discrimination, network stressor (i.e., stressful event that happened to a close friend or family member), and any other stressor. In addition, COVID-19 stressors (e.g., family illness, loss of employment) will also be collected. A day will be categorized as a "stressor day" if the participant endorsed at least one stressor, or a "nonstressor day" if the participant indicated that no stressors occurred. *Stressor frequency* will be defined as the percentage of interview days during which at least one stressor occurred (e.g., a person who experienced stressors on 1 of the 5 days had a stressor frequency of 20%).

Stressors will also be summed up and Stress Day Intensity will be stored as scale variable. COVID-19 stressors and daily stressful events will be counted individually and also together so there is ability in exploratory analyses to look at the comparative impacts of COVID-19 stressors versus normal daily stressors.

Affect will be assessed using scales developed for the MIDUS Study [4,68] Participants will report the frequency of emotions using a 5-point scale: 0 = *none of the time*, 1 = *a little of the time*, 2 = *some of the time*, 3 = *most of the time*, 4 = *all of the time*. The Negative Affect (NA) scale consists of 14 items: *restless or fidgety, nervous, worthless, so sad nothing could cheer you up, everything was an effort, hopeless, lonely, afraid, jittery, irritable, ashamed, upset, angry,* and *frustrated*. The Positive Affect (PA) scale consists of 13 items: *in good spirits, cheerful, extremely happy, calm and peaceful, satisfied, full of life, close to others, like you belong, enthusiastic, attentive, proud, active,* and *confident.* Daily NA and PA are calculated by averaging the items within each subscale, and then aggregating scores across interview days. Following prior work [24,69], we will control for *daily affect on non-stressor days* to distinguish between the affect people typically experienced and how they reacted on stressor days. We will not control for mean affect across all days because it overlaps with the concept of affective reactivity (which captures affect on stressor days).

Affective reactivity has been defined as the change in levels of affect on days when stressors occurred, compared to one's typical affect on non-stressor days. Following procedures established in other daily stress studies [70,71] affective reactivity scores will be computed for each participant using a two-level multilevel model in which the occurrence of a daily stressor (yes/no) will be entered as a predictor of PA or NA on day *d* for person *i*:

$$\text{Level 1 (day- level):} \quad \text{Affect}_{di} = \text{a}_{0i} + \text{a}_{1i}(\text{Stressor Day}_{di}) + \text{e}_{di}$$

$$\text{Level 2 (person- level):} \quad \text{a}_{0i} = \beta_{00} + \text{u}_{0i}$$

$$\text{a}_{1i} = \beta_{10} + \text{u}_{1i}$$

At Level 1, a_{0i} is the intercept representing affect on non-stressor days, a_{1i} is the slope representing person *i*'s change in affect on stressor days, and e_{di} is the residual representing day-to-day variability in affect for person *i*. At Level 2, β_{00} and β_{10} represent the sample average levels of affect and affective reactivity, respectively, and u_{0i} and u_{1i} are the variances reflecting person *i*'s deviations from the sample average levels of affect and affective reactivity. These deviations will be outputted from a multilevel model to calculate each person's PA reactivity and NA reactivity slopes.

Daily NA and PA are calculated by averaging the items within each subscale, and then aggregating scores across interview days. Following prior work[69,72], we will control for *daily affect on non-stressor days* to distinguish between the affect people typically experienced and how they reacted on stressor days. *Affective reactivity* has been defined

as the difference in levels of affect on days when stressors occurred, compared to one's typical affect on non-stressor days. In population samples, we have found 10% of days with multiple stressors and 25% of days with a single stressor[73,74]. However, if we assume that COVID-19 has increased the frequency and severity of daily stress, then we can instead examine the relationship of affect with number as well as severity of stressors in a linear fashion. First, we will examine the association between salivary IL-6 and CAT-DI depression severity at baseline to establish the relationship before exposure to study interventions. We will estimate the independent association between salivary IL-6 and depression severity in a linear regression model controlling for baseline characteristics **(H3.1)**. Second, we will build on the H3.1 model to examine the extent to which positive and negative affect reactivity on high stress days are associated with change in salivary IL-6 at baseline. **(H3.2)**. We will also examine the association of affect reactivity with depression severity at baseline holding salivary IL-6 and other participant characteristics constant, and further extend the model with an interaction term to test whether the combination of increased salivary IL-6 and stress-related affective reactivity are associated with increased depression severity above and beyond either item alone. Finally, in an exploratory multilevel structural equation modeling (MSEM) framework, we will investigate the role of salivary IL-6 and stress-related affective reactivity as partial mediators of the relationship between participation in MBCT-R and depression severity. MSEMs allow for the simultaneous estimation of all pathways in the hypothesized partial mediation framework (i.e., direct, indirect, and total) while properly accounting for the repeated measures, intent-to-treat design with the underlying same linear mixed modeling strategy to be used in the primary outcomes analysis **(H3.3)**.[75]

9.6.4. Exploratory Outcomes:

Other secondary clinical outcomes include changes in anxiety (ANX-CAT), neurovegetative symptoms (BDI-2), perceived stress scale (PSS-14), number of televisits and referral rates to psychiatric/substance use treatment as recorded from the Electronic Health Record (EHR) from randomization to 24 weeks. We will include several exploratory mechanisms: COVID-19 status, COVID-19 impact (RSQ-COVID-19), COVID-19 fear, experiential avoidance (BEAQ), stress coping style (disengagement, involuntary dis/engagement) (RSQ-COVID-19), rumination (EQ), decentering (EQ), self-compassion (SCS-SF), emotion regulation (DERS), and interoceptive awareness (MAIA-2).We expect randomization to balance the treatment and control groups on participant characteristics. However, in the chance case of imbalance of assignment, we will add the following variables as measurable covariates in regression models: other CAT-MH scores besides CAT-DI, age, gender, education level, past meditation experience, race/ethnicity, employment, acute change in employment past 6 months. We will also add any other baseline characteristics that may be significantly imbalanced after randomization. We will record level of home skills use weekly to measure mindfulness practice dose in MBCT-R and coping skills use in iCBT. We adapt the mixed model specification depending on variable outcome types as described in 9.6.2. For the substudy, we will examine associations between salivary IL-6 concentrations, negative affect reactivity, and depression severity among the full sample, including healthy controls and people with depression, by conducting a linear regression using IL-6 levels as the dependent variable

and negative affect reactivity and depression severity *T*-scores as the independent variables. We will also conduct an independent samples t-test to examine group-level differences in IL-6 concentrations between healthy controls and people with depression.

For secondary and exploratory outcomes, we will use the Benjamini-Hochberg false discovery rate (FDR) procedure[76], which accounts for multiple comparisons. We will implement FDR according to Cao et al.[77] in which a cutoff p value is determined for a family of similar variables and analyses (family-wise error rate = 0.05).[78–80] Statistical significance will be determined for the following analysis families: between-group main secondary clinical outcomes (family size, n = 5), and stress-related affectivity outcomes (n= 4), inflammatory marker outcomes (n=4), and other exploratory outcomes (n will be defined as exploratory analysis proceeds).

10. DATA COLLECTION AND QUALITY ASSURANCE

10.1. Data Collection Forms

<u>Sources of Research Materials for Participants:</u>

The following research material will be obtained:

1. Responses to assessments stored in REDCap
2. Responses to CAT-MH interviews stored into a CHA secure Google Drive delivered weekly by Adaptive Testing Technologies (ATT).
3. Saliva samples
4. Electronic health records (to reference referrals and to check for exclusion)
5. Audio-video tapes of MBCT-R sessions for fidelity ratings

The following is the full list of measures for this study.

A brief **demographics survey** will note participant race, ethnicity, primary language, income, and other quantifiable attributes. Duration: 4 min.

We will conduct a Substance Use History at screening and Week 24. We will use the **PhenX toolkit to assess Lifetime use of Substances, Substance Use 30-Day Frequency, alcohol 30-day quantity and frequency** during screening. We will also conduct 30-day alcohol and substance use using the **TimeLine Follow Back (TLFB) Method** at Week 24 visit by telephone or video conference[81]. (PhenX duration: 8 minutes, TLFB Method: 15 minutes)

Brief Experiential Avoidance Questionnaire (BEAQ)[82]: The 62-item Multidimensional Experiential Avoidance Questionnaire (MEAQ) was recently developed to assess a broad range of experiential avoidance (EA) content. However, practical clinical and research considerations made a briefer measure of EA desirable. Using items from the original 62-item MEAQ, a 15-item scale (BEAQ) was created that tapped content from each of the MEAQ's six dimensions. Items were selected on the basis of their performance in 3 samples: undergraduates (n = 363), psychiatric outpatients (n = 265), and community adults (n = 215). These items were then evaluated using 2

additional samples (314 undergraduates and 201 psychiatric outpatients) and cross-validated in 2 new, independent samples (283 undergraduates and 295 community adults). The resulting measure (Brief Experiential Avoidance Questionnaire; BEAQ) demonstrated good internal consistency. It also exhibited strong convergence with respect to each of the MEAQ's 6 dimensions. The BEAQ demonstrated expected associations with measures of avoidance, psychopathology, and quality of life and was distinguishable from negative affectivity and neuroticism. Duration: 4 min

The **Difficulties in Emotion Regulation (DERS) Scale** [83] is a 36-item self-report scale designed to assess emotional dysregulation. The scale assess 6 aspects of emotional dysregulation: non-acceptance of emotional responses ("When I'm upset, I become embarrassed for feeling that way"), difficulties engaging in goal directed behavior ("When I'm upset, I have difficulty thinking about anything else"), impulse control difficulties ("When I'm upset, I lose control over my behaviors"), lack of emotional awareness ("When I'm upset, I take time to figure out what I'm really feeling (reverse-scored)", limited access to emotion regulation strategies ("When I'm upset, it takes me a long time to feel better"), and lack of emotional clarity ("I have no idea how I am feeling"). Duration: 8 min.

The **Perceived Stress Scale (PSS) Scale**[84] uses 14 items to measure the degree to which situations in life are stressful. Items are designed to evaluate how overloaded, unpredictable, and uncontrollable one finds one's life. Each item is scored on a 5-point Likert scale from 0 (*Never*) to 4 (*Very often*). An example question is, "In the last month, how often have you felt difficulties were piling up so high that you could not overcome them?" Positively stated items are reverse scored before all scale items are summed to yield a total score. Duration: 4 min.

The short-form **Self-Compassion Scale-Short Form (SCS-SF)**[85] is an abbreviated 12-item form of the original 26-item Self-Compassion Scale. This scale evaluates six different aspects of self-compassion: Self-Kindness (e.g., ''I try to be understanding and patient toward those aspects of my personality I don't like''), Self-Judgment (e.g., ''I'm disapproving and judgmental about my own flaws and inadequacies''), Common Humanity (e.g., ''I try to see my failings as part of the human condition''), Isolation (e.g., ''When I feel inadequate in some way, I try to remind myself that feelings of inadequacy are shared by most people"), Mindfulness (e.g., ''When something painful happens I try to take a balanced view of the situation''), and Over-Identification (e.g., ''When I'm feeling down I tend to obsess and fixate on everything that's wrong.''). The scale is scored on a 5-point Likert scale (1 = Almost never; 5 = Almost always), and negative subscale items are reverse scored. Duration: 2 min.

The **Multidimensional Assessment of Interoceptive Awareness (MAIA-2)**[86]is a 37 item self-report scale designed to assess multiple aspects of interoception and interoceptive awareness. The scale assesses 8 aspects of interoceptive awareness: noticing ("I notice when I am uncomfortable in my body"), not-distracting ("I do not notice (I ignore) physical tension or discomfort until they become more severe"), not-

worrying ("I start to worry that something is wrong if I feel any discomfort"), attention regulation ("When I am in conversation with someone, I can pay attention to my posture"), emotional awareness ("I notice that my breathing becomes free and easy when I feel comfortable"), self-regulation ("When I am caught up in thoughts, I can calm my mind by focusing on my body/breathing"), body listening ("I listen to my body to inform me about what to do"), and trusting ("I feel my body is a safe place"). Duration: 5 min.

Experiences Questionnaire (EQ)
This is a 20-item self-report measure of decentering[87] and is conceptualized as a protective factor and capable of measuring resilience to depressive relapse. The EQ uses a 5-point Likert scale with responses from "never" to "all the time". A sample item from the decentering subscale is "I can observe unpleasant feelings without being drawn into them." Psychometric properties are: (reliability: Cronbach's $\alpha = .89$; convergent validity: $r > .46$; and divergent validity: $r < -.35$).[88] Duration: 5 min.

The **Beck Depression Inventory (BDI-II)** is a 21-item self-report scale designed to assess depression severity over the past 2 weeks with well-established internal consistency, reliability and validity.[89,90] Duration: 7 min.

Optional Daily Diaries

These diaries will have 2 sections, including inventory of stressful events and affect reactivity scales.

1. The **Daily Inventory of Stressful Events**[91] is used to assess whether each of 7 types of stressors occurred in the past 24 hours: argument, avoided an argument, stressor at work or school, stressor at home, discrimination, network stressor (i.e., stressful event that happened to a close friend or family member), and any other stressor. A day is categorized as a "stressor day" if the participant endorsed at least one stressor, or a "nonstressor day" if the participant indicated that no stressors occurred. Stressor frequency is defined as the percentage of interview days during which at least one stressor occurred (e.g., a person who experienced stressors on 1 of the 5 days had a stressor frequency of 20%). Additional questions about COVID-19 stressors will be added to inventory of daily stressors. We will collect daily diaries for at least 5 days within a 7-day window at baseline, between study weeks 9-12, up to 12 months post-intervention, or during a single time point collection for healthy controls with saliva collection ideally (*but not necessarily*) on a day when there is completed diary surveys the day before, day of collection and day after. Duration: 10 min.

2. Positive and Negative Affect Reactivity Scales were developed for the MIDUS Study[4,92] Participants report the frequency of emotions using a 5-point scale: 0 = none of the time, 1 = a little of the time, 2 = some of the time, 3 = most of the time, 4 = all of the time. The NA scale consists of 14 items: restless or fidgety, nervous, worthless, so sad

nothing could cheer you up, everything is an effort, hopeless, lonely, afraid, jittery, irritable, ashamed, upset, angry, and frustrated. The PA scale consists of 13 items: in good spirits, cheerful, extremely happy, calm and peaceful, satisfied, full of life, close to others, like you belong, enthusiastic, attentive, proud, active, and confident. Daily NA and PA are calculated by averaging the items within each subscale, and then aggregating scores across interview days. During the 8 study days, Cronbach's alpha ranges from 0.83 to 0.87 for daily NA and from 0.92 to 0.95 for daily PA. As per previous studies with this measure[24], we will control for daily affect on nonstressor days to distinguish between the affect people typically experienced and how they reacted on stressor days. We will not control for mean affect across all days because it overlaps with the concept of affective reactivity (which captures affect on stressor days). Duration: 10 min.

Salivary Samples

The minimally invasive nature of sample collection is a key advantage of oral fluid over traditional biospecimens. An IDE is not required for these saliva tests because they are non-invasive and are not being evaluated to determine safety or effectiveness. The two dominant methods are collection of whole saliva by sponge method (an absorbent material placed in the mouth) and passive drool. A sponge method of collection will be used for required oral fluid toxicology testing. Oral fluid toxicology testing will be supervised via videochat with a study staff member to ensure that there is no tampering with the collection. We intend to use passive drool for optional IL-6 cytokine stress collection since we have multiple cytokines being collected. A passive drool method of collection will be used for optional COVID-19 IgG antibody testing.

Preparation: All subjects will be sent a package after enrollment and initial screening with an Oral Fluid Toxicology kit for saliva sample collection via video conference with a research coordinator or a research assistant.

1. **Optional COVID-19 IgG samples**: Antibodies for COVID-19 will be sampled during baseline at the CMC by a CMC research coordinator, research assistant, or other trained CMC staff member and again between weeks 9-12 among participants enrolled in the salivary sub-study to establish whether they were exposed to COVID-19 during the study. We may also contact participants for one saliva sample collection up to 12 months following the intervention period. We may also contact CHAMindWell participants to enroll as healthy controls for a single time point collection. Samples will be immediately stored in a locked CMC freezer by the research coordinator. Testing will be conducted at Salimetrics using established methods for immunoglobulin analysis[63]. Duration: 15 min.

2. **Optional cytokine samples**: These will be collected during weeks -4-0 (pre-intervention) and weeks 9-12 (post-intervention) during diary collection periods. Participants will come to the CMC for saliva sample collection by a research

coordinator once at baseline and once after the study intervention between weeks 9-12 with saliva collection ideally (*but not necessarily*) on a day when there is completed daily diary surveys the day before, day of collection and day after. We may also contact participants for one saliva sample collection up to 12 months following the intervention period. Research coordinators will place samples immediately in the freezer prior to shipment to Salimetrics for analysis. Duration: 4 min.

3. **Collection process of both Cytokine and COVID-19 IgG salivary samples:** Care will be taken to freeze immediately and to prevent freeze/thaw cycles because of the risk to cytokine levels with extended periods of thaw (see Figure X,Y)[63]. Participants will come to the CMC for saliva sample collection by a research coordinator at baseline, study weeks 9-12, or up to 12 months following the intervention. All samples will be safely stored in a locked freezer at CMC prior to shipment to Salimetrics. Only members of the study team will have access to the sample freezer.

Figure X: *Room temperature storage decreased median salivary cytokine concentrations (Riis, Granger, under review)*

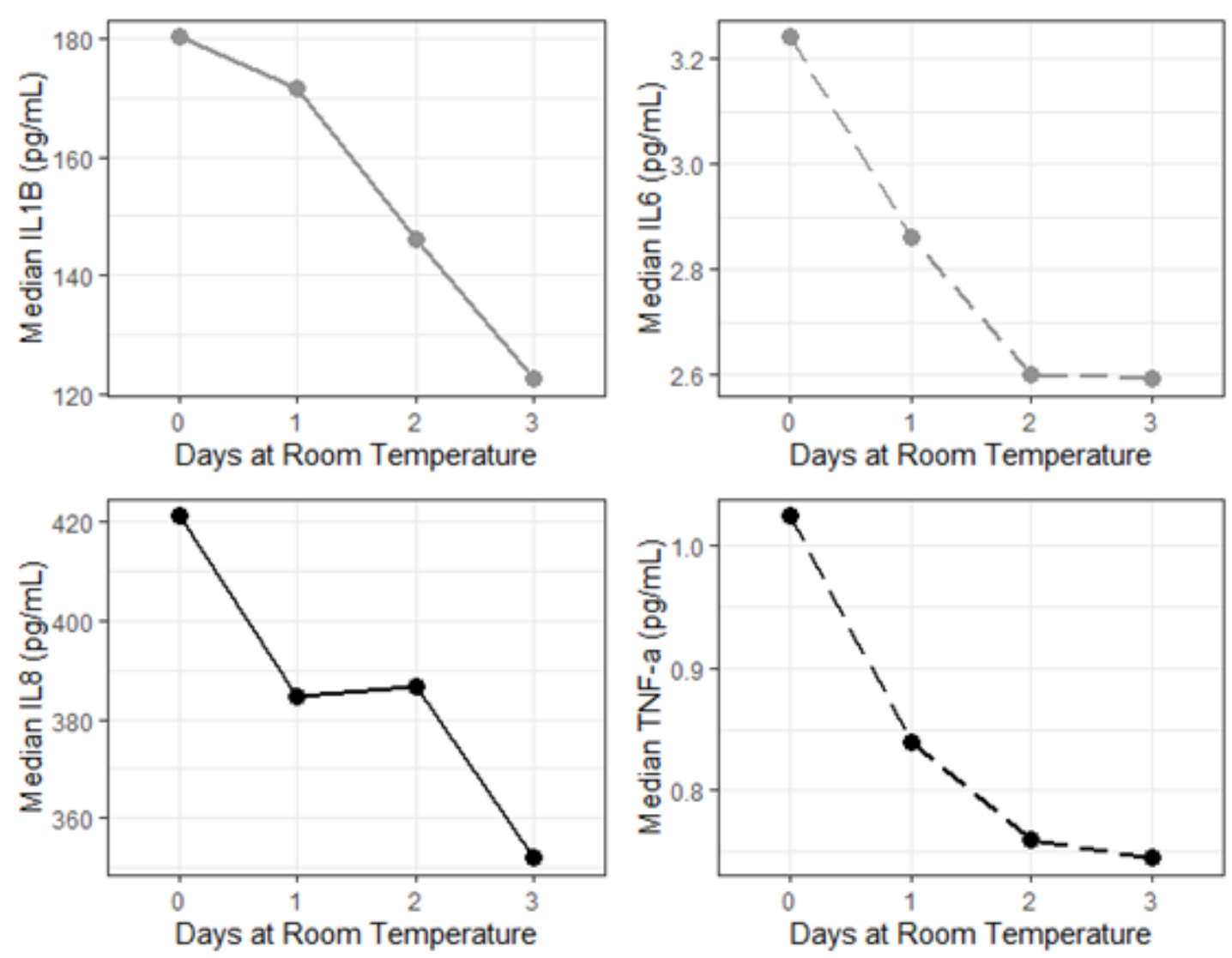

Figure X: *Exposure to freeze/thaw cycles decreased median salivary cytokine concentrations (Riis, Granger, under review)*

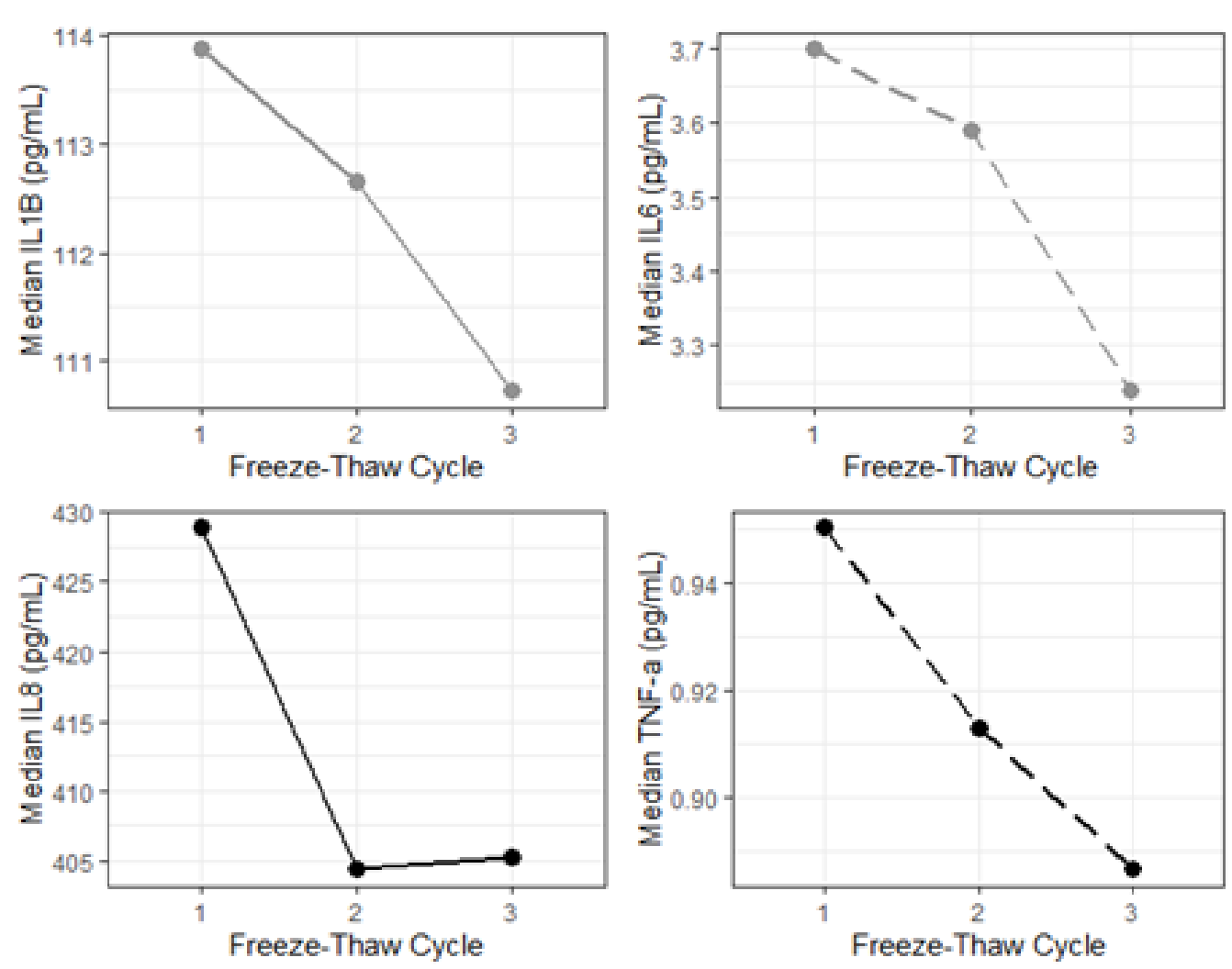

4. **Processing of Cytokine and COVID-19 IgG salivary samples**: Periodically (but not more than 6 months from the earliest collected sample to be sent in the package), a

bioshipper container will be filled with dry ice and shipped via FedEx (or similar approved carrier) to Salimetrics where the samples with be analyzed. Samples may be held until study completion before sending depending on funding and capacity for additional analyses. We will prioritize the analysis of 70 MBCT-R and CHA-MindWell alone subject's samples, but will include iCBT samples as well if funding allows. Additional unused samples may be saved and stored at Salimetrics storage facility or in the CMC freezer for future use including genomics or additional biomarkers not currently identified.

5. **Analysis of IL-6 salivary cytokines samples** as part of optional sub-study will be conducted using a 4-plex proinflammatory cytokine panel for IL-1β, IL-6, IL-8, and TNF-α (Meso Scale Discovery®; cat# K15025C) at Salimetrics laboratory in Irvine, California[63].

6. **Daytime Oral Fluid Toxicology Testing**[93]:

Daytime oral fluid toxicology tests will be sent at screening to participants' homes to be taken between weeks -4 and 0 and sent again between weeks 6-8 to be taken at week 24.

Participants will be asked to complete daytime oral fluid toxicology testing during screening (weeks -4 – 0) and post-intervention (week 24). This process takes about 15 minutes in total. During this time, participants are required to video chat with a research coordinator to ensure that the results are properly recorded and that there is no tampering with the samples. First participants will unscrew the collector cap and pull out the collection stick with the sponge from the collection chamber. Next, participants will put the collector stick between their tongue and cheek to collect oral fluid by swabbing the inside of their mouth and gently chewing the sponge until the saturation stick turns red, this takes a total of 3 minutes. Once this is complete, participants remove the sponge from their mouth, and place the collection stick into the collection chamber. Finally, participants secure the cap and shake three times. At this time, the research coordinator will start the timer for 10 minutes, after which the research coordinator will ask the participant to peel off the label. Results will be recorded by a saved screenshot image in secure google drive with the study ID and acrostic and date of test as the only identifiers. The research coordinator will record the results in REDCap, which will be double checked by another RC. Duration: 15 min.

<u>Use of Salivary Samples for Future Research</u>

Patient identifiers will be removed from the identifiable private information or identifiable biospecimens (saliva samples). After removal, biospecimens could be used for future research studies or distributed to another investigator for future research studies without additional informed consent. The research might include whole genome sequencing. We will save these saliva samples in a locked refrigerator in a double locked

area at CMC in case funding for this type of genomic testing becomes available in the future with follow-up grants.

CAT-MH Interviews
Participants are sent a link to complete the CAT-MH (Computer Adaptive Testing for Mental Health) interview on a computer, tablet or phone. The interview is delivered via a secure HIPAA-compliant server: either through a CHA IT-approved process using the ATT server or CHA's REDCap server. ATT is the company who developed the CAT-MH software for behavioral health measurement. Items from each of the modules for depression (CAT-DI), anxiety (ANX-CAT), mania and hypomania (M/HM-CAT), PTSD (PTSD-CAT), psychosis (CAT-Psychosis), suicidality (SS-CAT), and substance abuse (SUD-CAT) [57,58] are chosen from large item banks based on multidimensional item response theory, adapting each item presented to the individual's severity so that different individuals are tested with different items depending on their severity level. [52] This allows for rapid testing – 2-12 minutes, depending on the number of domains tested – compared to 1-1.5 hours for a structured clinical interview, and greater precision. It is easy for patients to fill in online[50]. Duration: 2-12 min.

Credibility/Expectancy, Rank Desire for each intervention option.
Treatment expectancy and credibility for each arm will be assessed with the 6-item Credibility/ Expectancy Questionnaire (CEQ)[94] modified slightly to substitute the word 'Class' for 'Therapy' in the instructions. Duration: 1 min.

Responses to Stress Questionnaire COVID-19 (RSQ-COVID-19)[95]
The adult self-report COVID-19 Responses to Stress Questionnaire is a 72-item survey. Responses are recorded on a 4-point Likert scale (1 = Not at All, 4 = Very/A lot). Items in the RSQ cover 5 factors of coping and stress responses: primary control engagement coping (i.e., problem solving, emotional expression, emotional modulation), secondary control engagement coping (i.e., positive thinking, cognitive restructuring, acceptance, distraction), disengagement coping (i.e., avoidance, denial, wishful thinking); involuntary engagement (e.g., physiological arousal, rumination), and involuntary disengagement (e.g., emotional numbing). Duration: 20 min.

UCI Oral Health Questionnaire[96]

The UCI Oral Health Questionnaire is a 12-item survey that asks sociodemographic questions, questions about oral and physical health, as well as additional oral health questions drawn from the WHO Oral Health Survey.[97] The questionnaire includes basic questions about dental health characteristics of participants to be used as an exclusion criteria for the salivary collection portion of the study. Duration: 4 min

COVID-19 Fear[98]

The COVID-19 Fear scale is a 7-item questionnaire. Responses are recorded on a 5-point Likert scale (1 = Strongly Disagree to 5 = Strongly Agree). Responses to scale items

were found to positively correlate with perceived vulnerability, hospital anxiety, and depression. Sample questions include "I am most afraid of coronavirus-19," and "It makes me uncomfortable to think about coronavirus-19." Duration: 3 min.

COVID Status Self-Report

Participants will record their current COVID-19 status. This self-report measure will be recorded on REDCap. Duration: 1 min.

Home Skills Use Diary

The **Home Skills Use Diary** will be completed for the 8 weeks during the intervention period and will come in two types (1: weekly mindfulness practice and resource use diary for the MBCT-R group and 2: General CBT coping skills use diary card for both the iCBT and CHAMindWell arms). Duration: 5 min.

1. Carmody et al.[99] emphasizes that improvements in mindfulness, symptoms, and wellbeing are significantly related to formal and informal mindfulness practice. This 12-item weekly diary asks participants to specify the type and duration of formal mindfulness techniques they completed each day for the past week, the type and frequency of informal techniques each day, and their mindfulness resource use will be assessed by daily frequency per week of community or mobile mindfulness resources as listed on the Community Resources. Four formal mindfulness techniques include body scan, sitting meditation, loving kindness, and mindful movement. Four informal mindfulness practices include techniques like breathing space, mindful awareness of eating, informal kindness, and mindful walking. Four resources include use of Mindfulness/Wellness centers, online recordings, mobile apps and MBCT-R recordings provided online. A participant's total formal practice duration as well as informal practice and resource utilization counts for the week will be calculated by study personnel. Participants will fill out the practice diary on an online REDCap form. This diary is previously used in MBI studies[80] and will be adapted specifically for MBCT-related skills.

2. For iCBT and CHAMindWell alone arms we will use a Homework Practice Questionnaire[100] self-report measure asks participants to record the frequency of engaging in CBT practices including behavioral activation, cognitive reappraisal, core beliefs, exposure and thought records. It demonstrates acceptable test-retest reliability and validity based on correlations between client and therapist ratings. Previous studies have shown that frequency of skill use leads to depression symptom reduction in CBT.[101]

The **Adverse Event Patient Participant Self-Report Form** will be systematically assessed at assessment timepoints (baseline, 4-weeks, 8-weeks, 12-weeks, 16-weeks, 20-weeks and 24-weeks). In addition, group leaders and research coordinators will be trained to identify and report any adverse events that occur or are reported during weekly group visits. All AEs will be reviewed monthly by the PI, and SAE's will be reviewed within 24 hours. Please see adverse events section (7.3) above. Duration: 5 min

10.2. Data Management

The below outlines data storage in this study, which data will be stored, and what PHI will be stored if applicable. Only CHA IRB approved study staff will have access to the below, which summarizes the entirety of data collected in this study. Data will flow between CHA CMC and CHA Health Equity Lab, and PSU Almeida lab for analysis. Data from Salimetrics will be sent via a CSV or XLSX file to CHA. CHA will send that data to PSU. All data collection will be centralized to the following 5 locations below.

We will receive MoodGYM administrative login credentials to access module data. This data will be saved to our secure G-Suite Database as CSV files.

CHA Secure G-Suite Databases: Secure CHA G-suite database access will be granted through secure logins to CHA IRB approved study staff only. All data kept in G-suite databases will be identified by study number only, with the exception of the one linking sheet that links name and study number, and the G-suite folder where the pdfs of consent forms signature are kept. Consent forms signature pages will be captured as a pdf and saved to the G-suite consent form database. Electronic Medical Record exports may be completed by the data analyst in Health Equity Lab and uploaded to a separate CHA Secure G-suite database if they have not already been entered directly into REDCap by the coordinator. CAT-MH data will be transferred by ATT to a confidential G-suite Drive for CHAMindWell that they have access to deliver file drops every Monday morning and will be accessible through REDCap. Study status tracking for each participant will be maintained on a G-suite database. Audio-video recordings of MBCT-R group sessions will be recorded by Zoom, saved to the HIPAA-compliant Zoom cloud or to a coordinator's computer but only accessible within the CHA Zoom research account on the coordinator's computer, then uploaded to a G-suite database and erased from the coordinator's computer Zoom account. Images of MoodGym completion report among iCBT arm will be emailed to RCs by participants or screen shot by video and then stored in G-suite. Any notes taken by study staff during any portion of the study will be recorded in a google document within our secure G Drive. All study staff will be alerted to the fact that no paper notes will be recorded throughout the trial due to privacy issues related to working from home during COVID-19.

PHI kept on Secure G-suite databases:
Screening database: Name, birthday, medical diagnosis, MRN, phone number, email address
Linking database: Name, study number, study acrostic
Consent form folder, stored in secure G-Suite drive separate from all other study documents: PDFs of consent form signature pages uploaded

Secure REDCap Databases: Electronically signed informed consent forms and informed consent assessments will be stored in REDCap and completion will be visible from REDCap dashboard. All participant survey responses will be kept in a secure REDCap database. Only CHA IRB-approved study staff will have access to these

databases. REDCap (Research Electronic Data Capture) [[i]] is a secure, HIPAA compliant, web-based application designed to support data capture for research studies. This platform provides the following elements: 1) an intuitive interface for validated data entry; 2) audit trails for tracking data manipulation and export procedures; 3) automated export procedures for seamless data downloads to common statistical packages; and 4) procedures for importing data from external sources. The REDCap software was developed by Vanderbilt University and has been obtained and installed for usage at the Cambridge Health Alliance.

PHI kept on REDCap database: demographic survey response data (home Zip+4 code, e-mail address, and age).

Double-locked lockboxes and filing cabinets: Given COVID-19, all data will be stored using online secure databases. If necessary, lockboxes will be transported to and from each site by the site research coordinator within a secure vehicle or in a locked bag located on their person during transit.

Locked Biospecimen Freezer:
All biospecimen samples will be coded with study number and acrostic and will not include accessible PHI. These will be stored in a locked CMC freezer in a locked research space. This freezer has been approved by Tom Leslie who is CHA's property manager for CMC space.

MoodGYM:
Participants enrolled in iCBT will be given a unique user token ID which they will use to register on the MoodGYM website (https://moodgym.com.au). Depression and anxiety quiz scores, progress through the site (with date-stamps for when modules are started/completed), number of logins, and time in each module will be associated with the individual user token in the MoodGYM site. ehub Health will not have access to the key to link the user tokens to the participant. CHA will receive administrative login credentials to access reports of these data, which only we will be able link to the specific participant.

Collaboration
Penn State will receive completely de-identified data from CHA. As such, we will seek a non-Human Subjects Research determination from Penn State's IRB office. The data they will receive includes: medical diagnoses, current prescribed medications, salivary cytokine pre- and post-salivary samples, COVID-19 IgG salivary test results, Oral Fluid Toxicology test results, demographic information, survey responses, and CAT-MH results. They will receive the following demographic information: gender, sexual identity, racial background, ethnicity, English as first language, primary language, years of education, training or technical education, profession/trade/skill, years of education of mother, years of education of father, employment status, income in the last year, length of longest full-time job, number of days paid for work in past 30 days, marital status, living

arrangements, if they live with anyone with current alcohol problems or use of non-prescribed drugs, if they have been in a controlled environment in the past 30 days, information about interpersonal relationships, if they receive pension for physical disability, and hopes for improvements they will see as a result of the program. Penn State will also receive de-identified survey response data for the following surveys: oral health saliva screening, alcohol/substance use history, SMME, Daily Inventory of Stressors, Positive and Negative Affect Reactivity, Experiences Questionnaire, BEAQ, DERS, PSS-14, SCC-SF, MAIA-2, BDI-II, RSQ-COVID-19, COVID-19 Fear, COVID status self-report, and Home Skills Use Diary.

Data Linkage to participants and access to data:
The secure web application REDCap will be used for data collection. Data will be entered directly into REDCap by participants. Any data entry not conducted by participants will be conducted only by members of the research team. All data collection will take place under the supervision of the Principal Investigator (Dr. Schuman-Olivier, MD) or a Research Coordinator. Substitute codes will be used to label all sources of participant information and access to identifiable data will be limited to researchers directly involved in the study. The Data Analyst will be responsible for coding data and exporting coded data from REDCap to STATA (14 or newer version), R (3.5.3 or newer version), or SAS (9.4 or newer version). All identifiable data will be destroyed 7 years after study completion and will not be maintained for future uses not specified in this research plan.

Data Sharing

The data generated in this grant will be presented at national or international conferences and published in a timely fashion. All final peer-reviewed manuscripts that arise from this proposal will be submitted to the digital archive PubMed Central. Primary outcome data will be deposited to appropriate public repositories (e.g., Harvard Dataverse, Open Science Framework) prior to the time the main study findings are accepted for publication. This will be listed in the consent form.

We will ensure that the clinical trial is registered to ClinicalTrials.gov no later than 21 days after enrollment of the first participant, and that a summary of study results will be available on ClinicalTrials.gov no later than 12 months after the primary completion date. Informed consent documents for the study will include a specific statement relating to posting of study information and results at ClinicalTrials.gov. CHA has an internal policy in place to ensure that clinical trials registration and results reporting occur in compliance with policy requirements.

Description of Plan for Data Quality and Management:
The PI, Co-PI, and senior RC, and data analyst will review data collection, data completeness and accuracy as well as protocol compliance on a monthly basis.

Survey session data will be entered by participants into REDCap only. The data analyst will review all REDCap data collection forms on an ongoing basis for data completeness and accuracy as well as protocol compliance and provide a monthly report to the PI about missing data.

Adherence with expected study assessment visits will be reviewed twice monthly by the senior RC with the two RCs, monthly with the PI, and twice yearly by the study methodologist and DSMB.

Frequency of Review—The frequency of data review for this study differs according to the type of data and can be summarized in the following Data Quality Monitoring Table 4.

Table 4. Data Quality Monitoring Table

**Data type**	**Frequency of review**	**Reviewer**
Study progress, recruitment, ICF and I/E review, and safety	Weekly	Senior Research Coordinator (SRC)
Data collection, data quality/completeness/accuracy protocol compliance	Weekly	Senior Research Coordinator (SRC)
Study progress, recruitment, participant accrual (including compliance with protocol enrollment criteria)	Monthly	PI, SRC, Co-PI, Data Analyst
Data collection, data quality/completeness/accuracy protocol compliance	Monthly	Data Analyst, SRC, PI, Co-PI
Participant accrual (including compliance with protocol enrollment criteria)	Twice-yearly	PI, Co-PI, Study Methodologist, DSMB
Status of all enrolled participants, as of date reporting	Twice-yearly	PI, Co-PI, Study Methodologist, DSMB

Adherence data regarding study visits and intervention	Twice-yearly	PI, Co-PI, Study Methodologist, DSMB
AEs and study safety	Per occurrence & Monthly	PI, Co-PI
Minor Protocol Deviations	Per occurrence & Monthly	PI, Co-PI
Major Protocol Violations	Per occurrence	PI, Co-PI, IRB, DSMB, NCCIH
SAEs	Per occurrence	PI, Co-PI, IRB, DSMB
SAEs (unanticipated, serious, and possibly related to study)	Per occurrence	PI, Co-PI, IRB, DSMB, NCCIH

10.3. Quality Assurance

10.3.1. Training

Co-PI Dr. Fulwiler will be one of the MBCT-R intervention group leaders early on in the study while other group leaders are being trained (see group leader training below). Dr. Fulwiler will have limited access to de-identified data for the purpose of data analysis. Other group leaders will be trained MBCT program staff, trained by CMC to administer MBCT in other contexts and then trained for MBCT-R adaptations. Group leaders will not have access to any study documents or information. They will act in a program leader capacity as population mental wellness programs staff, not in a study capacity. All groups will be led by a leader who will be a credentialed clinician with an active clinical license at CHA for providing healthcare.

MBCT-R training for group leaders: Prerequisites to be eligible for training as a group leader for MBCT-R are: 1) mental health training and experience, i.e. an advanced degree in mental health (social work, psychology, psychiatry) including training in Cognitive-Behavioral Therapy, and at least two years of clinical experience in the field including experience with running groups; 2) personal experience with a regular mindfulness meditation practice; and 3) Training and experience with MBCT including attendance at the 5-day intensive foundational training and experience teaching at least one MBCT class with supervision and mentoring by a qualified MBCT mentor. Candidates must also be thoroughly familiar with the MBCT curriculum (Mindfulness-Based Cognitive

Therapy for Depression, Second Edition, Segal, Z.V., Williams, J.M.G., Teasdale, J.D., (2012) Guildford Press, New York).

MBCT-R training will start with a day-long program in a simulated classroom setting involving role plays, practice teaching, and didactics covering adaptation of the MBCT curriculum for COVID-related resilience skills. Participants will then be eligible to co-teach MBCT-R with Dr. Fulwiler, a nationally recognized MBCT mentor, who will provide mentoring and feedback. Leader trainees will be required to co-lead at least two MBCT-R classes with Dr. Fulwiler with mentoring before being eligible to co-lead a MBCT-R class on their own. Trainees will be rated for both MBCT and MBCT-R teaching competence using the Mindfulness-Based Intervention Teaching Assessment Criteria (MBI:TAC).[59] Only teachers who are rated as Competent or above will be allowed to teach MBCT-R classes for the study.

MBCT-R Group Leader Fidelity and Adherence

Fidelity and Adherence: MBCT-R group leaders will be mentored by Co-PI Dr. Fulwiler when teaching MBCT-R classes. Those who are eligible for solo teaching (see group leader training description in Section 5.2 above), will attend weekly group supervision/mentoring sessions with Dr. Fulwiler throughout the 8-week class. All groups will be audio-video recorded. In order to prevent drift from the manual, 10% of session audio records will be reviewed by trained experts, and all sessions will be assessed for adherence to the manual using fidelity checklists that are completed by site research coordinators participating in the group.

10.3.2. Quality Control Committee

The Senior Research Coordinator will review enrollment reports, adverse events, data quality, missing data, database quality, event reports from group leaders, and other aspects of quality control address study each week.

MBCT-R Faculty mentor (Gawande) will review 10% of audio-video recordings of the intervention, conduct MBI:TAC and review adherence checklists to ensure fidelity to the intervention.

Drs. Schuman-Olivier and Fulwiler will meet monthly with the senior research coordinator and data analyst to review enrollment, adverse events, data quality, missing data, database quality, event reports from group leaders, and other aspects of quality control.

Dr. Fulwiler will meet weekly with MBCT-R group leaders for weekly mentorship during MBCT-R groups.

10.3.3. Metrics

All self-report survey items for both primary and secondary measures are coded as required fields in REDCap to prevent missed items. During the RCT, group leader

fidelity checklists are reviewed each week by Co-PI Dr. Fulwiler or MBCT-R faculty mentor Dr. Gawande. All data are checked each month for missingness within each measure and for missing surveys, by the data analyst.

10.3.4. Protocol Deviations

Protocol deviations are captured weekly in the research coordinator meeting, which is overseen by the Senior Research Coordinator, and in the study implementation weekly meeting, which is overseen by the PI. Deviations are documented in the Protocol Deviation log, which is reviewed monthly by the PI and is reviewed twice yearly by the DSMB, and annually by the CHA IRB.

10.3.5. Monitoring

Data Safety Monitoring Board:
We will have a Data Safety Monitoring Board that meets twice yearly, consisting of 3 members, with at least one psychiatry clinician researcher (Joji Suzuki, MD), at least one member who has expertise in mindfulness-based interventions (Sarah Bowen, PhD), and at least one epidemiologist/statistician (Linda Valeri, PhD).

Safety Review Plan:
Study progress and safety will be reviewed monthly (and more frequently if needed). Progress reports, including participant recruitment, retention/attrition, and AEs, will be provided to the DSMB every 6 months for the DSMB meeting. An Annual Report will be compiled and will include a list and summary of AEs. In addition, the Annual Report will address (1) whether AE rates are consistent with pre-study assumptions; (2) reason for dropouts from the study; (3) whether all participants met entry criteria; (4) whether continuation of the study is justified on the basis that additional data are needed to accomplish the stated aims of the study; and (5) conditions whereby the study might be terminated prematurely. The Annual Report will be sent to the DSMB and will be forwarded to the IRB. The IRB and other applicable recipients will review progress of this study on an annual basis. The PI will also send copies of signed recommendations and comments from the DSMB to the NCCIH Program Officer within 30 days of each monitoring review.

Study Report Outline for the DSMB (Interim or Annual Reports):

The study team will generate Study Reports for the DSMB and will provide information on the following study parameters: rate of participant accrual and compliance with inclusion/exclusion criteria, status of all enrolled participants, adherence data regarding study visits and intervention, AEs, and protocol violations. Study report tables will be generated only from aggregate (not by group assignment) baseline and aggregate safety data for the study population. A separate Closed Safety Report, with masked group baseline and safety data, will be generated for the DSMB by a designated unmasked member of the team but will not be reviewed by the study PI.

This study will be stopped prior to its completion if: (1) the intervention is associated with adverse effects that call into question the safety of the intervention; (2) any new information becomes available during the trial that necessitates stopping the trial; or (3) other situations occur that might warrant stopping the trial.

The PI will include an assessment of futility (if relevant) in the annual progress report to NIH (using statistical means such as predictive probability, if appropriate) and will consult with the study monitors to assess the impact of significant data loss due to problems in recruitment, retention, or data collection. The study may also be discontinued at any time by the IRB, the NCCIH, or other government agencies as part of their duties to ensure that research participants are protected.

11. PARTICIPANT RIGHTS AND CONFIDENTIALITY

11.1. Institutional Review Board (IRB) Review

This protocol and the informed consent document and any subsequent modifications will be reviewed and approved by the IRB or ethics committee responsible for oversight of the study.

11.2. Informed Consent Forms

Informed consent will be obtained during the Informed Consent Session as described in Section 6 above. The consent session will begin with a verbal review of key information about the study over videoconference. Consent will be obtained through REDCap with an electronic signature given COVID-19 risk for in-person contact precludes in person informed consent and the purpose of this study is to conduct a remotely delivered program. The consent form will describe the purpose of the study, the procedures to be followed, and the risks and benefits of participation. All informed consent documents are approved and reviewed on an annual basis by the CHA IRB. A signed consent form will be obtained from each participant. Given that the intervention requires a 7th grade reading level and only adult participants can enroll, we will not obtain informed consent for this study from a person who requires a guardian.

11.3. Participant Confidentiality

All participants will be protected by a certificate of confidentiality automatically issued through the NIH. Confidentiality will be ensured by use of a unique numeric identification code and an acrostic that are unique to each study participant.

All research session data will be collected using standardized electronic forms on designed using the REDCap database hosted by Cambridge Health Alliance or through CAT-MH. REDCap (Research Electronic Data Capture) is a secure, web-based application designed to support data capture for research studies, providing an intuitive interface for validated data entry and export procedures to common statistical packages. All data management will be conducted by the research team operating from CHA. All data collection will take place under the supervision of the Dr. Schuman-Olivier, Dr.

Fulwiler or a research coordinator. Data will only be collected by members of the research team. Only CHA IRB-approved study staff will have access to the study REDCap databases. All REDCap access will be password-protected.

The name, birthday, and MRN will be entered into an initial CHA secured G-Suite database strictly for the purpose of screening and consent process. A unique numeric identifier and acrostic will be created for all participants who have completed an informed consent. The list linking any personal identifying information with the participant's study number and acrostic will be kept in a separate CHA secured G-suite file in a different folder. All data will be linked to these identifiers and no direct participant identifiers will be transmitted from REDCap and CAT-MH to STATA 14, R 3.5.3 or SAS 9.4 for analysis.

We either will generate medical record reports for participants who are in our study using the EHR system and then enter this information into REDCap OR enter EHR information directly into REDCap. This exported document will be kept in a CHA secure G-suite folder and will include MRN and name. In addition, the data analyst will use SQL to create data reports from EHR directly for analysis in STATA.

Database Protection:

Access to identifiable data will be limited to researchers directly involved in the study. All identifiable data will be destroyed 7 years after study completion and will not be maintained for future uses not specified in this research plan.

Minimization:
The risk of loss of confidentiality is judged to be minimal. Confidentiality will be maintained by disguising identifying information through the assignment of a numeric study number and an alphabetic study acrostic, and by keeping all data in the secure REDCap application. Any coded or de-identified data will be maintained in password-protected databases. Participant information will be accessible only to study staff. Information about study participants will not leave our institution in any form that would identify individual participants. Data will be transmitted to STATA 14 or SAS 9.4 in a pooled form with participants identified only by numeric code and acrostic. In addition, we will ask group participants to agree to a group confidentiality agreement. This agreement would require that information shared within the group remain solely within the group.

Confidentiality During Adverse Event (AE) Reporting
AE reports and annual summaries will not include participant- or group-identifiable material. Each report will only include the unique numeric identifier and acrostic.

11.4. Study Discontinuation

This study will be stopped prior to its completion if: (1) the intervention is associated with adverse effects that call into question the safety of the intervention; (2) difficulty in study recruitment or retention will significantly impact the ability to evaluate the study endpoints; (3) any new information becomes available during the trial that necessitates stopping the trial; or (4) other situations occur that might warrant stopping the trial.

During the funding of this study, any action by the IRB or one of the study investigators that results in a temporary or permanent suspension of the study will be reported to the NCCIH Program Official within 1 business day of notification.

12. COMMITTEES

The DSMB for this study includes Sarah Bowen, PhD, Linda Valeri, PhD, and Joji Suzuki, MD. See DSMP for details.

13. PUBLICATION OF RESEARCH FINDINGS

Any presentation, abstract, or manuscript will be made available for review by NCCIH prior to publication.

14. ADDENDUM: Enrolling Healthy Controls Participants for Sub-studies only

We will enroll up to 30 healthy control participants in the daily diary and salivary collection substudies. We will enroll healthy control participants both from CHAMindWell and from the local community. To recruit healthy controls for the sub-studies, CHAMindWell patients who have CAT-DI scores of ≤35 and who do not have any other CAT-MH screening test > 35 (or CAT-Anx > 50) in their most recent CAT-MH survey (does not include CAT-SDOH) may be referred to the study by CHAMindWell coordinators. We will recruit healthy controls for from the community by hanging the healthy control recruitment flyer in public settings with approval from that setting (Coffee shops, yoga studios, libraries, community boards, etc.) and by sending out the flyer electronically by email to CHA staff (i.e., research staff involved with the study will be excluded). After these healthy volunteer recruits will consent to the substudy, then they will participate in screening and baseline assessments as described above and will complete a full CAT-MH interview via REDCap (if not completed in past 30 days in MindWell already) and their eligibility will be reviewed. If eligible to continue, then they will complete the baseline CAT-Anx and CAT-DI, and then be asked to complete 7 days of daily diaries as described in the diary substudy. They will also be asked to come to CMC to provide a salivary sample as described above in the salivary substudy procedures.

Healthy Control Inclusion Criteria

All of the following are required criteria for inclusion in the study:

1. 18-70 years old
2. Sufficient English fluency to understand procedures and questionnaires
3. Ability to provide informed consent
4. Access to the internet and an electronic device to attend study groups and complete questionnaires.
5. Denies current depression

Healthy Control Exclusion Criteria

Any of the following is regarded as a criterion for exclusion from the study:

6. Active psychosis or severe level of psychosis on CAT-Psychosis (>35)
7. Bipolar I disorder history or severe level of mania on CAT-M/H[50] (>35)
8. Depression, indicated by CAT-DI > 35
9. Moderate to severe anxiety with CAT-Anx > 50
10. PTSD on CAT-PTSD (>35)
11. Current treatment with antipsychotic medication, mood stabilizer or benzodiazepines
12. Cognitive inability as demonstrated by the inability to complete an online informed consent assessment

13. Current participation in another experimental research study
14. Previous participation in an 8-week intensive Mindfulness-Based Intervention in past 1 year, including Mindful Mental Health Service (MMHS) groups (e.g., MTPC, MSC, MBSR)
15. Expected medical hospitalization in next 6 months
16. Expected incarceration in next 6 months
17. Severe substance use disorder or high risk on CAT-MH SUD. In addition, use of cocaine, unprescribed opioids, stimulants, or benzodiazepines in the past 3 months.
18. Inability to complete screening, baseline assessments and 5 daily diaries.

15. REFERENCES

1. Arroll B, Moir F, Kendrick T. Effective management of depression in primary care: a review of the literature. BJGP open 2017;1(2).
2. Chiang JJ, Turiano NA, Mroczek DK, Miller GE. Affective reactivity to daily stress and 20-year mortality risk in adults with chronic illness: Findings from the National Study of Daily Experiences. Health Psychol 2018;37(2):170–8.
3. Sobell LC, Brown J, Leo GI, Sobell MB. The reliability of the Alcohol Timeline Followback when administered by telephone and by computer. Drug Alcohol Depend [Internet] 1996;42(1):49–54. Available from: http://www.sciencedirect.com/science/article/pii/037687169601263X
4. Kessler RC, Andrews G, Colpe LJ, et al. Short screening scales to monitor population prevalences and trends in non-specific psychological distress. Psychol Med 2002;32(6):959–76.
5. Webb CA, Rosso IM, Rauch SL. Internet-based cognitive-behavioral therapy for depression: Current progress and future directions. Harv. Rev. Psychiatry. 2017;
6. Griffiths KM, Christensen H, Jorm AF, Evans K, Groves C. Effect of web-based depression literacy and cognitive-behavioural therapy interventions on stigmatising attitudes to depression: randomised controlled trial. Br J Psychiatry 2004;185:342–9.
7. Pfefferbaum B, North CS. Mental Health and the Covid-19 Pandemic. N Engl J Med 2020;
8. Chen Q, Liang M, Li Y, et al. Mental health care for medical staff in China during the COVID-19 outbreak. The Lancet Psychiatry [Internet] 2020 [cited 2020 Apr 29];7(4):e15–6. Available from: https://www-ncbi-nlm-nih-gov.ezproxy.umassmed.edu/pmc/articles/PMC7129426/
9. Galea S, Merchant RM, Lurie N. The Mental Health Consequences of COVID-19 and Physical Distancing: The Need for Prevention and Early Intervention. JAMA Intern Med [Internet] 2020 [cited 2020 Apr 20];Available from: https://jamanetwork.com/journals/jamainternalmedicine/fullarticle/2764404
10. Kang L, Li Y, Hu S, et al. The mental health of medical workers in Wuhan, China dealing with the 2019 novel coronavirus. The Lancet Psychiatry. 2020;7(3):e14.
11. Rajkumar RP. COVID-19 and mental health: A review of the existing literature. Asian J Psychiatr 2020;52:102066.
12. Galea S, Brewin CR, Gruber M, et al. Exposure to hurricane-related stressors and mental

illness after Hurricane Katrina. Arch Gen Psychiatry 2007;64(12):1427–34.
13. Rhodes J, Chan C, Paxson C, Rouse CE, Waters M, Fussell E. The Impact of Hurricane Katrina on the Mental and Physical Health of Low-Income Parents in New Orleans. Am J Orthopsychiatry 2010;80(2):237–47.
14. Berggren RE, Curiel TJ. After the storm--health care infrastructure in post-Katrina New Orleans. N Engl J Med 2006;354(15):1549–52.
15. Wang PS, Gruber MJ, Powers RE, et al. Disruption of existing mental health treatments and failure to initiate new treatment after Hurricane Katrina. Am J Psychiatry 2008;165(1):34–41.
16. Wang PS, Gruber MJ, Powers RE, et al. Mental Health Service Use Among Hurricane Katrina Survivors in the Eight Months After the Disaster. Psychiatr Serv [Internet] 2007 [cited 2020 Apr 26];58(11):1403–11. Available from: http://psychiatryonline.org/doi/abs/10.1176/ps.2007.58.11.1403
17. Moise IK, Ruiz MO. Hospitalizations for Substance Abuse Disorders Before and After Hurricane Katrina: Spatial Clustering and Area-Level Predictors, New Orleans, 2004 and 2008. Prev Chronic Dis 2016;13:E145.
18. Cepeda A, Valdez A, Kaplan C, Hill LE. Patterns of substance use among hurricane Katrina evacuees in Houston, Texas. Disasters 2010;34(2):426–46.
19. Shuler MN, Wallington SF, Qualls-Hampton RY, Podesta AE, Suzuki S. Trend analysis of substance abuse treatment admissions in New Orleans from 2000–2012: A population-based comparison pre- and post-Hurricane Katrina. Subst Use Misuse 2016;51(12):1542–54.
20. Phillippi SW, Beiter K, Thomas CL, et al. Medicaid Utilization Before and After a Natural Disaster in the 2016 Baton Rouge-Area Flood. Am J Public Health 2019;109(S4):S316–21.
21. Joseph NT, Matthews KA, Myers HF. Conceptualizing health consequences of Hurricane Katrina from the perspective of socioeconomic status decline. Health Psychol 2014;33(2):139–46.
22. Southwick SM, Vythilingam M, Charney DS. The psychobiology of depression and resilience to stress: implications for prevention and treatment. Annu Rev Clin Psychol 2005;1:255–91.
23. Russo SJ, Murrough JW, Han M-H, Charney DS, Nestler EJ. Neurobiology of resilience. Nat Neurosci 2012;15(11):1475–84.
24. Charles ST, Piazza JR, Mogle J, Sliwinski MJ, Almeida DM. The wear and tear of daily stressors on mental health. Psychol Sci 2013;24(5):733–41.
25. Smith K. Mental health: a world of depression. Nature [Internet] 2014 [cited 2020 May 9];515(7526):181. Available from: http://www.nature.com/articles/515180a
26. Liu Q, He H, Yang J, Feng X, Zhao F, Lyu J. Changes in the global burden of depression from 1990 to 2017: Findings from the Global Burden of Disease study. J Psychiatr Res [Internet] 2019 [cited 2020 May 9];Available from: https://linkinghub.elsevier.com/retrieve/pii/S0022395619307381
27. Patten SB, Williams JVA, Lavorato DH, Bulloch AGM, MacQueen G. Depressive episode characteristics and subsequent recurrence risk. J Affect Disord 2012;
28. Conradi HJ, Ormel J, de Jonge P. Presence of individual (residual) symptoms during depressive episodes and periods of remission: a 3-year prospective study. Psychol Med

[Internet] 2011 [cited 2020 May 9];41(6):1165–74. Available from: https://www.cambridge.org/core/product/identifier/S0033291710001911/type/journal_article

29. Segal Williams, JMG, Teasdale, JD Z V. Mindfulness-Based Cognitive Therapy for Depression Second Edition. Second. New York, NY: The Guilford Press; 2012.
30. Teasdale JD, Segal Z V, Williams JM, Ridgeway VA, Soulsby JM, Lau MA. Prevention of relapse/recurrence in major depression by mindfulness-based cognitive therapy. 2000.
31. Ma SH, Teasdale JD. Mindfulness-based cognitive therapy for depression: replication and exploration of differential relapse prevention effects. 2004.
32. Piet J, Hougaard E. The effect of mindfulness-based cognitive therapy for prevention of relapse in recurrent major depressive disorder: A systematic review and meta-analysis. Clin. Psychol. Rev. 2011;31:1032–40.
33. Segal Z V, Bieling P, Young T, et al. Antidepressant monotherapy vs sequential pharmacotherapy and mindfulness-based cognitive therapy, or placebo, for relapse prophylaxis in recurrent depression. Arch Gen Psychiatry 2010;67:1256–64.
34. Kuyken W, Hayes R, Barrett B, et al. The effectiveness and cost-effectiveness of mindfulness-based cognitive therapy compared with maintenance antidepressant treatment in the prevention of depressive relapse/recurrence: results of a randomised controlled trial (the PREVENT study). Health Technol Assess 2015;19(73):1–124.
35. Kuyken W, Warren FC, Taylor RS, et al. Efficacy of Mindfulness-Based Cognitive Therapy in Prevention of Depressive Relapse. JAMA Psychiatry [Internet] 2016;73(6):565. Available from: https://archpsyc.jamanetwork.com/article.aspx?articleid=2517515 England%5Cnhttp://archpsyc.jamanetwork.com/article.aspx?doi=10.1001/jamapsychiatry.2016.0076
36. van Aalderen JR, Donders ART, Giommi F, Spinhoven P, Barendregt HP, Speckens AEM. The efficacy of mindfulness-based cognitive therapy in recurrent depressed patients with and without a current depressive episode: a randomized controlled trial. Psychol Med [Internet] 2012;42(05):989–1001. Available from: http://dx.doi.org/10.1017/S0033291711002054
37. Chiesa A, Castagner V, Andrisano C, et al. Mindfulness-based cognitive therapy vs. psycho-education for patients with major depression who did not achieve remission following antidepressant treatment. Psychiatry Res [Internet] 2015;226(2–3):474–83. Available from: http://www.sciencedirect.com/science/article/pii/S0165178115000736
38. Eisendrath SJ, Gillung E, Delucchi KL, et al. A Randomized Controlled Trial of Mindfulness-Based Cognitive Therapy for Treatment-Resistant Depression. Psychother Psychosom [Internet] 2016;85(2):99–110. Available from: http://www.ncbi.nlm.nih.gov/pubmed/26808973
39. Goldberg SB, Tucker RP, Greene PA, Davidson RJ, Kearney DJ, Simpson TL. Mindfulness-based cognitive therapy for the treatment of current depressive symptoms: a meta-analysis. Cogn Behav Ther [Internet] 2019 [cited 2019 Apr 5];48(6):445–62. Available from: http://www.ncbi.nlm.nih.gov/pubmed/30732534
40. Strauss C, Cavanagh K, Oliver A, Pettman D. Mindfulness-based interventions for people diagnosed with a current episode of an anxiety or depressive disorder: A meta-analysis of randomised controlled trials. PLoS One [Internet] 2014 [cited 2017 May 3];9(4):e96110.

Available from: http://dx.plos.org/10.1371/journal.pone.0096110
41. Panahi F, Faramarzi M. The Effects of Mindfulness-Based Cognitive Therapy on Depression and Anxiety in Women with Premenstrual Syndrome. Depress Res Treat 2016;2016.
42. Segal Z V., Dimidjian S, Beck A, et al. Outcomes of Online Mindfulness-Based Cognitive Therapy for Patients with Residual Depressive Symptoms: A Randomized Clinical Trial. JAMA Psychiatry 2020;
43. Lever Taylor B, Cavanagh K, Strauss C. The Effectiveness of Mindfulness-Based Interventions in the Perinatal Period: A Systematic Review and Meta-Analysis. PLoS One [Internet] 2016 [cited 2017 Oct 6];11(5):e0155720. Available from: http://www.ncbi.nlm.nih.gov/pubmed/27182732
44. Mohamadi J, Ghazanfari F, Drikvand FM. Comparison of the Effect of Dialectical Behavior Therapy, Mindfulness Based Cognitive Therapy and Positive Psychotherapy on Perceived Stress and Quality of Life in Patients with Irritable Bowel Syndrome: a Pilot Randomized Controlled Trial. Psychiatr Q 2019;90(3):565–78.
45. Dimidjian S, Beck A, Felder JN, Boggs JM, Gallop R, Segal Z V. Web-based Mindfulness-based Cognitive Therapy for reducing residual depressive symptoms: An open trial and quasi-experimental comparison to propensity score matched controls. Behav Res Ther 2014;
46. Teasdale JD. Cognitive Vulnerability to Persistent Depression. Cogn Emot [Internet] 1988;2(3):247–74. Available from: http://www.informaworld.com/10.1080/02699938808410927
47. Segal Z V, Kennedy S, Gemar M, Hood K, Pedersen R, Buis T. Cognitive reactivity to sad mood provocation and the prediction of depressive relapse. Arch Gen Psychiatry [Internet] 2006;63(7):749–55. Available from: http://www.ncbi.nlm.nih.gov/entrez/query.fcgi?cmd=Retrieve&db=PubMed&dopt=Citation&list_uids=16818864
48. Moulds ML, Kandris E, Williams AD, Lang T, Yap C, Hoffmeister K. An Investigation of the Relationship Between Cognitive Reactivity and Rumination. Behav Ther [Internet] 2008 [cited 2020 May 9];39(1):65–71. Available from: https://linkinghub.elsevier.com/retrieve/pii/S0005789407000585
49. Gu J, Strauss C, Bond R, Cavanagh K. How do mindfulness-based cognitive therapy and mindfulness-based stress reduction improve mental health and wellbeing? A systematic review and meta-analysis of mediation studies. Clin Psychol Rev [Internet] 2015;37:1–12. Available from: http://www.ncbi.nlm.nih.gov/pubmed/25689576
50. Achtyes ED, Halstead S, Smart LA, et al. Validation of computerized adaptive testing in an outpatient nonacademic setting: The VOCATIONS trial. Psychiatr Serv 2015;66(10):1091–6.
51. Gibbons RD, Kupfer D, Frank E, Moore T, Beiser DG, Boudreaux ED. Development of a computerized adaptive test suicide scale—The CAT-SS. J Clin Psychiatry 2017;78(9):1376–82.
52. Gibbons RD, Weiss DJ, Pilkonis PA, et al. Development of a computerized adaptive test for depression. Arch Gen Psychiatry 2012;69(11):1104–12.
53. Gibbons RD, Hooker G, Finkelman MD, et al. The computerized adaptive diagnostic test for major depressive disorder (CAD-MDD): A screening tool for depression. J Clin

Psychiatry 2013;74(7):669–74.
54. Twomey C, O'Reilly G. Effectiveness of a freely available computerised cognitive behavioural therapy programme (MoodGYM) for depression: Meta-analysis. Aust N Z J Psychiatry 2017;51(3):260–9.
55. Farrer L, Christensen H, Griffiths KM, Mackinnon A. Web-Based Cognitive Behavior Therapy for Depression With and Without Telephone Tracking in a National Helpline: Secondary Outcomes From a Randomized Controlled Trial. J Med Internet Res 2012;14(3):e68.
56. Guille C, Zhao Z, Krystal J, Nichols B, Brady K, Sen S. Web-Based Cognitive Behavioral Therapy Intervention for the Prevention of Suicidal Ideation in Medical Interns: A Randomized Clinical Trial. JAMA psychiatry 2015;72(12):1192–8.
57. Gibbons RD, Weiss DJ, Frank E, Kupfer D. Computerized Adaptive Diagnosis and Testing of Mental Health Disorders. Annu Rev Clin Psychol 2016;12(1):83–104.
58. Gibbons RD, deGruy F V. Without Wasting a Word: Extreme Improvements in Efficiency and Accuracy Using Computerized Adaptive Testing for Mental Health Disorders (CAT-MH). Curr. Psychiatry Rep. 2019;
59. Crane RS, Eames C, Kuyken W, et al. Development and validation of the mindfulness-based interventions - teaching assessment criteria (MBI:TAC). Assessment 2013;20(6):681–8.
60. Black DS. Mindfulness-based interventions: an antidote to suffering in the context of substance use, misuse, and addiction. Subst Use Misuse 2014;49(5):487–91.
61. Lindahl JR, Fisher NE, Cooper DJ, Rosen RK, Britton WB. The varieties of contemplative experience: A mixed-methods study of meditation-related challenges in Western Buddhists [Internet]. 2017. Available from: http://dx.doi.org/10.1371/journal.pone.0176239
62. Posner K, Brown GK, Stanley B, et al. The Columbia-suicide severity rating scale: Initial validity and internal consistency findings from three multisite studies with adolescents and adults. Am J Psychiatry 2011;168(12):1266–77.
63. Richards D, Richardson T. Computer-based psychological treatments for depression: a systematic review and meta-analysis. Clin Psychol Rev 2012;32(4):329–42.
64. Magnusson K. Power Analysis for Longitudinal Multilevel Models [Internet]. 2018;Version 0. Available from: https://github.com/rpsychologist/powerlmm
65. Efird J. Blocked Randomization with Randomly Selected Block Sizes. Int. J. Environ. Res. Public Heal. . 2011;8(1).
66. Rabe-Hesketh S, Skrondal A. Multilevel and longitudinal modeling using Stata: Vol I: Continuous Responses & II: Categorical responses, counts, and survival. 3rd ed. Stata Press; 2012.
67. Deb P, Norton EC, Graham Manning W. Health econometrics using Stata. College Station, TX: Stata Press; 2017.
68. Mroczek DK, Kolarz CM. The effect of age on positive and negative affect: A developmental perspective on happiness. J. Pers. Soc. Psychol. 1998;75(5):1333–49.
69. Piazza JR, Charles ST, Sliwinski MJ, Mogle J, Almeida DM. Affective reactivity to daily stressors and long-term risk of reporting a chronic physical health condition. Ann Behav Med 2013;45(1):110–20.
70. Bolger N, DeLongis A, Kessler RC, Schilling EA. Effects of daily stress on negative

mood. J Pers Soc Psychol 1989;57(5):808–18.
71. Cohen LH, Gunthert KC, Butler AC, O'Neill SC, Tolpin LH. Daily affective reactivity as a prospective predictor of depressive symptoms. J Pers 2005;73(6):1687–713.
72. Charles ST, Piazza JR, Mogle J, Sliwinski MJ, Almeida DM. The wear and tear of daily stressors on mental health. Psychol Sci [Internet] 2013;24(5):733–41. Available from: https://pubmed.ncbi.nlm.nih.gov/23531486
73. Almeida DM, Charles ST, Mogle J, et al. Charting adult development through (historically changing) daily stress processes. Am Psychol 2020;75(4):511–24.
74. Almeida DM, Horn MC. Is Daily Life More Stressful during Middle Adulthood? In: How healthy are we?: A national study of well-being at midlife. Chicago, IL, US: University of Chicago Press; 2004. p. 425–51.
75. Preacher KJ, Zyphur MJ, Zhang Z. A general multilevel SEM framework for assessing multilevel mediation. Psychol Methods 2010;15(3):209–33.
76. Benjamini Y, Hochberg Y. Controlling the False Discovery Rate: A Practical and Powerful Approach to Multiple Testing. J R Stat Soc Ser B [Internet] 1995;57(1):289–300. Available from: http://www.jstor.org/stable/2346101
77. Cao J, Zhang S. Multiple comparison procedures. JAMA 2014;312(5):543–4.
78. Glickman ME, Rao SR, Schultz MR. False discovery rate control is a recommended alternative to Bonferroni-type adjustments in health studies. J Clin Epidemiol 2014;67(8):850–7.
79. Wahbeh H, Goodrich E, Goy E, Oken BS. Mechanistic Pathways of Mindfulness Meditation in Combat Veterans With Posttraumatic Stress Disorder. J Clin Psychol 2016;72(4):365–83.
80. Gawande R, To MN, Pine E, et al. Mindfulness Training Enhances Self-Regulation and Facilitates Health Behavior Change for Primary Care Patients: a Randomized Controlled Trial. J Gen Intern Med 2019;34(2).
81. Sobell LC, Brown J, Leo GI, Sobell MB. The reliability of the Alcohol Timeline Followback when administered by telephone and by computer. Drug Alcohol Depend 1996;42(1):49–54.
82. Gámez W, Chmielewski M, Kotov R, Ruggero C, Suzuki N, Watson D. The brief experiential avoidance questionnaire: Development and initial validation. Psychol Assess 2014;26(1):35–45.
83. Gratz K, Roemer L. Multidimensional Assessment of Emotion Regulation and Dysregulation: Development, Factor Structure, and Initial Validation of the Difficulties in Emotion Regulation Scale. J Psychopathol Behav Assess 2004;26:41–54.
84. Cohen S, Kamarck T, Mermelstein R. A global measure of perceived stress. J Health Soc Behav 1983;24(4):385–96.
85. Raes F, Pommier E, Neff KD, Van Gucht D. Construction and factorial validation of a short form of the Self-Compassion Scale. Clin Psychol Psychother 2011;18(3):250–5.
86. Bornemann B, Singer T. Taking time to feel our body: Steady increases in heartbeat perception accuracy and decreases in alexithymia over 9 months of contemplative mental training. Psychophysiology 2017;54(3):469–82.
87. Fresco DM, Moore MT, van Dulmen MHM, et al. Initial Psychometric Properties of the Experiences Questionnaire: Validation of a Self-Report Measure of Decentering. Behav Ther 2007;38(3):234–46.

88. Soler J, Franquesa A, Feliu-Soler A, et al. Assessing Decentering: Validation, Psychometric Properties, and Clinical Usefulness of the Experiences Questionnaire in a Spanish Sample. Behav Ther 2014;45(6):863–71.
89. Beck AT, Steer RA, Brown GK. Manual for the Beck depression inventory-II. San Antonio, TX Psychol Corp 1996;
90. Dozois DJA, Covin R. The Beck Depression Inventory-II (BDI-II), Beck Hopelessness Scale (BHS), and Beck Scale for Suicide Ideation (BSS) [Internet]. In: Comprehensive handbook of psychological assessment Vol 2 Personality assessment. 2004. p. 50–69.Available from: http://0-search.ebscohost.com.mercury.concordia.ca/login.aspx?direct=true&db=psyh&AN=2004-12821-005&site=ehost-live
91. Almeida DM, Wethington E, Kessler RC. The Daily Inventory of Stressful Events: An interview-based approach for measuring daily stressors. Assessment 2002;9(1):41–55.
92. Mroczek DK, Kolarz CM. The effect of age on positive and negative affect: a developmental perspective on happiness. J Pers Soc Psychol 1998;75(5):1333–49.
93. Premium Oral Fluid Drug Testing [Internet]. CLIA Waiv. Available from: https://www.cliawaived.com/amfile/file/download/file/1164/product/6442/
94. Devilly GJ, Borkovec TD. Psychometric properties of the credibility/expectancy questionnaire. J Behav Ther Exp Psychiatry 2000;31(2):73–86.
95. College VUP. Responses to Stress Questionnaire (RSQ). 2020;
96. Riis J, Bryce C, Stebbins J, Granger D. Salivary total Immunoglobulin G as a surrogate marker of oral immune activity in salivary bioscience research. Brain Behav Immun 2019;1.
97. World Health Organization. Oral health surveys: basic methods. 5th editio. World Health Organization; 2013.
98. Ahorsu DK, Lin C-Y, Imani V, Saffari M, Griffiths MD, Pakpour AH. The Fear of COVID-19 Scale: Development and Initial Validation. Int J Ment Health Addict [Internet] 2020;1–9. Available from: https://pubmed.ncbi.nlm.nih.gov/32226353
99. Carmody J, Baer RA. Relationships between mindfulness practice and levels of mindfulness, medical and psychological symptoms and well-being in a mindfulness-based stress reduction program. J Behav Med 2008;31(1):23–33.
100. Hawley LL, Rector NA, Laposa JM. Examining the dynamic relationships between exposure tasks and cognitive restructuring in CBT for SAD: Outcomes and moderating influences. J Anxiety Disord 2016;
101. Hawley LL, Padesky CA, Hollon SD, et al. Cognitive-Behavioral Therapy for Depression Using Mind Over Mood: CBT Skill Use and Differential Symptom Alleviation. Behav Ther 2017;48(1):29–44.
