# Supplementary material for: Effects of Remotely Delivered and Web-Based Interventions on Depression Severity During the COVID-19 Pandemic: 3-Arm Randomized Controlled Trial
Source: JMIR Ment Health. 2026 Aug 4;13:e88388. doi: 10.2196/88388 (PMC13436849; doi:10.2196/88388)
Supplement: Supplementary file 2 [file mental-v13-e88388-s001.docx]

**Multimedia Appendix 1**

**Table S1.** MBCT and MBCT-R session outlines.

|  | **MBCT^a^** | | **MBCT-R^b^** | |
| --- | --- | --- | --- | --- |
| **Week** | **Title and Themes** | **Content** | **Title and Themes** | **Content** |
| **1** | Awareness & Automatic Pilot  ·Mindfulness and automaticity  ·Enhancing attention to the senses and sensations  ·Learning to place, hold, and shift attention | ·Raisin exercise  ·Body scan  ·Mindful inquiry  ·Short breath focus | Awareness & Automatic Pilot  ·Mind-wandering  ·Habituated behaviors  ·Present moment awareness | ·Raisin exercise  ·Body scan  ·Mindful inquiry  ·Short breath focus |
| **2** | Living in Our Heads  ·Normalizing obstacles to paying attention to the present  ·Reality as a construct or interpretation  ·Focused attention on breath/body | ·Body scan  ·Inquiry and review  ·Thoughts and feelings exercise  ·Pleasant Events Calendar  ·Sitting meditation (breath/body) | Living in Our Heads  ·The wandering mind  ·Obstacles to paying attention  ·The breath as an anchor | ·Body scan  ·Inquiry & practice review  ·Thoughts and feelings exercise  ·Brief sitting meditation (breath, body) |
| **3** | Gathering the Scattered Mind  ·Normalizing “distracted mind”  ·Focusing on breath/body as attentional anchors  ·Deconstructing experience into components | ·Sitting meditation (sight/breath/body)  ·3-minute breathing space  ·Mindful movement  ·Unpleasant events calendar  ·Inquiry and review | Gathering the Scattered Mind  ·Lost in thought  ·Welcoming & curious attitude  ·Bringing mindfulness to daily life | ·Sitting (breath, body, sensations)  ·Mindful movement  ·3-minute breathing space  ·Inquiry & practice review |
| **4** | Recognizing Aversion  ·Moving from focused to open monitoring of experience  ·Staying present with difficulty  ·Disrupting rumination by shifting attention to body | ·Sitting meditation (sound/breath/body)  ·Inquiry and review  ·Automatic Thoughts questionnaire  ·3-minute breathing space responsive  ·Mindful walking | Recognizing Aversion  ·Avoidance and attachment  ·Turning towards difficulty  ·A different place to view thoughts | ·Sitting (breath, body, sounds, thoughts)  ·Automatic Thoughts Questionnaire  ·3-minute breathing space responsive  ·Mindful walking  ·Inquiry & practice review |
| **5** | Allowing and Letting Be  ·Being mode of mind  ·Acceptance  ·Choosing skillful responses  ·Moving towards self-care | ·Sitting meditation working with difficulty  ·Inquiry and review  ·3-minute breathing space responsive | Allowing and Letting Be  ·A different relationship to the unwanted  ·Acceptance; Window of tolerance  ·Moving towards self-care | ·Sitting meditation focus on difficulty  ·Inquiry & practice review  ·Working with difficulty  ·Breathing space |
| **6** | Thoughts are Not Facts  ·Thoughts as mental events  ·Interpretations dependent on state and context  ·Early warning signs | ·Sitting meditation working with thoughts  ·Inquiry and review  ·Alternative viewpoints exercise  ·3-minute breathing space  ·Discuss relapse signature | Thoughts are Not Facts  ·How moods influence thoughts  ·Thoughts as mental events  ·Early warning signs of emotional distress | ·Sitting meditation focus on thoughts  ·Moods & thoughts  · Alternative viewpoints  ·Discuss relapse signature  ·Discuss retreat |
| **6.5**  **Half-**  **Day**  **Retreat** | Making the Practice Your Own  ·Being in silence  ·Going deeper with mindfulness  ·Strengthening the practice | ·Mindful walking  ·Loving kindness meditation  ·Discussion of practices  ·Discuss preparation for end of course | Making the Practice Your Own  ·Being in silence  ·Going deeper with mindfulness  ·Strengthening the practice | ·Mindful walking  ·Loving kindness meditation  ·Discussion of practices  ·Discuss preparation for end of course |
| **7** | How Can I Best Take Care of Myself?  ·Self-care for managing mental wellness  ·Behavior and mood are interrelated  ·Skills to prevent relapse | ·Sitting meditation (all)  ·Inquiry and review  ·Nourishing and depleting activities  ·3-minute breathing space  ·Discuss action plans for relapse prevention | How Can I Best Take Care of Myself?  ·Behavior and mood are interrelated  ·Skills to prevent relapse  ·Fostering resilience | ·Sitting meditation (all)  ·Nourishing & depleting exercise - links between activity and mood  ·Scheduling activities and mindful action  ·Action plan for staying well |
| **8** | Maintaining & Extending New Learning  ·Sustaining mindfulness practices to support working with difficulty  ·The importance of self-care | ·Body scan  ·Inquiry and review  ·Review action plans  ·Review course  ·Closing practice | Maintaining & Extending New Learning  ·Ending and beginnings  ·The importance of self-care  ·Well-being and resilience  ·What is most important to us | ·Body scan  ·Review action plans  ·Review of entire course  ·Keeping practice going  ·Closing exercise |

^a^MBCT = Mindfulness-Based Cognitive Therapy ^b^MBCT-R = Mindfulness-Based Cognitive Therapy for Resilience

**Table S2.** Three-way exploratory analysis of study arm by week for primary (1^o^), secondary (2^o^), and exploratory outcome analyses.

| **Outcome** | **Results** | |
| --- | --- | --- |
| **Group** | **Study Arm by Week** | |
|  | **Chi-sq, df** | ***P Value*** |
| **Depression** | | |
| CHA-MW^a^ | 12.3, 12 | .423 |
| 1^0:^ MBCT-R^b^ |  |  |
| 2^0^: iCBT^c^ |  |  |
| **Anxiety** | | |
| CHA-MW | 13.1, 12 | .364 |
| MBCT-R |  |  |
| iCBT |  |  |
| **Perceived Stress** | | |
| CHA-MW | 7.3, 8 | .504 |
| MBCT-R |  |  |
| iCBT |  |  |
| **Interoception** | | |
| CHA-MW | 7.1, 6 | .315 |
| MBCT-R |  |  |
| iCBT |  |  |
| **Experiential Avoidance** | | |
| CHA-MW | 11.4, 6 | .077 |
| MBCT-R |  |  |
| iCBT |  |  |
| **Emotion Dysregulation** | | |
| CHA-MW | 3.9, 6 | .697 |
| MBCT-R |  |  |
| iCBT |  |  |
| **Self-Compassion** | | |
| CHA-MW | 4.3, 6 | .631 |
| MBCT-R |  |  |
| iCBT |  |  |
| **Decentering** | | |
| CHA-MW | 10.1, 6 | .119 |
| MBCT-R |  |  |
| iCBT |  |  |

^a^CHA-MW = Cambridge Health Alliance (CHA) Mindwell
^b^MBCT-R = Mindfulness-Based Cognitive Therapy for Resilience ^c^iCBT = internet Cognitive Behavioral Therapy

**Table S3.** Percentage of mental health clinician visit use by study arm.

|  |  | CHA-MW^a^ (n=19) |  | MBCT-R^b^ (n=37) |  | iCBT^c^ (n=41) |  | Total (n=97) |
| --- | --- | --- | --- | --- | --- | --- | --- | --- |
|  |  | At Least 1 (%) |  | At Least 1 (%) |  | At Least 1 (%) |  | At Least 1 (%) |
| Total visits during 24 weeks |  | 63.2% |  | 67.6% |  | 51.2% |  | 59.8% |
| Telephone Visits |  | 57.9% |  | 45.9% |  | 46.3% |  | 48.5% |
| Psychotherapy |  | 15.8% |  | 24.3% |  | 17.1% |  | 19.6% |
| Psychopharmacology |  | 21.1% |  | 24.3% |  | 2.4% |  | 14.4% |
| Group |  | 0% |  | 2.7% |  | 9.8% |  | 5.2% |

^a^CHA-MW = Cambridge Health Alliance (CHA) ^b^MBCT-R = Mindfulness-Based Cognitive Therapy for Resilience Mindwell ^c^iCBT = internet Cognitive Behavioral Therapy

Table shows that when visits were disaggregated by type, findings revealed that patients in the MBCT-R study arm were more likely to be engaged in psychopharmacology visits than those in the iCBT study arm (difference = 21.9%, 95% CI [5.9%, 36.1%], *P*=.005) but not those in the control CHA-MW alone arm.

**Table S4.** Percentage of individual psychopharmacology visit use before and during intervention by study arm.

|  |  | Psychopharmacology 24-wk Before | | |  | Psychopharmacology 24-wk During | | |
| --- | --- | --- | --- | --- | --- | --- | --- | --- |
|  |  | Mean | SD | % >=1 |  | Mean | SD | % >=1 |
| CHA-MW |  | 0.00 | 0.00 | 0% |  | 0.53 | 1.31 | 21% |
| MBCT-R |  | 0.57 | 1.56 | 14% |  | 0.95 | 1.88 | 24% |
| iCBT |  | 0.24 | 1.14 | 7% |  | 0.05 | 0.31 | 2% |

**Table S5.** Sensitivity analysis pre versus post differences between participants who did and did not use psychopharmacology by study group.

| Outcome Measure | (Week 24 v Baseline) By (Use of Psychopharmacology ) By | | | | |
| --- | --- | --- | --- | --- | --- |
|  | (MBCT-R^a^ v. CHA-MW^b^ (Control)) | |  | (iCBT^c^ v. CHA-MW (Control)) | |
|  | Chi-Sq, df | *P* Value |  | Chi-Sq, df | *P* Value |
| Depression | 0.49, 1 | .483 |  | 0.04, 1 | .838 |
| Anxiety | 3.28, 1 | .070 |  | 0.01, 1 | .942 |
| Perceived Stress | 0.16, 1 | .689 |  | 2.66, 1 | .103 |
| Interoceptive Awareness | 0.2, 1 | .657 |  | 0, 1 | .963 |
| Experiential Avoidance | 1.5, 1 | .220 |  | 0.44, 1 | .505 |
| Emotion Dysregulation | 2.23, 1 | .136 |  | 2.97, 1 | .085 |
| Self-Compassion | 1.9, 1 | .168 |  | 0.42, 1 | .517 |
| Decentering | 0, 1 | .996 |  | 0.54, 1 | .461 |

^a^MBCT-R = Mindfulness-Based Cognitive Therapy for Resilience ^b^CHA-MW = Cambridge Health Alliance (CHA) Mindwell ^c^iCBT = internet Cognitive Behavioral Therapy

Table shows the Chi-Sq (df) and p-value associated with the interaction term used to assess whether the difference difference between baseline and 24 weeks differed by those who did and did not use Psychopharmacology between MBCT-R and CHA-MW Control and iCBT and CHA-MW Control.

**Table S6.** Exploratory outcome scores by study arm and week for primary (1^o^) and secondary (2^o^) outcome analyses.

|  |  | **1^o^: MBCT-R^a^** | | **2^o^: iCBT^b^** | | **CHA-MW^c^** | | **Total** | |
| --- | --- | --- | --- | --- | --- | --- | --- | --- | --- |
| **Week** |  | **Mean (SD)** | **n** | **Mean (SD)** | **n** | **Mean (SD)** | **n** | **Mean (SD)** |  |
| **Anxiety** |  |  |  |  |  |  |  |  |  |
| 0 |  | 45.5 (13.6) | 37 | 41.1 (18.4) | 41 | 46 (14) | 18 | 43.7 (15.9) | 96 |
| 4 |  | 37.9 (17.7) | 28 | 28.7 (13.7) | 35 | 36.3 (16) | 15 | 33.5 (16.1) | 78 |
| 8 |  | 32.2 (18.6) | 30 | 26.1 (19.1) | 31 | 31.8 (14.3) | 15 | 29.6 (18.1) | 76 |
| 12 |  | 34.2 (19.2) | 28 | 27.2 (17.4) | 28 | 31.3 (12) | 14 | 30.8 (17.3) | 70 |
| 16 |  | 30.6 (20.4) | 23 | 27.4 (23) | 29 | 34.4 (14.6) | 15 | 30.1 (20.4) | 67 |
| 20 |  | 33.1 (21.3) | 20 | 31.5 (16.3) | 27 | 29.4 (12.7) | 14 | 31.6 (17.2) | 61 |
| 24 |  | 29.6 (20.7) | 23 | 28.1 (18.5) | 29 | 29.6 (14) | 15 | 28.9 (18.2) | 67 |
| **Perceived Stress** | | | |  | |  | |  | |
| 0 |  | 40.4 (5.9) | 36 | 37.1 (7.4) | 41 | 39.8 (7) | 19 | 38.9 (6.9) | 96 |
| 4 |  | 38.5 (5.5) | 19 | 34.1 (7) | 33 | 36 (5.9) | 14 | 35.8 (6.5) | 66 |
| 8 |  | 35.3 (8.5) | 26 | 33.4 (7.7) | 27 | 32.8 (8) | 12 | 34.1 (8) | 65 |
| 12 |  | 35.3 (10.2) | 20 | 34.9 (8.4) | 25 | 34.3 (6) | 12 | 34.9 (8.6) | 57 |
| 24 |  | 34 (8.8) | 20 | 31.1 (10) | 26 | 33.4 (7.6) | 11 | 32.5 (9.1) | 57 |
| **Interoception** | | | |  | |  | |  | |
| 0 |  | 2.3 (0.7) | 36 | 2.5 (0.7) | 41 | 2.2 (0.6) | 19 | 2.4 (0.7) | 96 |
| 4 |  | 2.7 (0.8) | 19 | 2.7 (0.8) | 33 | 2.6 (0.7) | 14 | 2.7 (0.7) | 66 |
| 8 |  | 2.9 (0.8) | 26 | 2.7 (0.7) | 27 | 2.5 (0.8) | 12 | 2.7 (0.7) | 65 |
| 12 |  | 2.9 (0.9) | 20 | 2.6 (0.6) | 24 | 2.6 (0.7) | 11 | 2.7 (0.7) | 55 |
| **Experiential Avoidance** | | | |  | |  | |  | |
| 0 |  | 50.7 (9.1) | 36 | 51.2 (13.5) | 41 | 52.7 (11.4) | 19 | 51.3 (11.5) | 96 |
| 4 |  | 45.9 (10.2) | 19 | 48.2 (13.3) | 33 | 43.7 (13.2) | 14 | 46.6 (12.4) | 66 |
| 8 |  | 42.5 (10.7) | 26 | 48.1 (12.5) | 28 | 48.9 (11.6) | 12 | 46.1 (11.9) | 66 |
| 12 |  | 43.7 (13) | 21 | 48 (12.2) | 26 | 48.5 (11.2) | 12 | 46.6 (12.3) | 59 |
| **Emotion Dysregulation** | | | |  | |  | |  | |
| 0 |  | 94.6 (18.8) | 36 | 95.2 (23.6) | 41 | 103.9 (22.3) | 19 | 96.7 (21.7) | 96 |
| 4 |  | 88.8 (22.7) | 19 | 85.3 (20.9) | 33 | 89.1 (21.2) | 14 | 87.1 (21.2) | 66 |
| 8 |  | 80 (22) | 26 | 83.6 (22.1) | 28 | 89 (24.7) | 12 | 83.2 (22.4) | 66 |
| 12 |  | 81 (25.6) | 21 | 85.5 (24.4) | 25 | 84.5 (24.9) | 12 | 83.7 (24.6) | 58 |
| **Self-Compassion** | | | |  | |  | |  | |
| 0 |  | 2.7 (0.7) | 36 | 2.5 (0.8) | 41 | 2.4 (0.7) | 19 | 2.5 (0.7) | 96 |
| 4 |  | 2.9 (0.6) | 19 | 2.9 (0.8) | 33 | 2.6 (0.7) | 14 | 2.8 (0.7) | 66 |
| 8 |  | 3.1 (0.7) | 26 | 3 (0.7) | 27 | 2.9 (0.9) | 12 | 3 (0.7) | 65 |
| 12 |  | 3.1 (0.8) | 20 | 2.9 (0.8) | 25 | 2.9 (0.8) | 12 | 2.9 (0.8) | 57 |
| **Decentering** | | | |  | |  | |  | |
| 0 |  | 33 (5.7) | 35 | 32.5 (5.7) | 41 | 29.8 (4) | 19 | 32.2 (5.5) | 95 |
| 4 |  | 34.1 (5.4) | 19 | 35.2 (5.8) | 33 | 33 (5.2) | 14 | 34.4 (5.6) | 66 |
| 8 |  | 36.5 (6.1) | 26 | 35.3 (5.2) | 28 | 35.6 (6.4) | 12 | 35.8 (5.7) | 66 |
| 12 |  | 35.5 (6.9) | 22 | 33.6 (5.7) | 26 | 34.1 (3.9) | 13 | 34.4 (5.8) | 61 |
|  | | | |  | |  | |  | |

^a^MBCT-R = Mindfulness-Based Cognitive Therapy for Resilience ^b^iCBT = internet Cognitive Behavioral Therapy

^c^CHA-MW = Cambridge Health Alliance (CHA) Mindwell

**Figure S1.** Marginal estimates of CAT-MH depression score by study week, group and completer status.

Legend: Figure shows the mean and standard error of the marginal estimate by study week, group and complete status
